# Global and regional prevalence of metallo-β-lactamases in carbapenem-resistant *Acinetobacter baumannii*: a systematic review and meta-analysis

**DOI:** 10.1093/jac/dkag037

**Published:** 2026-02-05

**Authors:** Stamatis Karakonstantis, Renatos Nikolaos Tziolos, Maria Paneta, Evangelos I Kritsotakis

**Affiliations:** Division of Internal Medicine, School of Medicine, University of Crete, Heraklion, Greece; Department of Internal Medicine & Infectious Diseases, University Hospital of Heraklion, University of Crete, Heraklion, Greece; Department of Internal Medicine & Infectious Diseases, University Hospital of Heraklion, University of Crete, Heraklion, Greece; Department of Internal Medicine & Infectious Diseases, University Hospital of Heraklion, University of Crete, Heraklion, Greece; Laboratory of Biostatistics, School of Medicine, University of Crete, Heraklion, Greece

## Abstract

**Background:**

Emergence and spread of carbapenem-resistant *Acinetobacter baumannii* (CRAB) producing metallo-β-lactamases (MBLs), especially New Delhi MBLs (NDMs), are concerning due to resistance to last-resort antibiotics.

**Methods:**

PubMed and Scopus were queried for studies published between 2020 and 2024 reporting MBL prevalence in CRAB isolates. Risk of bias was assessed across the population, setting and measurement domains. Binomial-Normal mixed-effects models were applied to estimate regional and country-specific weighted MBL and NDM prevalence proportions in CRAB. Subgroup and meta-regression analyses were performed to investigate heterogeneity.

**Results:**

Two hundred thirty-three studies reporting 58 676 CRAB isolates were analysed. The global MBL prevalence in CRAB was 5.3% [95% confidence interval CI), 3.3%–8.2%]. Regional variability explained a substantial portion of heterogeneity in meta-regression (*R*^2^ = 35%). MBL prevalence in CRAB was highest in the Eastern Mediterranean (42.1%; 95% CI, 28.7%–56.8%) and African (36.1%; 95% CI, 12.2%–69.8%) regions, moderately high in South-East Asia (17.9%; 95% CI, 9.6%–30.9%), and low (<1%) in Europe, the Americas and the Western Pacific region. MBL prevalence in CRAB was higher in studies conducted during 2020–2024 than during 2012–2019 (7.2% versus 4.2%; adjusted odds ratio 2.3, *P* = 0.024). NDM was the dominant MBL in CRAB, with a global prevalence of 1.7% (95% CI, 1.1%–2.7%) in 218 studies.

**Conclusions:**

Although its global prevalence is low, MBL-producing CRAB is common in specific regions and countries, threatening the utility of new antibiotics. Sustained surveillance, rigorous infection control and antimicrobial stewardship are required to preserve the activity of last-resort antimicrobials.

## Introduction

Carbapenem-resistant *Acinetobacter baumannii* has become a significant and difficult-to-treat nosocomial pathogen. Resistance even to last-resort antimicrobials is increasingly reported globally.^1,2^ The morbidity and mortality associated with *A. baumannii* are widely acknowledged, despite earlier controversies.^3,4^ Treatment options for carbapenem-resistant *A. baumannii* are limited.^5–8^ Polymyxins can be considered, but resistance to polymyxins is increasing^9,10^ and treatment outcomes with polymyxin-based regimens are suboptimal compared to newer options like sulbactam/durlobactam^11,12^ and cefiderocol.^12,13^ High-dose ampicillin/sulbactam is another possibility, but resistance is frequent in some settings,^10^ and it is no longer preferred when sulbactam/durlobactam is available.^7,8^ Additionally, various synergistic combinations have been tried,^14,15^ but clinical data on their safety and efficacy are limited.^16,17^

Cefiderocol and sulbactam/durlobactam are promising options that may improve patient outcomes,^11–13^ but their availability is currently limited, even in settings where *A. baumannii* is highly prevalent. Furthermore, sulbactam/durlobactam is not active against metallo-β-lactamase (MBL)-producing CRAB,^18^ while New Delhi MBLs (NDMs) have been associated with decreased susceptibility to cefiderocol.^2,19^ With the increasing use of sulbactam/durlobactam and cefiderocol, there is a risk of selecting MBL-producing *A. baumannii* strains resistant even to these last-resort treatment options. Therefore, monitoring the evolving global epidemiology of MBL-producing *A. baumannii* is crucial.

Herein, we report a systematic review and meta-analysis of the global and regional pooled prevalence of MBLs in clinical CRAB isolates. As NDM is the predominant MBL type among CRAB isolates globally,^20^ and the most concerning in terms of resistance to last resort antibiotics, we also report on the prevalence of NDM-type MBLs in CRAB.

## Material and methods

This systematic review with meta-analysis was preregistered with the International Prospective Register of Systematic Reviews (PROSPERO, CRD42024629402) and is reported according to the Preferred Reporting Items for Systematic reviews and Meta-Analyses (PRISMA) 2020 reporting guideline.

### Search strategy, information sources and eligibility

PubMed and Scopus were searched from inception to 21 October 2024 to identify studies reporting the proportion of MBLs among clinical CRAB isolates. PubMed was queried for (Acinetobacter OR baumannii) AND (metallo OR carbapenemase* OR MBL OR NDM), and Scopus was searched for (TITLE (acinetobacter OR baumannii) AND TITLE-ABS-KEY (metallo OR carbapenemase* OR mbl OR ndm)). No language exclusion criteria were applied. Case reports, studies with fewer than 10 CRAB isolates and studies selecting CRAB isolates based on MBL production were excluded. Studies of non-*A. baumannii* isolates were also excluded, recognizing the limitation that available identification methods cannot differentiate between the various species within the *A. baumannii-Calcoaceticus* complex. Due to the substantial number of eligible articles and a focus on the recent epidemiology, we reviewed articles published in the last five years (since 2020).

### Deduplication, screening and data extraction

Deduplication and screening of the articles were conducted in the Rayyan online platform by one author.^21^ Duplicate articles with >97% similarity percentage or the same ‘DOI’ were resolved using Rayyan’s ‘Auto Resolver’ function. De-duplication of the remaining articles was conducted manually.

Data were extracted from each study by one author using an Excel template and were subsequently confirmed by a second author. Disagreements were resolved through discussion and consensus. The following data were collected: year of publication, period when the *A. baumannii* isolates were collected, sampling method for selecting CRAB isolates for carbapenemase testing, country/-ies and number of centres where the isolates were collected, method of *A. baumannii* identification, definition of carbapenem resistance, method(s) of carbapenemase detection, list of carbapenemases that were sought (if only specific carbapenemase types were sought), study population characteristics (outpatients/inpatients/mixed, ICU/non-ICU/mixed, paediatric/adults/elderly/mixed), total number of *A. baumannii* isolates, total number of CRAB isolates, number of CRAB isolates assessed for the presence of carbapenemases, number of CRAB isolates positive for MBL-type carbapenemases of any subtype and number of CRAB isolates positive for NDM subtype carbapenemases.

### Risk of bias assessment

A customized tool for assessing risk of bias was developed by adapting the Joanna–Briggs critical appraisal tool for prevalence studies to suit the context of the present review.^22,23^ The developed tool is described in detail in the first section of the Supplement. Briefly, risk of bias was assessed in two domains applicable to this review: (a) population and setting (examining the sampling plan, sample size, description of subjects and clinical setting and coverage bias), and (b) condition measurement (examining the validity and standardization of the method for carbapenemase detection). Studies with moderate or high risk of bias in either domain were categorized as moderate-to-high risk overall, and those with low risk of bias in both domains were classified as low risk overall.

### Outcomes

The outcome endpoints of interest were as follows: (1) the prevalence of clinical CRAB isolates that were positive for MBL-type carbapenemases, (2) the prevalence of clinical CRAB isolates producing MBL-type carbapenemases that were positive for NDM-subtype carbapenemases.

### Meta-analysis

Global, regional and country-specific prevalence proportions of MBL-type and NDM-type carbapenemases among CRAB isolates were estimated as weighted averages from individual study data using a mixed-effects logistic regression model.^24,25^ The model assumes a binomial distribution for the number of carbapenemase-producing CRAB isolates in each study and a normally distributed random effect (log odds ratio) across studies. Maximum likelihood estimation and the Laplace approximation for integration were applied.^24,25^ A pooled prevalence estimate was obtained as a weighted mean. Weighting is implicit: studies with lower variability contribute more to the model’s likelihood function and exert greater influence on the pooled estimate. The Binomial-Normal mixed-effects model correctly manages proportions near 0% or 100% (when variance tends to zero and classic inverse variance weights are problematic) and maintains the confidence limits for the pooled proportion within the zero to one range. The resulting confidence interval contains highly probable values for the population-averaged (pooled) prevalence proportion. Whenever computational problems arose, pooled prevalence proportions were estimated using a random-effects inverse-variance model after applying the Freeman-Tukey double-arcsine transformation.^26^

Caterpillar forest plots were constructed to illustrate the distribution of prevalence proportions across studies, with a 95% confidence interval (CI) for each study calculated using Wilson's score method. Higgins-Thompson's I² statistic was used as a summary index of variability across the studies that cannot be attributed to sampling error. Because I² is usually high and may not be informative for prevalence data,^27^ the between-study variance (τ^2^) and its respective 95% prediction interval (PI) were also reported. The PI describes the expected range of MBL prevalence in CRAB isolates in new studies or settings.^28^ The geographic distributions of MBL and NDM proportions in CRAB were illustrated with choropleth maps based on the latest country boundaries published by the World Bank.

Multivariable meta-regression analysis using the Binomial-Normal mixed-effects model was employed to examine sources of heterogeneity. Adjusted odds ratios and their 95% CІs were calculated to summarize the strength and direction of associations between study-level covariates and the prevalence of MBL carbapenemase production in CRAB. A covariate-specific *R*^2^ statistic was calculated as the portion of between-study variance reduced after including that covariate in the multivariable model. Moreover, pooled estimates of MBL carbapenemase production prevalence were obtained for each covariate level using subgroup analysis. *A priori* selected covariates were examined, including the year when data collection ended (examined both as a dichotomous indicator before and after 2020, and as a continuous variable using a restricted cubic spline^29^), geographical region [according to the World Health Organization (WHO) classification], multicounty setting (yes/no), multicentre setting (yes/no) and risk of bias (classified as either low or moderate-to-high).

To assess publication bias, we examined asymmetry in Doi plots of normal-quantiles (*z*-scores) against logit-transformed MBL and NDM prevalence proportions in CRAB.^30^ In addition, we calculated the LFK index (values close to zero indicate symmetry).^30^

STATA (Version 19; StatCorp, College Station, TX, USA) was used for all analyses. A value of *P* < 0.05 was considered statistically significant.

## Results

### Study characteristics

**Figure 1.**
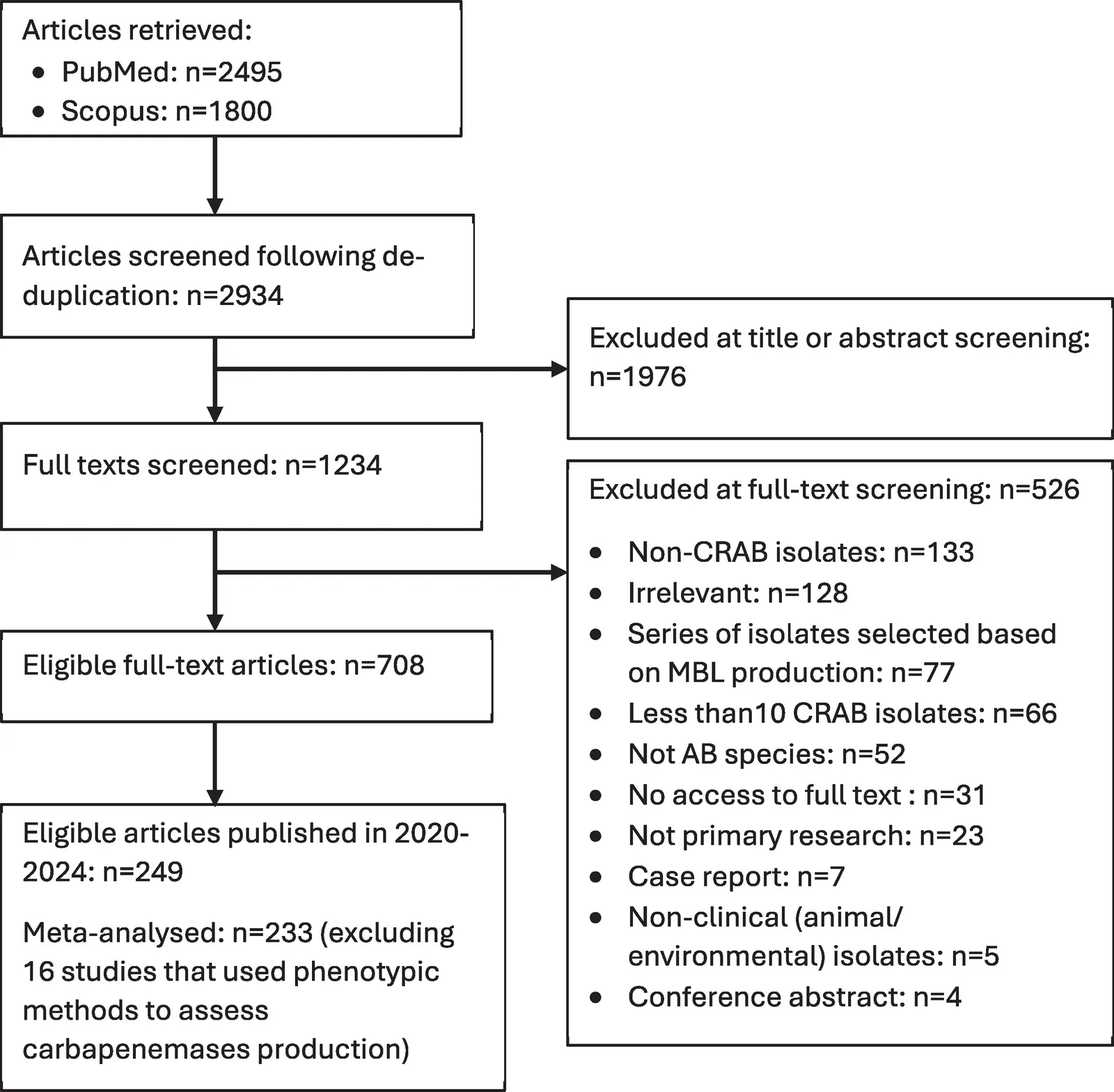
Flowchart of the identification, screening, and inclusion process. AB, *Acinetobacter baumannii*; CRAB, carbapenem-resistant *Acinetobacter baumannii*; MBL, metallo-β-lactamase.

Figure 1 depicts the review flow chart. In total, *n* = 249 eligible studies were assessed (the list of the studies is available in the Supplement). Study characteristics are summarized in Table S1 (available as Supplementary data at *JAC* Online). Based on the period when data collection ended in each study, 57% of the studies collected data before 2020, while 43% collected data between 2020 and 2024. About half (52%) were multicentre studies, and a minority (6%) were conducted in more than one country. A considerable number of studies were retrieved from the Eastern Mediterranean region (*n* = 65), the European region (*n* = 54), South-East Asia (*n* = 41), the Western Pacific region (*n* = 36) and the Americas (*n* = 34). In contrast, only 9 studies originated in the African region, and 10 were multiregional. Patient-related data (clinical care setting, intensive care and age) were largely unreported. Most studies (61%) collected data exclusively from hospitalized patients, but 27% did not clearly report on their source population. In 93% of the studies, carbapenemase types were identified using molecular methods. Phenotypic methods, which may severely overestimate MBL production, were used in 16 studies (6%). In one study, carbapenemase type was assessed by immunochromatography.

### Risk of bias

The majority (69%) of the studies were assessed as moderate-to-high risk of bias (Tables S2 and S3), primarily due to potential biases in the population and setting domains. Inadequate sample size (97%), inadequate description of setting and subjects (42%) and inappropriate or unclear sampling method (33%) were the key issues contributing to the risk of bias.

#### Global prevalence of MBL and NDM in CRAB

In all, 4534 MBL producers were identified among 58 676 CRAB isolates by molecular methods in 233 studies, leading to a globally pooled prevalence estimate for MBL-producing CRAB of 5.3% (95% CI, 3.3%–8.2%). In addition, 16 studies reported 409 MBL producers among 703 CRAB isolates (pooled weighted prevalence 65.4%; 95% CI 17.9%–92.4%; Figure S1) using unreliable phenotypic methods; these studies were excluded from further meta-analysis.

NDM-subtype carbapenemases were reported in 2301 of 57 147 CRAB isolates in 218 studies, for a pooled prevalence of 1.7% (95% CI, 1.1%–2.7%; Figure S2). Globally, MBL CRAB isolates were almost entirely NDMs (pooled weighted proportion 100%, 95% CI, 99.8%–100%, in 148 studies reporting at least one MBL CRAB isolate; Figure S3). Studies examining NDM subtypes predominantly reported the NDM-1 subtype (948 of 980 NDM CRAB isolates in 75 studies; Figure S4).

### Heterogeneity

**Figure 2.**
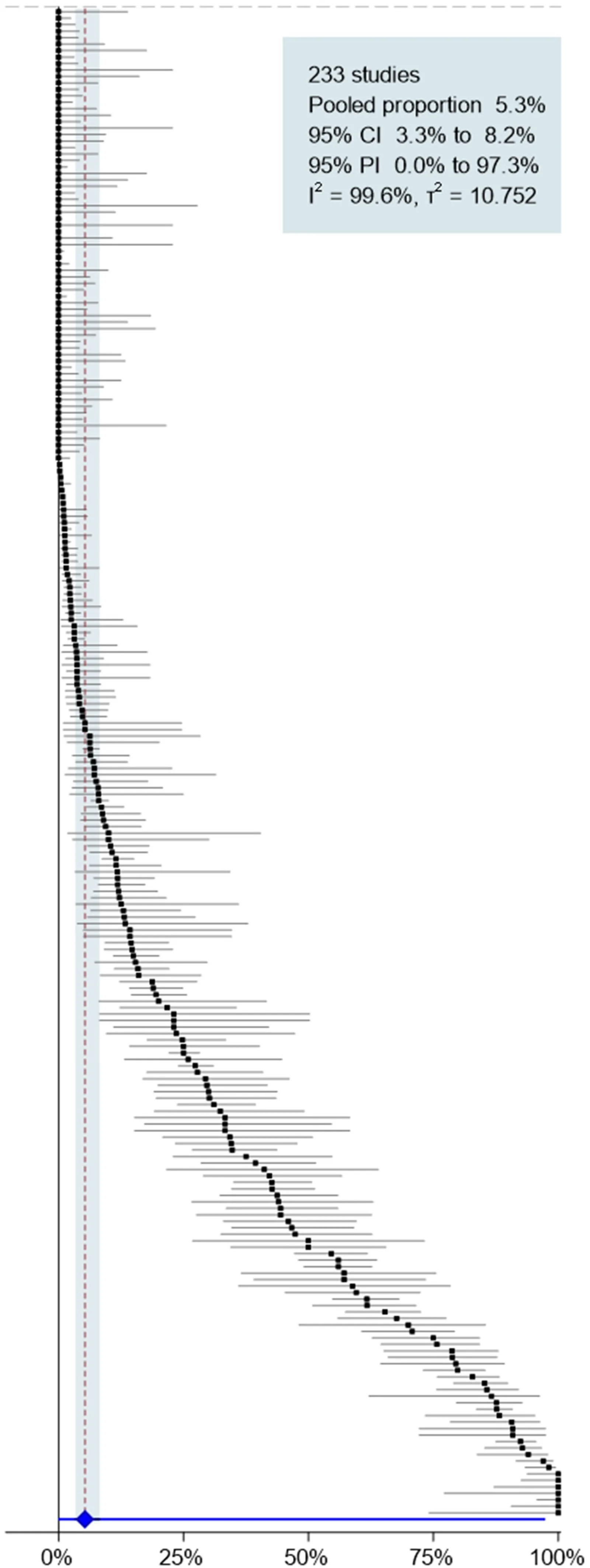
Caterpillar plot of the random-effects meta-analysis of the global prevalence of metallo-β-lactamases (MBL) in carbapenem-resistant *A. baumannii.* Black squares and grey horizontal lines are point estimates and 95% confidence intervals, respectively, of MBL prevalence percentages in individual studies. The blue diamond centre represents the global population-averaged (pooled prevalence) estimate. The diamond's length and the respective bluish-grey vertical area indicate the 95% CI for the pooled prevalence. The blue horizontal line continuing through the diamond indicates the 95% prediction interval (PI) for the expected prevalence in new studies.

**Figure 3.**
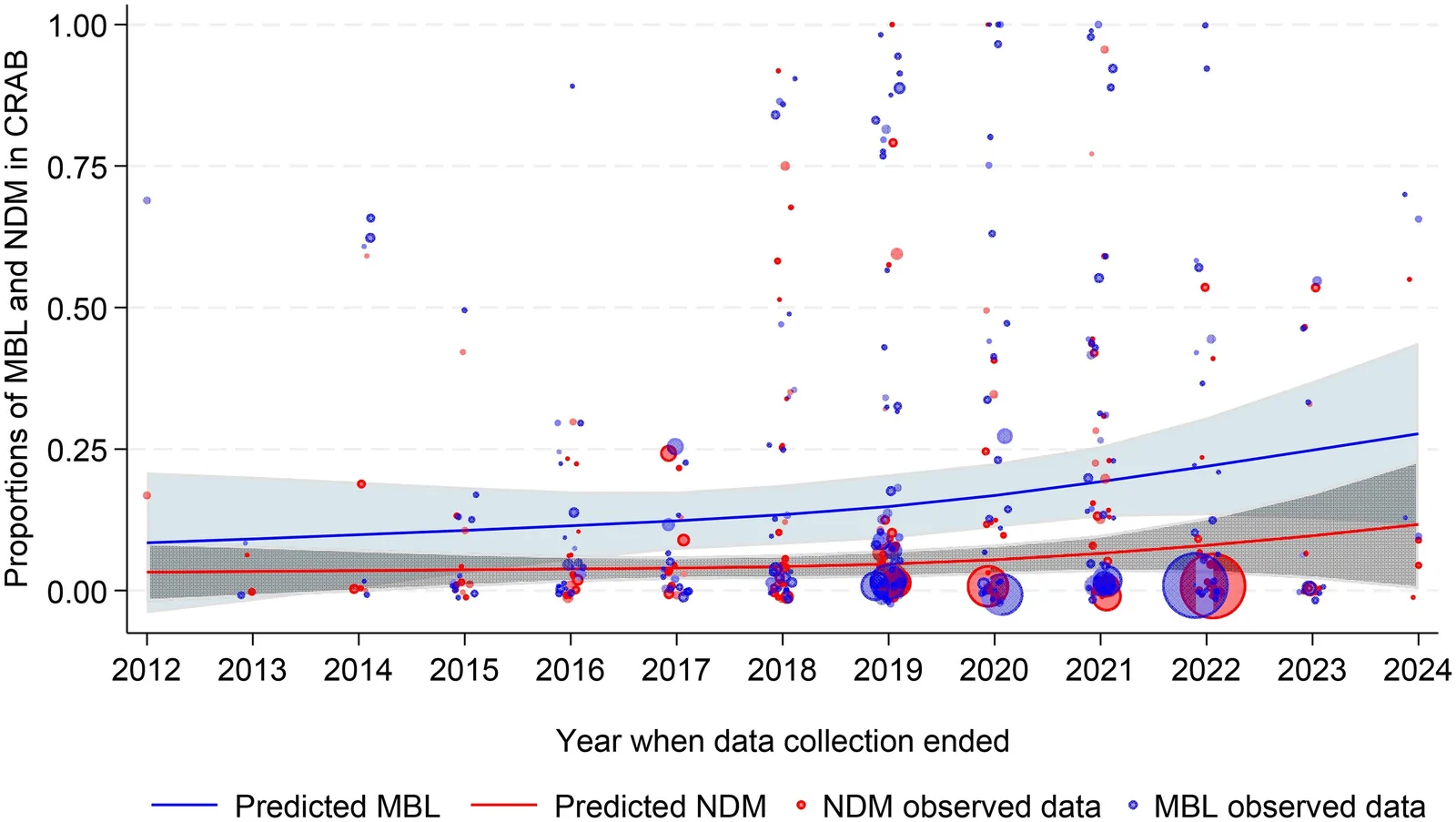
Time trend in the annual global prevalence of MBL- and NDM-producing CRAB isolates. The bubbles represent data points from individual studies; their sizes are proportional to sample sizes. A restricted cubic spline was used to model the independent effect of the year of study conduct on the proportions of MBL CRABs (solid blue line) and NDM CRABs in particular (solid red line) in a multivariable Binomial-Normal mixed-effects model, adjusting for WHO region, multicounty setting, multicentre setting and risk of bias classification. The bluish-grey and violet shaded areas represent the corresponding pointwise 95% confidence intervals. MBL, metallo-β-lactamase; NDM, New Delhi MBL; CRAB, carbapenem-resistant *A. baumannii*.

As evident in Figure 2, the studies were characterized by maximal heterogeneity in reported prevalence rates (*I*^2^ = 99.6%; PI for MBL prevalence in CRAB in a new study, 0% to 97.3%). Multivariable meta-regression (Table 1) revealed that regional variation (WHO regions) explained a substantial portion of the observed heterogeneity in reported prevalence rates (*R*^2^ = 35%). In addition, MBL prevalence in CRAB was higher in studies conducted during 2020–2024 than during 2012–2019 (pooled estimates 7.2% versus 4.2%; aOR = 2.3, *P* = 0.024), independently of other study-level covariates. The time trend in the annual global proportions of MBL- and NDM-producing CRAB isolates is illustrated in Figure 3.

**Table 1.** Subgroup and multivariable meta-regression analyses of the associations between study-level characteristics and the prevalence of metallo-β-lactamases in carbapenem-resistant A. baumannii

Study characteristic	Subgroup	*n*	MBL/CRAB (CI), %	aOR (CI)	*P*	R^2^
Period*^a^*	2012–2019	132	4.2 (1.7–6.7)	Ref.	0.024	3.5%
	2020–2024	101	7.2 (2.5–11.9)	2.34 (1.12–4.92)		
WHO region	EUR	50	0.9 (0.3–2.3)	Ref.		35.0%
	AMR	31	0.4 (0.0–4.2)	1.48 (0.37–5.87)	0.579	
	WPR	36	0.4 (0.1–3.0)	1.11 (0.30–4.07)	0.877	
	SEAR	37	17.9 (9.6–30.9)	23.9 (7.05–80.8)	<0.001	
	AFR	7	36.1 (12.2–69.8)	76.3 (8.85–658.4)	<0.001	
	EMR	62	42.1 (28.7–56.8)	80.0 (26.5–241.9)	<0.001	
	Multiregional	10	1.1 (0.0–3.4)	2.15 (0.07–63.87)	0.658	
Multi-country setting	No	219	6.1 (3.3–8.8)	Ref.	0.855	0.1%
	Yes	14	0.9 (0.0–2.4)	0.76 (0.04–14.86)		
Multicentre setting	No	111	8.4 (3.3–13.6)	Ref.	0.731	0.5%
	Yes	122	3.6 (1.4–5.7)	0.87 (0.41–1.88)		
Risk of bias	Moderate-to-high risk	157	6.0 (2.8–9.1)	Ref.	0.846	<0.1%
	Low risk	76	4.0 (0.8–7.2)	1.48 (0.03–77.9)		

*n* number of studies; MBL/CRAB, prevalence of metallo-β-lactamase producers in carbapenem-resistant isolates; CI, 95% confidence interval; aOR, adjusted odds ratio; EUR, European region; AMR, Region of the Americas; WPR, Western Pacific Region; SEAR, South-East Asia Region; EMR, Eastern Mediterranean Region; AFR, African Region.

^*a*^Period was defined by the year when data collection ended in each study.

In the univariate analysis, MBL prevalence in CRAB appeared to be higher in single-country and single-centre studies (Table S4), but these associations were not statistically significant in the multivariable regression model. There was no evidence of significant variation in prevalence estimates across risk-of-bias categories in either the univariate or multivariable analyses.

### Regional and country-specific prevalence

Data on the overall MBL and the NDM-subtype prevalence rates in CRAB clinical isolates were available from studies in 14 of 22 countries (64%) in the Eastern Mediterranean region, 21 of 53 countries (40%) in the European region, 7 of 11 countries (64%) in the South-East Asia region, 7 of 35 countries (20%) in the Americas, 8 of 27 countries (30%) in the Western Pacific and 5 of 47 countries (11%) in the African region.

MBL prevalence in CRAB was exceedingly high in the Eastern Mediterranean (42.1%; 95% CI, 28.7–56.8%) and African (36.1%; 95% CI, 12.2–69.8%) regions, moderately high in South-East Asia (17.9%; 95% CI, 9.6–30.9%) and low (<1%) in the European region, the Americas and the Western Pacific region. The high prevalence in the Eastern Mediterranean, African and South-East Asian regions was likely driven by non-NDM-type MBLs. Looking specifically at NDM-type MBLs, their prevalence in these regions was 8.2% (95% CI 4.2%–15.5%), 26.0% (95% CI 7.0%–62.0%) and 12.2% (95% CI 7.1%–20.2%), respectively.

**Figure 4.**
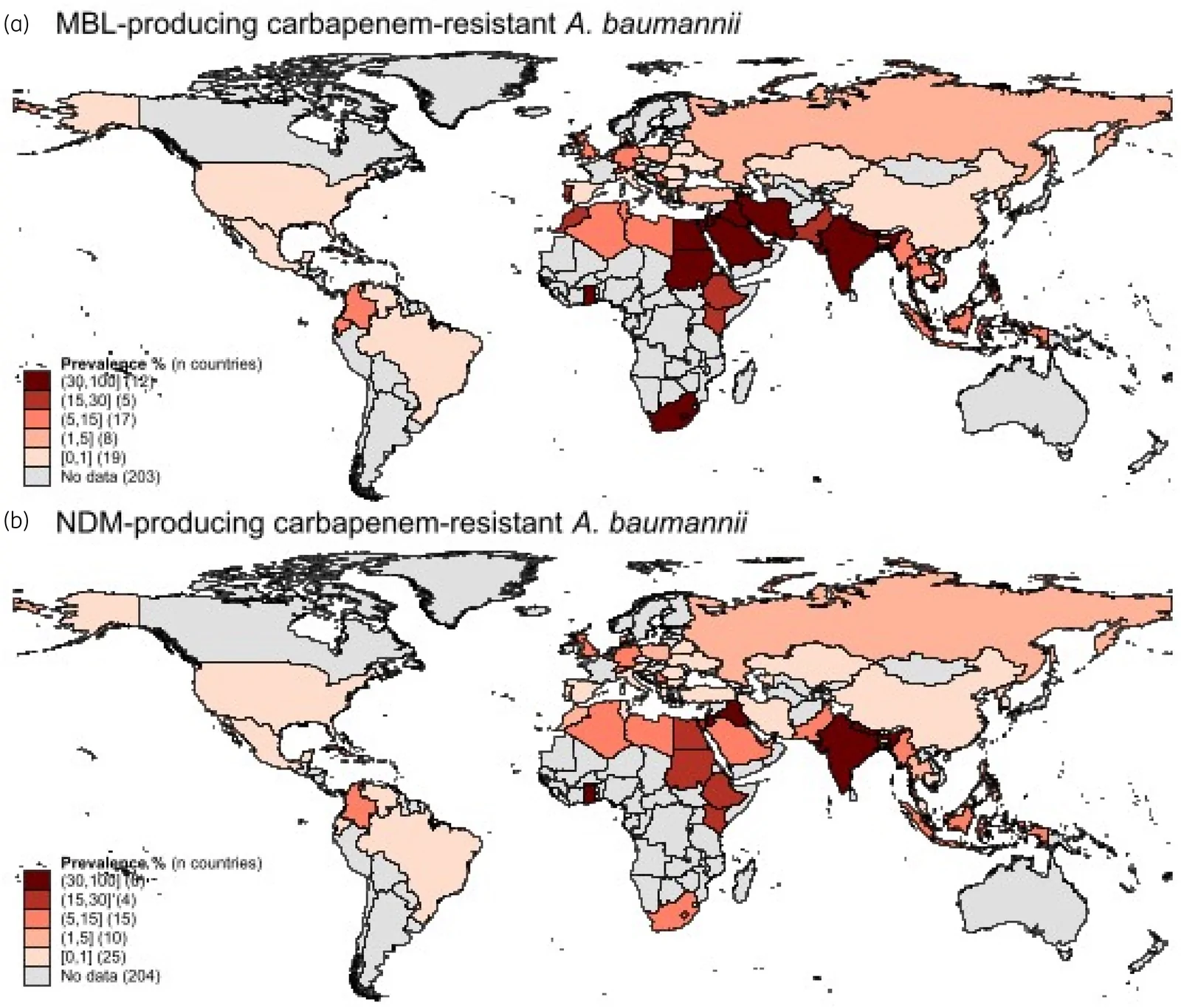
Global distribution of the country-specific estimates of the prevalence of MBL- and NDM-producing carbapenem-resistant *A. baumannii* clinical isolates.

Figure 4 shows the global distributions of country-specific prevalence estimates for MBL- and NDM-producing CRAB. The MBL prevalence estimates are listed in supplementary Tables S5-S10 per region and country, whereas NDM-subtype prevalence estimates are given in Tables S11-S16.

In the Eastern Mediterranean region, MBL prevalence ranged from 1.4% to 96.9% of the reported CRAB clinical isolates. Most studies originated from Iran (*n* = 18) and Egypt (*n* = 17), with pooled MBL prevalence proportions of 56.1% (95% CI, 27.7–81.0%) and 37.9% (95% CI, 25.6–52.0%), respectively. Exceedingly high MBL CRAB prevalence was reported in Saudi Arabia (96.9%; 95% CI, 5.4–100.0%; 2 studies), Bahrain (94.0%; 95% CI, 83.8–97.9%; 1 study) and Iraq (85.2%; 95% CI, 47.7–97.3%; 3 studies). The highest NDM CRAB prevalences were reported in Iraq (89.7%, 95% CI 11.9%–99.8%, 2 studies), Jordan (42.0%, 95% CI 6.2%–88.9%, 1 study), Sudan (25.0%, 95% CI 14.2%–40.2%, 1 study), Egypt (24.6%, 95% CI 15.5%–36.7%, 17 studies) and Saudi Arabia (12.3%, 95% CI 6.8%–21.3%, 1 study).

In Europe, the reported MBL prevalence proportions in CRAB ranged from <0.1% in most countries (Austria, Belarus, Bulgaria, Croatia, Italy, Kazakhstan, Romania, Spain and Ukraine) to 18.8% (95% CI, 12.2–27.7%; 1 study) in Portugal. Relatively high regional MBL prevalence (100% NDM type) was documented in Serbia (12.8%; 95% CI, 3.9–34.7%; three studies), Switzerland (9.4%; 95% CI 5.2–16.5%; one study), Israel (6.3%; 95% CI 4.9–8.1%; one study), the United Kingdom (6.2%; 95% CI 1.1–28.3%; one study) and Germany (6.0%; 95% CI 2.8–10.1%; six studies).

The largest number of studies in the South-East Asia region was from India (18 studies), where the highest regional MBL prevalence in CRAB was observed (43.8%; 95% CI, 27.8–61.1%). NDM CRAB prevalence in India was 30.3% (95% CI 18.6%–45.4%) based on 16 studies. Exceedingly high MBL CRAB prevalence was also observed in Nepal (37.6%; 95% CI, 28.7–47.4%; 2 studies, all NDMs).

In the Americas, most data were derived from Brazil (nine studies) and the USA (eight studies), with a <1% prevalence of MBL and NDM CRAB in both countries. Relatively higher MBL prevalence was reported in Cuba (11.8%, 95% CI 7.9%–17.3%, all NDM, 1 study) and Colombia (7.9%, 95% CI 2.7%–20.8%, 67% NDM, one study).

Most studies from the Western Pacific region originated in China (22 studies) and reported a <1% prevalence of MBL and NDM in CRAB. Exceedingly high MBL prevalence (all NDMs) was reported in one study in Fiji (42.7%, 95% CI 34.6%–51.3%). High MBL prevalence was also reported in one study in Vietnam (14.5%, 95% CI 9.3%–22.0%, MBL subtype unknown).

Only seven studies were retrieved from the WHO African region, yielding a pooled estimate of MBL prevalence in CRAB of 36.1% (95% CI, 12.2%–69.8%). The highest MBL CRAB prevalence was reported in one study in Ghana (90.9%; 95% CI, 72.2%–97.5%), and the lowest was seen in one study in Algeria (14.3%; 95% CI, 5.0%–34.6%). Most MBL isolates were of the NDM subtype.

### Publication bias

A visual examination of the Doi plots in Figure S5, along with the corresponding LFK index values, suggests asymmetry, indicating that studies reporting higher MBL prevalence in CRAB and, to a greater extent, NDM CRAB prevalence, were slightly less frequently published than would be expected by chance. Stratification by WHO region indicated that this bias primarily arose from studies conducted in Europe, the Western Pacific region and South-East Asia (Figures S6 and S7).

## Discussion

This systematic review and meta-analysis demonstrated a low global prevalence of MBLs among CRAB clinical isolates, with the NDM enzyme (predominantly NDM-1) being the most common. Significant regional variation was observed, with remarkably high MBL prevalence in the Eastern Mediterranean, African and South-East Asia regions, likely driven by non-NDM-type MBLs. In other WHO regions, the pooled prevalence of MBL- and NDM-producing CRAB is low (<1%), but certain countries and specific centres have reported high rates, even within these low-prevalence regions. Moreover, MBL prevalence was higher in studies reporting more recent CRAB isolates, and meta-regression revealed a slowly but steadily increasing global MBL CRAB prevalence over time, independently of other covariates.

Similar to our findings, previous region-specific and/or non-systematic reviews have noted high MBL prevalence proportions in certain regions, particularly Asia and Africa.^20,31–33^ Recent studies have also highlighted the potential for clonal dissemination of NDM-producing CRAB strains,^34–36^ including cross-border spread.^37^ These findings raise concern regarding the potential spread of MBL-producing CRAB strains, which could threaten the activity of recently approved antibiotics, including cefiderocol and sulbactam/durlobactam. Sulbactam/durlobactam is inactive against MBL-producing CRAB,^18^ and NDMs may reduce susceptibility to cefiderocol.^2,19^ These antibiotics are last-resort options to treat infections caused by extensively drug-resistant *A. baumannii,* which is a major cause of nosocomial infections, with significant burden on patients and healthcare facilities in several regions and countries, and widely considered an urgent antimicrobial resistance threat.^38^ Other therapeutic choices, such as polymyxins, tigecycline and sulbactam, have significant drawbacks, including nephrotoxicity, gastrointestinal disturbances and limited effectiveness in certain cases.^6,11–13^

An interesting finding of this study is the steadily increasing global prevalence of MBLs in CRAB over time, even preceding the use of the recently approved cefiderocol and sulbactam/durlobactam. We hypothesize that the MBL prevalence in CRAB might have been driven by an increasing use of high-dose sulbactam, which has emerged as a preferred backbone option for treating CRAB infections.^1,7,39^ Sulbactam MIC is higher in NDM-producing CRAB strains,^40,41^ which may explain why sulbactam-based treatment regimens may select for these strains. Another hypothesis is that increasing MBL prevalence in CRAB may have been driven by increasing MBL prevalence in Enterobacterales,^42^ affected by new β-lactam-β-lactamase antibiotics and potential interspecies transfer of MBL genes.^43,44^ To the best of our knowledge, no studies have evaluated the plausibility of these hypotheses for the recent increase in NDM-producing *A. baumannii* isolates.

Strengths of this study include the systematic and comprehensive evaluation of the prevalence of MBL carbapenemases in CRAB clinical isolates and their regional and temporal variation through appropriate meta-analytical methods. However, critical challenges and limitations should be considered when interpreting our findings.

Between-study variability, or heterogeneity, is the primary source of uncertainty for meta-analysis, particularly when investigating the effects of interventions. However, substantial heterogeneity is common and should be expected in prevalence estimates due to differences in the time, place and setting in which the individual studies were conducted.^45,46^ The considerable heterogeneity observed in this study reflects the actual global pattern of MBL prevalence in CRAB and should not be viewed as a limitation. Instead, it is a naturally occurring phenomenon that provides an opportunity to gain deeper insights into the epidemiology and diverse global burden of antimicrobial resistance. Following widely accepted methodological standards,^45,46^ we estimated weighted averages of global, regional and county-specific prevalence proportions using a random-effects model. Additionally, we investigated sources of heterogeneity through subgroup and meta-regression analyses. The analysis illustrated an increasing prevalence of MBL- and NDM-producing CRAB isolates over time and highlighted that regional variability is a primary driver of the observed heterogeneity. However, considerable variation within the regions remained unexplained by the available study-level covariates. Despite our protocol, we were unable to investigate additional sources of heterogeneity related to the clinical setting (such as type and level of care, mean patient age, clinical speciality and site of infection) because the studies were largely unclear about the exact patient care setting from which their data were sourced. Moreover, regional and country-specific pooled estimates were based on a subset of countries within each region and, in some cases, on a small number of studies and/or institutions. Therefore, these estimates should be interpreted with caution, given the uncertainty (as reflected in wide PIs) arising from limited data in the contemporary literature for some regions. Still, the country-specific estimates provided in this study, with acknowledged uncertainty, offer possible regional targets for alerted surveillance of antimicrobial resistance in *A. baumannii*. Grading the certainty of the evidence is particularly challenging in systematic reviews of prevalence, and there is no formal guidance for this.^47^ We noted evidence that studies with high MBL and/or NDM prevalence proportions in CRAB may have been less frequently submitted for publication, especially in Europe and the Western Pacific region, where the reported pooled prevalence rates might have been underestimated in this review. Additionally, we only examined the prevalence of NDMs among the various types of MBL producers, acknowledging the possibility of high non-susceptibility to cefiderocol in NDM-producing CRAB.^2,19^ While NDM is the predominant MBL type among CRAB isolates globally, other MBL enzymes, such as the VIM (Verona integron-encoded MBL) and IMP (imipenemase), have been reported in specific regions and countries,^20,31,48^ but were not sought in this review.

In conclusion, despite a low global prevalence of MBL CRAB clinical isolates, a concerningly high prevalence is observed in certain countries and institutions. The increasing use of new antibiotics, particularly cefiderocol and sulbactam/durlobactam, may exert selective pressure and facilitate the spread of such strains. Therefore, continued and sustained surveillance of the global and regional epidemiology of *A. baumannii* infections, rigorous infection control measures and antimicrobial stewardship are crucial to preserve the activity of new and last-resort antibiotics.

## Supplementary Material

dkag037_Supplementary_Data

## Access to data

The raw data that support the findings of this study are available on request from the corresponding author.

## Supplementary data

Figures S1 to S7 and Tables S1 to S16 are available as Supplementary data at *JAC* Online.
