# Supplementary material for: Global and regional prevalence of metallo-β-lactamases in carbapenem-resistant Acinetobacter baumannii: a systematic review and meta-analysis
Source: J Antimicrob Chemother. 2026 Feb 5;81(3):dkag037. doi: 10.1093/jac/dkag037 (PMC13496038; doi:10.1093/jac/dkag037)
Supplement: Supplementary file 1 [file dkag037_supplementary_data.docx]

**Online Supplement to**

**Global and regional prevalence of metallo-β-lactamases in carbapenem-resistant *Acinetobacter baumannii*: a systematic review and meta-analysis**

Stamatis Karakonstantis ^1,2^, Renatos-Nikolaos Tziolos ^2^, Maria Paneta ^2^, Evangelos I. Kritsotakis ^3, *^

Affiliations:

^1^ Division of Internal Medicine, School of Medicine, University of Crete, Heraklion, Greece

^2^ Department of Internal Medicine & Infectious Diseases, University Hospital of Heraklion, University of Crete, Heraklion, Greece

^3^ Laboratory of Biostatistics, School of Medicine, University of Crete, Heraklion, Greece

** Correspondence* [e.kritsotakis@uoc.gr](mailto:e.kritsotakis@uoc.gr)

Contents

[**Customised tool for risk of bias assessment**	4](#_Toc215476196)

[Methodology for assessing sample size adequacy	5](#_Toc215476197)

[**Table S1.** Summary table of study characteristics (n = 249)	7](#_Toc215476198)

[**Table S2.** Summary table of risk of bias assessment (n = 249)	9](#_Toc215476199)

[**Table S3.** Critical appraisal for risk of bias of reviewed studies	10](#_Toc215476200)

[**Figure S1.** Caterpillar plot of the random-effects meta-analysis of the proportion of MBL production in carbapenem-resistant *A. baumannii* isolates in studies using phenotypic methods for MBL	15](#_Toc215476201)

[**Figure S2.** Caterpillar plot of the random-effects meta-analysis of the proportion of NDM production in carbapenem-resistant *A. baumannii* isolates in studies that sought for NDM using molecular methods	16](#_Toc215476202)

[**Figure S3.** Caterpillar plot of the random-effects meta-analysis of the proportion of NDM production in MBL-producing carbapenem-resistant *A. baumannii* isolates	17](#_Toc215476203)

[**Figure S4**. Caterpillar plot of the random-effects meta-analysis of the proportion of NDM-1 production in NDM A. baumannii isolates in studies that sought for the NDM-1 subtype using molecular methods	18](#_Toc215476204)

[**Table S4.** Subgroup and univariate meta-regression analyses of the associations between study-level characteristics and the prevalence of metallo-β-lactamases in carbapenem-resistant *A. baumannii*	19](#_Toc215476205)

[**Table S5.** Prevalence estimates of the proportion of metallo-β-lactamases in carbapenem-resistant *A. baumannii* in the Eastern Mediterranean region	20](#_Toc215476206)

[**Table S6.** Prevalence estimates of the proportion of metallo-β-lactamases in carbapenem-resistant *A. baumannii* in Europe	21](#_Toc215476207)

[**Table S7.** Prevalence estimates of the proportion of metallo-β-lactamases in carbapenem-resistant *A. baumannii* in the South-East Asia region	22](#_Toc215476208)

[**Table S8.** Prevalence estimates of the proportion of metallo-β-lactamases in carbapenem-resistant *A. baumannii* in the Region of the Americas	23](#_Toc215476209)

[**Table S9.** Prevalence estimates of the proportion of metallo-β-lactamases in carbapenem-resistant *A. baumannii* in the Western Pacific region	24](#_Toc215476210)

[**Table S10.** Prevalence estimates of the proportion of metallo-β-lactamases in carbapenem-resistant *A. baumannii* in the African region	25](#_Toc215476211)

[**Table S11.** Prevalence estimates of the proportion of New Delhi metallo-beta-lactamases (NDM) in carbapenem-resistant *A. baumannii* in the Eastern Mediterranean region	26](#_Toc215476212)

[**Table S12.** Prevalence estimates of the proportion of New Delhi metallo-beta-lactamases (NDM) in carbapenem-resistant *A. baumannii* in the European region	27](#_Toc215476213)

[**Table S13.** Prevalence estimates of the proportion of New Delhi metallo-beta-lactamases (NDM) in carbapenem-resistant *A. baumannii* in the South-East Asia region	28](#_Toc215476214)

[**Table S14.** Prevalence estimates of the proportion of New Delhi metallo-beta-lactamases (NDM) in carbapenem-resistant *A. baumannii* in the Region of the Americas	29](#_Toc215476215)

[**Table S15.** Prevalence estimates of the proportion of New Delhi metallo-beta-lactamases (NDM) in carbapenem-resistant *A. baumannii* in the Western Pacific region	30](#_Toc215476216)

[**Table S16.** Prevalence estimates of the proportion of New Delhi metallo-beta-lactamases (NDM) in carbapenem-resistant *A. baumannii* in the African region	31](#_Toc215476217)

[**Figure S5.** Doi plot and LFK index to assess publication bias in reporting prevalence proportions of MBL-producing (on the left) and NDM-producing (on the right) isolates in carbapenem-resistant *A. baumannii*	32](#_Toc215476218)

[**Figure S6.** Doi plot and LFK index to assess publication bias in reporting prevalence proportions of MBL-producing carbapenem-resistant *A. baumannii* isolates, stratified by WHO region	33](#_Toc215476219)

[**Figure S7.** Doi plot and LFK index to assess publication bias in reporting prevalence proportions of NDM-producing carbapenem-resistant *A. baumannii* isolates, stratified by WHO region	34](#_Toc215476220)

[**List of studies included in the review**	35](#_Toc215476221)

# **Customised tool for risk of bias assessment**

| **Domains/items** | **Risk of bias assessment** |
| --- | --- |
| **Domain 1:**  **Population and setting** | **Low risk of bias**: Answer is “Yes” to at least 3 of 4 items  **Moderate/high risk of bias**: Answer is “No” or “Unclear” to 2 or more items |
| Was an appropriate sampling plan used? | **Yes:** Inclusion of all consecutive isolates (as eligible per study inclusion criteria) or a random sample of all consecutive isolates, and duplicates excluded (same pathogen isolated >1 time from the same patient).  **No**: Non-random or non-consecutive selection of isolates (e.g. convenience, quota or purposive sampling), or any sampling plan without an explicit method for excluding duplicate isolates.  **Unclear**: Information on the sampling design was inadequately or unclearly described. |
| Was the sample size adequate? | **Yes**: The study reported a precision-based calculation for the minimum number of CRAB isolates and/or was conducted with a sufficiently large number of CRAB isolates, to estimate MBL CRAB prevalence with an adequate degree of precision. Precision was deemed sufficient considering the recommendations of Naing et al. [1]. Namely, a precision of 3% was required for MBL CRAB prevalence percentages between 10 and 90%, a precision of 1/2 of the prevalence was required for MBL CRAB prevalences < 10%, and a precision of 1/2 of one minus the prevalence for MBL CRAB prevalences > 90%. Loss or gain in precision related to the sampling design (“design effect”) was also considered. Our approach to estimating the minimum sample size to achieve the desired level of precision was based on the Wilson score interval and is presented further below with examples.  **No:** The study did not report an appropriate sample size calculation or was conducted with an insufficient number of CRAB isolates, which does not provide an adequate degree of precision in estimating MBL CRAB prevalence. |
| Were the study subjects and the setting described in detail? | **Yes:** Clear description of the characteristics of the study population, including patients’ age, setting (inpatients/outpatient/mixed), and type of inpatients (ICU/non-ICU/mixed).  **No**: Any of the above information was missing or unclear. |
| Was the data analysis conducted with sufficient coverage of the identified sample? | **Yes**: No missing data or missing data unlikely to introduce coverage bias or universal testing of CRAB isolates (at least 90%) for carbapenemase production.  **No:** Missing data likely to introduce coverage bias, or selective rather than universal testing of CRAB isolates for carbapenemase production. |
| **Domain 2:**  **Condition measurement (carbapenemase detection)** | **Low risk of bias**: Answer is “Yes” to both items  **Moderate/high risk of bias**: Answer is “No” or “Unclear” to either of the 2 items |
| Were valid methods used for the identification of the condition? | Yes: Molecular methods (e.g. PCR, WGS) or lateral flow immunoassays were used for carbapenemases detection.  No: No method was reported, or EDTA-based phenotypic methods were used for carbapenemases detection. Despite their high sensitivity, EDTA-based phenotypic methods have low specificity, leading to a high false-positive rate [2]. |
| Was the condition measured in a standard and reliable way for all subjects? | Yes: In single-centre studies, the same method for carbapenemase detection was used for all CRAB isolates. In multi-centre or multi-national studies, a reference laboratory conducted all carbapenemase testing or a clearly described standardised protocol for carbapenemase testing was employed across all centres.  No: Carbapenemase detection was variably conducted in different patients or different patient care settings or centres.  Unclear: Standardisation of the carbapenemase detection method was not reported or was ambiguous. |
| **Overall risk of bias assessment** | **Low risk**: Study at low risk of bias in both domains  **Moderate/high risk**: Study at moderate/high risk of bias in either domain |

The tool’s domains and items were adapted from the Joann-Briggs tool for quality assessment of prevalence studies [3, 4] and modified to suit studies of antimicrobial resistance prevalence. **CRAB** = carbapenem-resistant *Acinetobacter baumannii*, **MBL**= metallo-β-lactamase, **WGS**= whole genome sequencing

## Methodology for assessing sample size adequacy

We used the Wilson score method to calculate confidence intervals for prevalence proportions in individual studies [5]. The level of precision, $d$, or the “margin of error” (Wilson interval width of $2d,$) was calculated as previously described [6]. The level of precision, $d$, was deemed sufficient considering the recommendations of Naing et al. [1]. A precision of 3% is required for estimating prevalence proportions between 10% and 90%, a precision of $0.5\times p$ is required for prevalences < 10%, and a precision of $0.5\times(1-p)$ for prevalences > 90%. For studies reporting extremely low (<1%) or extremely high (>99%) prevalence proportions, we fixed the desired precision at 0.25%. For studies that used a complex sampling design introducing a design effect ($deff$), we assumed a $deff=1.15$ for sampling designs involving clustering (when the ICC was not reported or could not be calculated from the study data) and for sampling plans with unequal or unknown selection probabilities. For simple random, systematic and consecutive sampling, we used $deff=1$. For proportionate stratified sampling, we assumed $deff=0.85$ (whenever $deff$ was not reported or could not be calculated from the study data). The table below provides examples of the minimum sample sizes required to achieve adequate precision in estimating a prevalence proportion.

|  |  | $n$ for estimating $p$  with precision $\pm d$ ($\alpha= 0.05$) | | |
| --- | --- | --- | --- | --- |
| $p (\%)$ | $d (\%)$ | SRS | CS | PSS |
| 0 | 0.25 | 764 | 879 | 650 |
| 0.5 | 0.25 | 3233 | 3718 | 2748 |
| 1 | 0.5 | 1606 | 1846 | 1365 |
| 5 | 2.5 | 304 | 349 | 258 |
| 10 | 3 | 387 | 445 | 329 |
| 15 | 3 | 544 | 626 | 462 |
| 20 | 3 | 681 | 783 | 579 |
| 30 | 3 | 893 | 1027 | 759 |
| 40 | 3 | 1021 | 1174 | 868 |
| 50 | 3 | 1063 | 1223 | 904 |
| 60 | 3 | 1021 | 1174 | 868 |
| 70 | 3 | 893 | 1027 | 759 |
| 80 | 3 | 681 | 783 | 579 |
| 85 | 3 | 544 | 626 | 462 |
| 90 | 3 | 387 | 445 | 329 |
| 95 | 2.5 | 304 | 349 | 258 |
| 99 | 0.5 | 1606 | 1846 | 1365 |
| 99.5 | 0.25 | 3233 | 3718 | 2748 |
| 100 | 0.25 | 764 | 879 | 650 |

SRS, simple random sampling; CS, cluster sampling, PSS, proportionate stratified sampling

**References**

[1] Naing L, Nordin RB, Abdul Rahman H, Naing YT. Sample size calculation for prevalence studies using Scalex and ScalaR calculators. *BMC Med Res Methodol*. 2022;22(1):209. Published 2022 Jul 30. doi:10.1186/s12874-022-01694-7.

[2] Bedenić B, Ladavac R, Vranić-Ladavac M, Barišić N, Karčić N, Sreter KB, et al. FALSE POSITIVE PHENOTYPIC DETECTION OF METALLO-BETA-LACTAMASES IN ACINETOBACTER BAUMANNII. Acta Clin Croat. 2019;58:113-8. DOI:<https://doi.org/10.20471/acc.2019.58.01.15>

[3] Migliavaca CB, Stein C, Colpani V, Munn Z, Falavigna M. Quality assessment of prevalence studies: a systematic review. J Clin Epidemiol. 2020;127:59-68. DOI:<https://doi.org/10.1016/j.jclinepi.2020.06.039>

[4] Munn Z, Moola S, Lisy K, Riitano D, Tufanaru C. Methodological guidance for systematic reviews of observational epidemiological studies reporting prevalence and cumulative incidence data. Int J Evid Based Healthc. 2015;13:147-53. DOI:<https://doi.org/10.1097/xeb.0000000000000054>

[5] Agresti, A., & Coull, B. A. (1998). Approximate is Better than “Exact” for Interval Estimation of Binomial Proportions. The American Statistician, 52(2), 119–126. <https://doi.org/10.1080/00031305.1998.10480550>

[6] Krishnamoorthy, K., & Peng, J. (2007). Some Properties of the Exact and Score Methods for Binomial Proportion and Sample Size Calculation. Communications in Statistics - Simulation and Computation, 36(6), 1171–1186. <https://doi.org/10.1080/03610910701569218>

# **Table S1.** Summary table of study characteristics (n = 249)

| **Study characteristic** | **Statistics** |
| --- | --- |
| Year of publication |  |
| 2020 | 45 (18%) |
| 2021 | 65 (26%) |
| 2022 | 42 (17%) |
| 2023 | 47 (19%) |
| 2024 | 50 (20%) |
| Period when data collection ended |  |
| 2012-2019 | 142 (57%) |
| 2020-2024 | 107 (43%) |
| WHO region |  |
| EMR | 65 (26%) |
| EUR | 54 (22%) |
| SEAR | 41 (16%) |
| WPR | 36 (14%) |
| AMR | 34 (14%) |
| AFR | 9 (4%) |
| Multiregional | 10 (4%) |
| Multi-country | 15 (6%) |
| Number of countries | 2 (1 - 55) |
| Multi-centre | 129 (52%) |
| Number of centres | 11 (1 - 448) |
| Patient care setting |  |
| Inpatient care | 153 (61%) |
| Outpatient care | 1 (0%) |
| Mixed | 28 (11%) |
| Not reported | 67 (27%) |
| Intensive care setting |  |
| Yes | 35 (14%) |
| No | 12 (5%) |
| Mixed | 93 (37%) |
| Not reported | 109 (44%) |
| Age group of patients |  |
| Adults | 57 (23%) |
| Children | 8 (3%) |
| Mixed | 61 (25%) |
| Not reported | 122 (49%) |
| Method of selecting CRAB isolates |  |
| Simple random or consecutive sampling | 234 (94%) |
| Convenience or purposive sampling | 5 (2%) |
| Stratified random sampling | 1 (0%) |
| Cluster random sampling | 8 (3%) |
| Unclear | 1 (0%) |
| Number of CRAB isolates | 238 (10 - 15538) |
| Assessed all CRAB for carbapenemases | 226 (91%) |
| Carbapenemase detection method |  |
| Molecular | 232 (93%) |
| Phenotypic | 16 (6%) |
| Immunoassay | 1 (0%) |
| Population and setting risk of bias |  |
| Low | 84 (34%) |
| Moderate-to-high | 165 (66%) |
| Condition measurement risk of bias |  |
| Low | 231 (93%) |
| Moderate-to-high | 18 (7%) |
| Overall risk of bias |  |
| Low | 76 (31%) |
| Moderate-to-high | 173 (69%) |

Summary statistics are n (%) for categorical variables and mean (range) for quantitative variables.

CRAB, carbapenem-resistant *Acinetobacter baumannii*; EUR, European region; AMR, Region of the Americas; WPR, Western Pacific Region; SEAR, South-East Asia Region; EMR, Eastern Mediterranean Region; AFR, African Region

# **Table S2.** Summary table of risk of bias assessment (n = 249)

| **RoB items and domains** | **n (%)** |
| --- | --- |
| 1.1 Appropriate sampling plan |  |
| Yes | 166 (67%) |
| No | 28 (11%) |
| Unclear | 55 (22%) |
| 1.2 Adequate sample size |  |
| Yes | 7 (3%) |
| No | 242 (97%) |
| 1.3 Adequate description of setting and subjects |  |
| Yes | 145 (58%) |
| No | 104 (42%) |
| 1.4 Sufficient sample coverage |  |
| Yes | 229 (92%) |
| No | 20 (8%) |
| 2.1 Valid method for carbapenamase detection |  |
| Yes | 233 (94%) |
| No | 16 (6%) |
| 2.2 Standardised protocol for carbapenemase detection |  |
| Yes | 247 (99%) |
| No | 2 (1%) |
| Population and setting RoB |  |
| Low | 84 (34%) |
| Moderate-to-high | 165 (66%) |
| Condition measurement RoB |  |
| Low | 231 (93%) |
| Moderate-to-high | 18 (7%) |
| Overall RoB |  |
| Low | 76 (31%) |
| Moderate-to-high | 173 (69%) |

RoB, risk of bias.

# **Table S3.** Critical appraisal for risk of bias of reviewed studies

| **Study** | **Q1** | **Q2** | **Q3** | **Q4** | **Q5** | **Q6** | **PS** | **CM** | **Overall** |
| --- | --- | --- | --- | --- | --- | --- | --- | --- | --- |
| Yek C, 2024 | N | N | Y | N | Y | Y | MH | L | MH |
| Ababneh Q,, 2021 | Y | N | Y | Y | Y | Y | L | L | L |
| Abbasi E, 2023 | U | N | Y | Y | Y | Y | MH | L | MH |
| Abdollahi A, 2021 | Y | N | N | Y | Y | Y | MH | L | MH |
| Abou Fayad AG, 2021 | Y | N | N | Y | Y | Y | MH | L | MH |
| Abouelfetouh A, 2020 | U | N | Y | Y | Y | Y | MH | L | MH |
| Abouelfetouh A,, 2022 | U | N | Y | Y | Y | Y | MH | L | MH |
| Adjei AY, 2021 | U | N | N | Y | Y | Y | MH | L | MH |
| Adler A, 2023 | Y | Y | Y | Y | Y | Y | L | L | L |
| Al-Hashem G, 2021 | N | N | N | Y | Y | Y | MH | L | MH |
| Al-Hassan L, 2021 | Y | N | Y | Y | Y | Y | L | L | L |
| Al-Hassan LL, 2020 | Y | N | N | Y | Y | Y | MH | L | MH |
| Al-Rashed N, 2023 | Y | N | Y | Y | Y | Y | L | L | L |
| Al-Tamimi M, 2022 | Y | N | N | Y | Y | Y | MH | L | MH |
| AlFaris, E.M., 2024 | U | N | Y | Y | Y | Y | MH | L | MH |
| Alcántar-Curiel MD, 2023 | Y | N | N | Y | Y | Y | MH | L | MH |
| Amaral SC, 2020 | U | N | Y | Y | N | Y | MH | MH | MH |
| Anggraini D, 2022 | Y | N | N | Y | Y | Y | MH | L | MH |
| Arif M, 2024 | N | N | Y | Y | Y | Y | MH | L | MH |
| Aung MS, 2021 | Y | N | Y | Y | Y | Y | L | L | L |
| Ayoub Moubareck C, 2021 | Y | N | Y | N | Y | Y | MH | L | MH |
| Azimi L, 2020 | U | N | Y | Y | Y | Y | MH | L | MH |
| Azizi, O, 2021 | Y | N | N | Y | Y | Y | MH | L | MH |
| Bae IK, 2024 | Y | N | N | Y | Y | Y | MH | L | MH |
| Baleivanualala SC, 2024 | Y | N | N | N | Y | Y | MH | L | MH |
| Bandić Pavlović D, 2024 | Y | N | N | Y | Y | Y | MH | L | MH |
| Bandić-Pavlović D, 2020 | N | N | N | Y | Y | Y | MH | L | MH |
| Bansal G, 2020 | U | N | N | Y | Y | Y | MH | L | MH |
| Barcelos Valiatti T, 2022 | Y | N | Y | Y | Y | Y | L | L | L |
| Boulesnam SL, 2023 | Y | N | Y | Y | N | Y | L | MH | MH |
| Bratić V, 2021 | N | N | Y | Y | Y | Y | MH | L | MH |
| Brauncajs M, 2022 | N | N | Y | Y | Y | Y | MH | L | MH |
| Bulens SN, 2024 | N | N | N | N | Y | Y | MH | L | MH |
| Cabral BG, 2021 | Y | N | N | Y | Y | Y | MH | L | MH |
| Camargo CH, 2020 | N | N | Y | Y | Y | Y | MH | L | MH |
| Cano-Martín E, 2021 | Y | N | N | Y | Y | Y | MH | L | MH |
| Carascal MB, 2024 | Y | N | Y | Y | Y | Y | L | L | L |
| Castillo Bejarano JI, 2023 | Y | N | N | Y | Y | Y | MH | L | MH |
| Chandran S, 2023 | Y | N | Y | Y | Y | Y | L | L | L |
| Chatterjee N, 2023 | U | N | Y | Y | Y | Y | MH | L | MH |
| Chen PK, 2024 | U | N | Y | Y | Y | Y | MH | L | MH |
| Chen ZR, 2022 | Y | N | N | Y | Y | Y | MH | L | MH |
| Chen, L, 2021 | Y | N | Y | Y | Y | Y | L | L | L |
| Chitrabanu, N.A., 2021 | U | N | Y | Y | N | Y | MH | MH | MH |
| Chukamnerd A, 2022 | Y | Y | N | Y | Y | Y | L | L | L |
| Cureño-Díaz MA, 2024 | Y | N | Y | Y | Y | Y | L | L | L |
| Dobrović K, 2023 | Y | N | N | Y | Y | Y | MH | L | MH |
| Domingues R, 2024 | U | N | N | Y | Y | Y | MH | L | MH |
| Donadu, M.G., 2021 | U | N | Y | Y | N | Y | MH | MH | MH |
| Dos Santos LA, 2024 | Y | N | Y | Y | Y | Y | L | L | L |
| Ejaz H, 2021 | Y | N | N | Y | Y | Y | MH | L | MH |
| Ejaz H, 2022 | Y | N | N | Y | Y | Y | MH | L | MH |
| Ekinci, B, 2024 | Y | N | N | Y | Y | Y | MH | L | MH |
| El-Hady, H.I.A, 2021 | Y | N | N | Y | Y | Y | MH | L | MH |
| Emami A, 2020 | Y | N | N | Y | Y | Y | MH | L | MH |
| Fam NS, 2020 | Y | N | Y | Y | Y | Y | L | L | L |
| Farajzadeh Sheikh A, 2020 | Y | N | N | Y | Y | Y | MH | L | MH |
| Farzana R, 2022 | Y | N | Y | Y | Y | Y | L | L | L |
| Ferjani S, 2022 | Y | N | N | Y | Y | Y | MH | L | MH |
| Frenk S, 2022 | Y | N | Y | Y | Y | Y | L | L | L |
| Fuentes-González MF, 2024 | Y | N | N | Y | Y | Y | MH | L | MH |
| Galani I, 2023 | Y | N | Y | Y | Y | Y | L | L | L |
| García-Cedrón D, 2023 | Y | N | N | Y | N | Y | MH | MH | MH |
| Garza-González E, 2021 | Y | N | Y | Y | Y | Y | L | L | L |
| Garza-Ramos U, 2023 | U | N | Y | Y | Y | Y | MH | L | MH |
| Garza-Ramos U, 2023 | Y | N | Y | N | Y | Y | MH | L | MH |
| Gautam D, 2023 | Y | N | N | N | Y | Y | MH | L | MH |
| Ghebremedhin B, 2021 | N | N | Y | Y | Y | Y | MH | L | MH |
| Gomez-Gamboa L, 2020 | U | N | N | Y | Y | Y | MH | L | MH |
| Gozalan A, 2021 | Y | N | N | Y | Y | Y | MH | L | MH |
| Grisold AJ, 2021 | Y | N | Y | Y | Y | Y | L | L | L |
| Guclu AU, 2020 | Y | N | Y | Y | N | Y | L | MH | MH |
| Guerra-Sarmiento M, 2021 | U | N | N | Y | Y | Y | MH | L | MH |
| Gurung A, 2024 | U | N | N | Y | Y | Y | MH | L | MH |
| Gür Vural, D, 2024 | Y | N | Y | Y | Y | Y | L | L | L |
| Hadjadj L, 2021 | U | N | Y | Y | Y | Y | MH | L | MH |
| Haji SH, 2022 | Y | N | Y | Y | Y | Y | L | L | L |
| Hamed SM, 2023 | N | N | Y | Y | Y | Y | MH | L | MH |
| Hamza, M.M., 2020 | Y | N | Y | Y | Y | Y | L | L | L |
| Hansen F, 2023 | Y | N | Y | Y | Y | Y | L | L | L |
| Hashemi, B, 2020 | Y | N | N | Y | Y | Y | MH | L | MH |
| Hashemizadeh Z, 2022 | Y | N | N | Y | Y | Y | MH | L | MH |
| Hassan RM, 2021 | Y | N | Y | Y | Y | Y | L | L | L |
| Hegazy EE, 2022 | Y | N | N | Y | Y | Y | MH | L | MH |
| Hernández-Chico I, 2024 | U | N | Y | Y | Y | Y | MH | L | MH |
| Higgins P.G, 2020 | N | N | N | Y | Y | Y | MH | L | MH |
| Higgins PG, 2021 | Y | N | Y | Y | Y | Y | L | L | L |
| Homenta, H, 2022 | Y | N | N | Y | Y | Y | MH | L | MH |
| Hsueh SC, 2024 | Y | Y | Y | Y | Y | Y | L | L | L |
| Hu H, 2024 | Y | N | N | Y | Y | Y | MH | L | MH |
| Hujer AM, 2021 | U | N | Y | Y | Y | Y | MH | L | MH |
| Ijaz, S., 2023 | Y | N | N | Y | Y | Y | MH | L | MH |
| Ingti B, 2020 | Y | N | N | Y | Y | Y | MH | L | MH |
| Jaloot, A.S, 2021 | Y | N | N | Y | N | Y | MH | MH | MH |
| Jian X, 2024 | U | N | Y | N | Y | Y | MH | L | MH |
| Jitmuang A, 2022 | Y | N | N | Y | Y | Y | MH | L | MH |
| Jun, S.H, 2023 | U | N | Y | Y | Y | Y | MH | L | MH |
| Kadivarian S, 2023 | U | N | N | Y | Y | Y | MH | L | MH |
| Kadom, S.M, 2020 | Y | N | Y | Y | Y | Y | L | L | L |
| Kalal, B.S, 2020 | Y | N | Y | Y | Y | Y | L | L | L |
| Karami, F., 2021 | Y | N | N | Y | Y | Y | MH | L | MH |
| Karpova, E, 2022 | U | N | N | Y | Y | Y | MH | L | MH |
| Kazemian H, 2024 | N | N | N | Y | Y | Y | MH | L | MH |
| Khalil MAF, 2021 | N | N | Y | Y | Y | Y | MH | L | MH |
| Khrulnova, S.A., 2020 | Y | N | N | Y | Y | Y | MH | L | MH |
| Khuntayaporn P, 2021 | Y | N | Y | Y | Y | Y | L | L | L |
| Khuntayaporn P, 2021 | Y | N | Y | Y | Y | Y | L | L | L |
| Kiffer CRV, 2023 | Y | Y | Y | Y | Y | Y | L | L | L |
| Kim D, 2022 | Y | N | N | N | Y | Y | MH | L | MH |
| Kim D, 2022 | Y | N | N | Y | Y | Y | MH | L | MH |
| Koirala J, 2020 | U | N | Y | Y | N | Y | MH | MH | MH |
| Kondratiuk V, 2021 | Y | N | Y | Y | Y | Y | L | L | L |
| Kongthai P, 2021 | U | N | Y | Y | Y | Y | MH | L | MH |
| Kostyanev T, 2021 | U | N | Y | Y | Y | Y | MH | L | MH |
| Kovalchuk V, 2024 | Y | N | Y | Y | Y | Y | L | L | L |
| Kozlov, R.S., 2021 | Y | N | Y | Y | Y | Y | L | L | L |
| Kumar, S., 2020 | U | N | Y | Y | Y | Y | MH | L | MH |
| Kumari M, 2021 | Y | N | N | Y | Y | Y | MH | L | MH |
| Kuo SC, 2021 | Y | N | N | Y | Y | Y | MH | L | MH |
| Lavrinenko A, 2021 | U | N | N | Y | Y | Y | MH | L | MH |
| Leal NC, 2020 | Y | N | Y | Y | Y | Y | L | L | L |
| Lee YL, 2023 | Y | N | N | N | Y | Y | MH | L | MH |
| Li Y, 2024 | Y | N | Y | Y | Y | Y | L | L | L |
| Li YY, 2024 | Y | N | N | Y | Y | Y | MH | L | MH |
| Liu B, 2021 | Y | N | N | Y | Y | Y | MH | L | MH |
| Liu PY, 2020 | Y | N | Y | Y | Y | Y | L | L | L |
| Longjam LA, 2023 | Y | N | Y | Y | Y | Y | L | L | L |
| Lukovic B, 2020 | Y | N | N | Y | Y | Y | MH | L | MH |
| Lukovic B, 2024 | N | N | N | Y | Y | Y | MH | L | MH |
| Luo TL, 2024 | Y | N | Y | Y | Y | Y | L | L | L |
| Luo YC, 2022 | U | N | Y | Y | Y | Y | MH | L | MH |
| Mabrouk SS,, 2020 | Y | N | Y | N | Y | Y | MH | L | MH |
| Makharita RR, 2020 | N | N | Y | Y | N | Y | MH | MH | MH |
| Mancini S, 2023 | U | N | Y | Y | Y | Y | MH | L | MH |
| Marusinec R, 2024 | N | N | N | N | Y | Y | MH | L | MH |
| Massik A, 2021 | Y | N | Y | Y | Y | Y | L | L | L |
| Massik A, 2022 | Y | N | Y | Y | Y | Y | L | L | L |
| Massik A, 2023 | U | N | Y | Y | Y | Y | MH | L | MH |
| McKay SL, 2022 | Y | N | Y | N | Y | Y | MH | L | MH |
| Mehrotra T, 2023 | U | N | Y | Y | Y | Y | MH | L | MH |
| Meliani, S, 2020 | Y | N | Y | Y | Y | Y | L | L | L |
| Miniaoui D, 2023 | Y | N | Y | Y | Y | Y | L | L | L |
| Mohamed, H.A, 2022 | Y | N | N | Y | Y | Y | MH | L | MH |
| Mohamed, S.E.R, 2022 | U | N | Y | Y | Y | Y | MH | L | MH |
| Mohan R, 2020 | N | N | Y | Y | Y | Y | MH | L | MH |
| Monnheimer M, 2021 | Y | N | Y | Y | Y | Y | L | L | L |
| Morales L, 2024 | Y | N | Y | Y | Y | Y | L | L | L |
| Mostafa SH, 2022 | Y | N | Y | Y | Y | Y | L | L | L |
| Mostafa SH, 2023 | U | N | Y | N | Y | Y | MH | L | MH |
| Mottaghiyan Z, 2023 | U | N | Y | Y | Y | Y | MH | L | MH |
| Moulana Z, 2020 | Y | N | N | Y | Y | Y | MH | L | MH |
| Moussa SH, 2023 | U | N | Y | N | Y | Y | MH | L | MH |
| Musila L, 2021 | Y | N | Y | Y | Y | Y | L | L | L |
| Müller C, 2023 | N | N | Y | Y | Y | Y | MH | L | MH |
| Namaei MH, 2021 | Y | N | N | Y | Y | Y | MH | L | MH |
| Nayak G, 2022 | Y | N | Y | Y | Y | Y | L | L | L |
| Nelson K, 2020 | Y | N | Y | N | Y | Y | MH | L | MH |
| Ngoi ST, 2021 | Y | N | N | Y | Y | Y | MH | L | MH |
| Nguyen LP, 2021 | Y | N | Y | Y | Y | Y | L | L | L |
| Nikibakhsh M, 2021 | Y | N | Y | Y | Y | Y | L | L | L |
| Nogbou ND, 2021 | U | N | Y | Y | Y | Y | MH | L | MH |
| Numan, S., 2022 | Y | N | Y | Y | N | Y | L | MH | MH |
| Ogbolu DO, 2020 | Y | N | Y | Y | N | Y | L | MH | MH |
| Okdah L, 2022 | Y | N | N | Y | Y | Y | MH | L | MH |
| Osman M, 2020 | Y | N | N | Y | Y | Y | MH | L | MH |
| Ozdede M, 2024 | Y | N | N | Y | Y | Y | MH | L | MH |
| Palavecino E, 2022 | Y | N | Y | Y | Y | Y | L | L | L |
| Pandey R, 2021 | Y | N | Y | Y | N | Y | L | MH | MH |
| Papadopoulou M, 2024 | Y | N | N | Y | Y | Y | MH | L | MH |
| Pascale R, 2022 | N | N | N | Y | Y | Y | MH | L | MH |
| Perdigão Neto LV, 2020 | Y | N | Y | Y | Y | Y | L | L | L |
| Petazzoni G, 2023 | N | N | N | Y | Y | Y | MH | L | MH |
| Pongparit, S, 2021 | Y | N | Y | Y | Y | Y | L | L | L |
| Pragasam AK, 2020 | U | N | Y | Y | Y | Y | MH | L | MH |
| Prayag PS, 2023 | U | N | Y | Y | Y | Y | MH | L | MH |
| Qu J, 2020 | Y | N | Y | Y | Y | Y | L | L | L |
| Quiñones Pérez D, 2022 | U | N | Y | Y | Y | Y | MH | L | MH |
| Rahbarnia L, 2020 | U | N | N | Y | Y | Y | MH | L | MH |
| Rahman A, 2022 | U | N | Y | N | Y | Y | MH | L | MH |
| Rima M, 2022 | N | N | Y | Y | Y | Y | MH | L | MH |
| Rocha IV, 2024 | U | N | N | Y | Y | Y | MH | L | MH |
| Rodriguez CH, 2020 | Y | N | Y | Y | Y | Y | L | L | L |
| Rodríguez-Aguirregabiria M, 2024 | N | N | N | Y | Y | Y | MH | L | MH |
| Rodríguez-Lucas C, 2021 | Y | N | Y | Y | Y | Y | L | L | L |
| Ruekit S, 2022 | Y | N | Y | Y | Y | Y | L | L | L |
| Sabour S, 2021 | N | Y | Y | Y | Y | N | L | MH | MH |
| Sabour S, 2024 | U | Y | Y | Y | Y | N | L | MH | MH |
| Sadeeva, Z.Z, 2023 | Y | N | N | Y | Y | Y | MH | L | MH |
| Saikia S, 2024 | Y | N | N | Y | Y | Y | MH | L | MH |
| Sakuma M, 2024 | U | N | Y | Y | Y | Y | MH | L | MH |
| Saleem M, 2022 | Y | N | N | Y | Y | Y | MH | L | MH |
| Santajit S, 2023 | Y | N | Y | Y | Y | Y | L | L | L |
| Seifert H, 2023 | Y | N | Y | Y | Y | Y | L | L | L |
| Selim MI, 2024 | U | N | Y | Y | Y | Y | MH | L | MH |
| Serwacki PA, 2024 | Y | N | N | N | Y | Y | MH | L | MH |
| Sewunet T, 2022 | Y | N | N | Y | Y | Y | MH | L | MH |
| Sharifipour E, 2020 | Y | N | N | Y | N | Y | MH | MH | MH |
| Sharma M, 2020 | Y | N | Y | Y | Y | Y | L | L | L |
| Sharma S, 2022 | Y | N | N | Y | Y | Y | MH | L | MH |
| Sharma, V, 2024 | Y | N | Y | Y | Y | Y | L | L | L |
| Shi X, 2021 | Y | N | Y | Y | Y | Y | L | L | L |
| Shrestha M, 2021 | U | N | N | Y | N | Y | MH | MH | MH |
| Soria-Segarra C, 2021 | Y | N | N | Y | Y | Y | MH | L | MH |
| Soria-Segarra C, 2024 | Y | N | Y | Y | Y | Y | L | L | L |
| Sotomayor N, 2024 | U | N | Y | Y | Y | Y | MH | L | MH |
| Strateva TV, 2023 | Y | N | N | Y | Y | Y | MH | L | MH |
| Sánchez-Urtaza S, 2023 | Y | N | N | Y | Y | Y | MH | L | MH |
| Słoczyńska A, 2021 | Y | N | Y | Y | N | Y | L | MH | MH |
| Tada T, 2020 | U | N | Y | Y | Y | Y | MH | L | MH |
| Taj, Z, 2023 | Y | N | N | Y | Y | Y | MH | L | MH |
| Takemura M, 2023 | U | N | Y | Y | Y | Y | MH | L | MH |
| Tawfick MM, 2023 | Y | N | N | Y | Y | Y | MH | L | MH |
| Taylor E, 2021 | N | N | Y | Y | Y | Y | MH | L | MH |
| Thet KT, 2020 | Y | N | Y | Y | Y | Y | L | L | L |
| Tickler IA, 2023 | Y | N | Y | Y | Y | Y | L | L | L |
| Tran, L.S, 2024 | Y | N | N | Y | Y | Y | MH | L | MH |
| Uskudar-Guclu A, 2024 | Y | N | Y | Y | Y | Y | L | L | L |
| Vahhabi A, 2021 | U | N | N | Y | Y | Y | MH | L | MH |
| Valcek A, 2022 | Y | N | Y | Y | Y | Y | L | L | L |
| Vijayakumar S, 2020 | Y | N | Y | Y | Y | Y | L | L | L |
| Vijayakumar S, 2022 | U | N | Y | Y | Y | Y | MH | L | MH |
| Vintilă C, 2024 | N | N | Y | Y | Y | Y | MH | L | MH |
| Wang L, 2024 | N | N | N | Y | Y | Y | MH | L | MH |
| Wang M, 2024 | Y | N | N | Y | Y | Y | MH | L | MH |
| Wasfi R, 2021 | Y | N | Y | Y | Y | Y | L | L | L |
| Wen, W.-H., 2021 | Y | N | N | Y | Y | Y | MH | L | MH |
| Wohlfarth E, 2022 | Y | N | Y | Y | Y | Y | L | L | L |
| Woon JJ, 2021 | Y | N | N | N | Y | Y | MH | L | MH |
| Worku S, 2024 | Y | N | N | Y | Y | Y | MH | L | MH |
| Wu Y, 2024 | Y | N | N | Y | Y | Y | MH | L | MH |
| Xiu Y, 2024 | Y | N | N | Y | Y | Y | MH | L | MH |
| Xu A, 2024 | Y | N | N | Y | Y | Y | MH | L | MH |
| Yadav SK, 2020 | Y | N | N | Y | N | Y | MH | MH | MH |
| Yang Q, 2023 | U | N | N | Y | Y | Y | MH | L | MH |
| Yao Y, 2024 | N | N | Y | Y | Y | Y | MH | L | MH |
| Yin L, 2023 | Y | N | N | Y | Y | Y | MH | L | MH |
| Yin L, 2023 | Y | N | Y | N | Y | Y | MH | L | MH |
| Zahedi Bialvaei, A., 2021 | Y | N | Y | Y | Y | Y | L | L | L |
| Zhang S, 2023 | Y | N | Y | Y | Y | Y | L | L | L |
| Zhang Y, 2021 | Y | N | Y | Y | Y | Y | L | L | L |
| Zhang Y, 2021 | Y | N | N | Y | Y | Y | MH | L | MH |
| Zhao Y, 2023 | Y | N | N | Y | Y | Y | MH | L | MH |
| Zhu LJ, 2020 | Y | N | Y | Y | Y | Y | L | L | L |
| Zornic S, 2022 | Y | N | Y | Y | Y | Y | L | L | L |
| Zou X, 2023 | U | N | Y | Y | Y | Y | MH | L | MH |
| de Freitas SB, 2020 | N | N | Y | Y | Y | Y | MH | L | MH |
| von Laer A, 2022 | Y | Y | N | N | Y | Y | MH | L | MH |
| Álvarez-Marín R, 2021 | Y | N | Y | Y | Y | Y | L | L | L |
| Özkul C, 2021 | Y | N | Y | Y | Y | Y | L | L | L |

**PS**, population and setting domain; **CM**, condition measurement domain, **Y/U/N**, yes/unclear/no; **L**, low risk of bias; **MH**, moderate-to-high risk of bias.

Q1: Was an appropriate sampling plan used?

Q2: Was the sample size adequate?

Q3: Were the study subjects and the setting described in detail?

Q4: Was the data analysis conducted with sufficient coverage of the identified sample?

Q5: Were valid methods used for the identification of the condition?

Q6: Was the condition measured in a standard and reliable way for all subjects?

# **Figure S1.** Caterpillar plot of the random-effects meta-analysis of the proportion of MBL production in carbapenem-resistant *A. baumannii* isolates in studies using phenotypic methods for MBL

Black squares and grey horizontal lines are point estimates and 95% confidence intervals of prevalence proportions in individual studies, respectively. The blue diamond's centre is the population-averaged percentage (pooled prevalence estimate). The diamond's length and the respective bluish-grey vertical area indicate the 95% confidence interval (CI) for the pooled prevalence. The blue horizontal line continuing through the diamond indicates the 95% prediction interval (PI) for the expected prevalence in new studies.

# **Figure S2.** Caterpillar plot of the random-effects meta-analysis of the proportion of NDM production in carbapenem-resistant *A. baumannii* isolates in studies that sought for NDM using molecular methods

Black squares and grey horizontal lines are point estimates and 95% confidence intervals of prevalence proportions in individual studies, respectively. The blue diamond's centre is the population-averaged percentage (pooled prevalence estimate). The diamond's length and the respective bluish-grey vertical area indicate the 95% confidence interval (CI) for the pooled prevalence. The blue horizontal line continuing through the diamond indicates the 95% prediction interval (PI) for the expected prevalence in new studies.

# **Figure S3.** Caterpillar plot of the random-effects meta-analysis of the proportion of NDM production in MBL-producing carbapenem-resistant *A. baumannii* isolates

Black squares and grey horizontal lines are point estimates and 95% confidence intervals of prevalence proportions in individual studies, respectively. The blue diamond's centre is the population-averaged percentage (pooled prevalence estimate). The diamond's length and the respective bluish-grey vertical area indicate the 95% confidence interval (CI) for the pooled prevalence. The blue horizontal line continuing through the diamond indicates the 95% prediction interval (PI) for the expected prevalence in new studies.

**Figure S4**. Caterpillar plot of the random-effects meta-analysis of the proportion of NDM-1 production in NDM A. baumannii isolates in studies that sought for the NDM-1 subtype using molecular methods

Black squares and grey horizontal lines are point estimates and 95% confidence intervals of prevalence proportions in individual studies, respectively. The blue diamond's centre is the population-averaged percentage (pooled prevalence estimate). The diamond's length and the respective bluish-grey vertical area indicate the 95% confidence interval (CI) for the pooled prevalence. The blue horizontal line continuing through the diamond indicates the 95% prediction interval (PI) for the expected prevalence in new studies.

# **Table S4.** Subgroup and univariate meta-regression analyses of the associations between study-level characteristics and the prevalence of metallo-β-lactamases in carbapenem-resistant *A. baumannii*

| **Study characteristic** | **Subgroup** | **N** | **MBL / CRAB % (CI)** | **Odds ratio (CI)** | ***P*** | **R^2^** |
| --- | --- | --- | --- | --- | --- | --- |
| Period | 2012-2019 | 132 | 4.2 (1.7 - 6.7) | Ref. | 0.221 | 1.2% |
|  | 2020-2024 | 101 | 7.2 (2.5 - 11.9) | 1.77 (0.71 - 4.43) |  |  |
| WHO region | EMR | 62 | 42.1 (28.7 - 56.8) | 30.81 (12.63 - 75.14) | <0.001 | 37.2% |
|  | EUR | 50 | 0.9 (0.3 - 2.3) | 0.06 (0.02 - 0.18) | <0.001 |  |
|  | SEAR | 37 | 17.9 (9.6 - 30.9) | 4.32 (1.31 - 14.20) | 0.016 |  |
|  | AMR | 31 | 0.4 (0.0 - 4.2) | 0.11 (0.03 - 0.46) | 0.002 |  |
|  | WPR | 36 | 0.4 (0.1 - 3.0) | 0.09 (0.02 - 0.34) | <0.001 |  |
|  | AFR | 7 | 36.1 (12.2 - 69.8) | 10.31 (0.84 - 126.83) | 0.068 |  |
|  | Multiregional | 10 | 1.3 (0.2 - 6.7) | 0.18 (0.02 - 1.57) | 0.122 |  |
| Multi-country | No | 219 | 6.1 (3.3 - 8.8) | Ref. | 0.034 | 4.1% |
|  | Yes | 14 | 0.9 (0.0 - 2.4) | 0.14 (0.02 - 0.86) |  |  |
| Multi-centre | No | 111 | 8.4 (3.3 - 13.6) | Ref. | 0.045 | 3.8% |
|  | Yes | 122 | 3.6 (1.4 - 5.7) | 0.40 (0.16 - 0.98) |  |  |
| Risk of bias | Moderate/high risk | 157 | 6.0 (2.8 - 9.1) | Ref. | 0.409 | 0.2% |
|  | Low risk | 76 | 4.0 (0.8 - 7.2) | 0.66 (0.25 - 1.76) |  |  |

N number of studies; MBL/CRAB, prevalence of metallo-β-lactamase producers in carbapenem-resistant isolates; CI, 95% confidence interval; EUR, European region; AMR, Region of the Americas; WPR, Western Pacific Region; SEAR, South-East Asia Region; EMR, Eastern Mediterranean Region; AFR, African Region.

*^a^* Defined by the year when data collection ended in each study.

*^b^* The odds ratio for each region compares that region to all other regions combined (e.g. EUR vs. elsewhere).

# **Table S5.** Prevalence estimates of the proportion of metallo-β-lactamases in carbapenem-resistant *A. baumannii* in the Eastern Mediterranean region

| **Region and countries** | **S** | **n/N** | **MBL Prevalence** | **95% CI** | **95% PI** | **I^2^** | **tau^2^** |
| --- | --- | --- | --- | --- | --- | --- | --- |
| Eastern Mediterranean region | 62 | 2022/5038 | 42.1% | 28.7% - 56.8% | 0.7% - 98.7% | 98.1% | 5.24 |
| Bahrain | 1 | 47/50 | 94.0% | 83.8% - 97.9% | ne | 0.0% | 0 |
| Egypt | 17 | 245/787 | 37.9% | 25.6% - 52.0% | 5.0% - 87.8% | 92.4% | 1.25 |
| Iran | 18 | 727/1651 | 56.1% | 27.7% - 81.0% | 0.5% - 99.7% | 98.6% | 6.44 |
| Iraq | 3 | 40/54 | 85.2% | 47.7% - 97.3% | 0.0% - 100.0% | 82.2% | 1.43 |
| Jordan | 3 | 306/859 | 36.9% | 10.3% - 74.8% | 0.0% - 100.0% | 99.1% | 2.01 |
| Kuwait | 1 | 35/234 | 15.0% | 11.0% - 20.1% | ne | 0.0% | 0 |
| Lebanon | 2 | 1/73 | 1.4% | 0.2% - 9.1% | ne | 38.5% | <0.01 |
| Libya | 1 | 3/21 | 14.3% | 5.0% - 34.6% | ne | 0.0% | 0 |
| Morocco | 3 | 169/268 | 22.8% | 0.3% - 97.1% | 0.0% - 100.0% | 99.1% | 13.91 |
| Pakistan | 6 | 300/763 | 19.6% | 4.3% - 56.7% | 0.1% - 98.9% | 97.9% | 3.86 |
| Saudi Arabia | 2 | 87/118 | 96.9% | 5.4% - 100.0% | ne | 97.2% | 9.86 |
| Sudan | 2 | 22/72 | 30.6% | 21.0% - 42.1% | ne | 20.9% | <0.01 |
| Tunisia | 2 | 39/56 | 7.3% | 0.0% - 100.0% | ne | 98.0% | 24.27 |
| United Arab Emirates | 1 | 1/32 | 3.1% | 0.6% - 15.7% | ne | 0.0% | 0 |

Population-averaged prevalence proportions and respective confidence and prediction intervals were estimated using a Binomial-Normal mixed-effects logistic regression model when at least two studies were available. For single studies, the confidence intervals were calculated using Wilson's score method. S, number of studies; n/N is the ratio of the number of isolates producing metallo-β-lactamases over the total number of carbapenem-resistant *A. baumannii* isolates; CI, confidence interval; PI, prediction interval; ne, not estimable.

# **Table S6.** Prevalence estimates of the proportion of metallo-β-lactamases in carbapenem-resistant *A. baumannii* in Europe

| **Region and countries** | **S** | **n/N** | **MBL Prevalence** | **95% CI** | **95% PI** | **I^2^** | **tau^2^** |
| --- | --- | --- | --- | --- | --- | --- | --- |
| European region | 50 | 291/6339 | 0.9% | 0.3% - 2.3% | 0.0% - 57.5% | 95.1% | 5.99 |
| Austria | 1 | 0/114 | <0.1% | 0.0% - 3.3% | ne | 0.0% | 0 |
| Belarus | 1 | 0/47 | <0.1% | 0.0% - 7.6% | ne | 0.0% | 0 |
| Belgium | 1 | 1/40 | 2.5% | 0.4% - 12.9% | ne | 0.0% | 0 |
| Bulgaria | 1 | 0/73 | <0.1% | 0.0% - 5.0% | ne | 0.0% | 0 |
| Croatia | 4 | 0/199 | <0.1% | ne | ne | <0.1% | 1.00 |
| Denmark | 1 | 3/141 | 2.1% | 0.7% - 6.1% | ne | 0.0% | 0 |
| Germany* | 6 | 88/1128 | 6.0% | 2.8% - 10.1% | 0.0% - 18.8% | 48.4% | 0.01 |
| Greece* | 2 | 4/310 | 0.9% | 0.0% - 2.5% | ne | <0.1% | <0.01 |
| Israel | 1 | 54/857 | 6.3% | 4.9% - 8.1% | ne | 0.0% | 0 |
| Italy | 2 | 0/132 | <0.1% | ne | ne | <0.1% | 1.00 |
| Kazakhstan | 1 | 0/182 | <0.1% | 0.0% - 2.1% | ne | 0.0% | 0 |
| Poland* | 2 | 3/109 | 2.4% | 0.1% - 6.7% | ne | <0.1% | <0.01 |
| Portugal | 1 | 18/96 | 18.8% | 12.2% - 27.7% | ne | 0.0% | 0 |
| Romania | 1 | 0/24 | <0.1% | 0.0% - 13.8% | ne | 0.0% | 0 |
| Russia | 3 | 6/598 | 1.0% | 0.5% - 2.2% | 0.0% - 65.1% | <0.1% | <0.01 |
| Serbia | 3 | 50/432 | 12.8% | 3.9% - 34.7% | 0.0% - 100.0% | 95.1% | 1.13 |
| Spain | 7 | 31/615 | <0.1% | 0.0% - 2.2% | 0.0% - 100.0% | 98.8% | 156.01 |
| Switzerland | 1 | 10/106 | 9.4% | 5.2% - 16.5% | ne | 0.0% | 0 |
| Türkiye | 6 | 18/599 | 1.2% | 0.2% - 7.1% | 0.0% - 64.1% | 79.5% | 2.37 |
| Ukraine | 2 | 0/52 | <0.1% | ne | ne | <0.1% | 1.00 |
| United Kingdom | 1 | 1/16 | 6.2% | 1.1% - 28.3% | ne | 0.0% | 0 |
| Multi-country | 2 | 4/469 | 0.5% | 0.0% - 7.3% | ne | 81.8% | 1.44 |

Population-averaged prevalence proportions and respective confidence and prediction intervals were estimated using a Binomial-Normal mixed-effects logistic regression model when at least two studies were available. Whenever computational problems were encountered, the pooled prevalence proportions were estimated using a random effects inverse-variance model following the Freeman-Tukey double-arcsine transformation (these cases are indicated with an asterisk). For single studies, the confidence intervals were calculated using Wilson's score method. S, number of studies; n/N is the ratio of the number of isolates producing metallo-β-lactamases over the total number of carbapenem-resistant *A. baumannii* isolates; CI, confidence interval; PI, prediction interval; ne, not estimable.

# **Table S7.** Prevalence estimates of the proportion of metallo-β-lactamases in carbapenem-resistant *A. baumannii* in the South-East Asia region

| **Region and countries** | **S** | **n/N** | **MBL Prevalence** | **95% CI** | **95% PI** | **I^2^** | **tau^2^** |
| --- | --- | --- | --- | --- | --- | --- | --- |
| South-East Asia region | 37 | 1356/4115 | 17.9% | 9.6% - 30.9% | 0.3% - 94.7% | 98.2% | 4.56 |
| Bangladesh | 2 | 3/58 | 2.4% | 0.0% - 77.0% | ne | 88.1% | 6.08 |
| India | 18 | 1096/2520 | 43.8% | 27.8% - 61.1% | 3.0% - 95.2% | 97.9% | 2.20 |
| Indonesia | 2 | 16/183 | 8.1% | 3.9% - 16.0% | ne | 70.0% | 0.11 |
| Myanmar | 2 | 6/57 | 10.5% | 4.8% - 21.5% | ne | <0.1% | <0.01 |
| Nepal | 2 | 38/101 | 37.6% | 28.7% - 47.4% | ne | <0.1% | <0.01 |
| North Korea | 2 | 0/172 | <0.1% | ne | ne | <0.1% | 1.00 |
| Thailand | 9 | 197/1024 | 5.8% | 1.3% - 22.2% | 0.0% - 94.0% | 98.3% | 4.87 |

Population-averaged prevalence proportions and respective confidence and prediction intervals were estimated using a Binomial-Normal mixed-effects logistic regression model when at least two studies were available. For single studies, the confidence intervals were calculated using Wilson's score method.

S, number of studies; n/N is the ratio of the number of isolates producing metallo-β-lactamases over the total number of carbapenem-resistant *A. baumannii* isolates; CI, confidence interval; PI, prediction interval; ne, not estimable.

# **Table S8.** Prevalence estimates of the proportion of metallo-β-lactamases in carbapenem-resistant *A. baumannii* in the Region of the Americas

| **Region and countries** | **S** | **n/N** | **MBL Prevalence** | **95% CI** | **95% PI** | **I^2^** | **tau^2^** |
| --- | --- | --- | --- | --- | --- | --- | --- |
| Region of the Americas | 31 | 326/29120 | 0.4% | 0.0% - 4.2% | 0.0% - 96.1% | 99.7% | 16.61 |
| Brazil | 9 | 94/17414 | 0.2% | 0.0% - 14.7% | 0.0% - 99.4% | 99.6% | 17.51 |
| Colombia | 1 | 3/38 | 7.9% | 2.7% - 20.8% | ne | 0.0% | 0 |
| Cuba | 1 | 22/186 | 11.8% | 7.9% - 17.3% | ne | 0.0% | 0 |
| Ecuador | 2 | 8/74 | 5.1% | 0.3% - 52.5% | ne | 85.2% | 1.74 |
| Mexico | 7 | 113/315 | <0.1% | 0.0% - 3.9% | 0.0% - 100.0% | 98.0% | 197.81 |
| USA | 8 | 34/4821 | 0.3% | 0.0% - 5.5% | 0.0% - 74.0% | 85.1% | 5.45 |
| Venezuela | 1 | 0/119 | <0.1% | 0.0% - 3.1% | ne | 0.0% | 0 |
| Multi-country* | 2 | 52/6153 | 1.1% | 0.1% - 3.2% | ne | 64.4% | <0.01 |

Population-averaged prevalence proportions and respective confidence and prediction intervals were estimated using a Binomial-Normal mixed-effects logistic regression model when at least two studies were available. Whenever computational problems were encountered, the pooled prevalence proportions were estimated using a random effects inverse-variance model following the Freeman-Tukey double-arcsine transformation (these cases are indicated with an asterisk). For single studies, the confidence intervals were calculated using Wilson's score method.

S, number of studies; n/N is the ratio of the number of isolates producing metallo-β-lactamases over the total number of carbapenem-resistant *A. baumannii* isolates; CI, confidence interval; PI, prediction interval; ne, not estimable.

# **Table S9.** Prevalence estimates of the proportion of metallo-β-lactamases in carbapenem-resistant *A. baumannii* in the Western Pacific region

| **Region and countries** | **S** | **n/N** | **MBL Prevalence** | **95% CI** | **95% PI** | **I^2^** | **tau^2^** |
| --- | --- | --- | --- | --- | --- | --- | --- |
| Western Pacific region | 36 | 339/5840 | 0.4% | 0.1% - 3.0% | 0.0% - 93.8% | 98.2% | 15.04 |
| Cambodia | 1 | 0/18 | <0.1% | 0.0% - 17.6% | ne | 0.0% | 0 |
| China | 22 | 259/3851 | 0.8% | 0.1% - 7.3% | 0.0% - 97.4% | 98.7% | 15.06 |
| Fiji | 1 | 56/131 | 42.7% | 34.6% - 51.3% | ne | 0.0% | 0 |
| Malaysia | 3 | 3/156 | <0.1% | 0.0% - 93.9% | 0.0% - 100.0% | 90.0% | 48.70 |
| Philippines | 1 | 2/25 | 8.0% | 2.2% - 25.0% | ne | 0.0% | 0 |
| South Korea | 3 | 0/1128 | <0.1% | ne | ne | <0.1% | 1.00 |
| Taiwan* | 4 | 2/414 | 0.3% | 0.1% - 1.2% | 0.0% - 3.0% | <0.1% | <0.01 |
| Vietnam | 1 | 17/117 | 14.5% | 9.3% - 22.0% | ne | 0.0% | 0 |

Population-averaged prevalence proportions and respective confidence and prediction intervals were estimated using a Binomial-Normal mixed-effects logistic regression model when at least two studies were available. Whenever computational problems were encountered, the pooled prevalence proportions were estimated using a random effects inverse-variance model following the Freeman-Tukey double-arcsine transformation (these cases are indicated with an asterisk). For single studies, the confidence intervals were calculated using Wilson's score method.

S, number of studies; n/N is the ratio of the number of isolates producing metallo-β-lactamases over the total number of carbapenem-resistant *A. baumannii* isolates; CI, confidence interval; PI, prediction interval; ne, not estimable.

# **Table S10.** Prevalence estimates of the proportion of metallo-β-lactamases in carbapenem-resistant *A. baumannii* in the African region

| **Region and countries** | **S** | **n/N** | **MBL Prevalence** | **95% CI** | **95% PI** | **I^2^** | **tau^2^** |
| --- | --- | --- | --- | --- | --- | --- | --- |
| African region | 7 | 105/206 | 36.1% | 12.2% - 69.8% | 0.4% - 98.8% | 94.3% | 3.22 |
| Algeria | 1 | 3/21 | 14.3% | 5.0% - 34.6% | ne | 0.0% | 0 |
| Ethiopia | 2 | 13/46 | 19.6% | 3.4% - 62.8% | ne | 89.6% | 1.41 |
| Ghana | 1 | 20/22 | 90.9% | 72.2% - 97.5% | ne | 0.0% | 0 |
| Kenya | 1 | 7/27 | 25.9% | 13.2% - 44.7% | ne | 0.0% | 0 |
| South Africa | 2 | 62/90 | 45.8% | 4.5% - 93.9% | ne | 97.7% | 4.03 |

Population-averaged prevalence proportions and respective confidence and prediction intervals were estimated using a Binomial-Normal mixed-effects logistic regression model when at least two studies were available. For single studies, the confidence intervals were calculated using Wilson's score method.

S, number of studies; n/N is the ratio of the number of isolates producing metallo-β-lactamases over the total number of carbapenem-resistant *A. baumannii* isolates; CI, confidence interval; PI, prediction interval; ne, not estimable.

# **Table S11.** Prevalence estimates of the proportion of New Delhi metallo-beta-lactamases (NDM) in carbapenem-resistant *A. baumannii* in the Eastern Mediterranean region

| **Region and countries** | **S** | **n/N** | **NDM Prevalence** | **95% CI** | **95% PI** | **I^2^** | **tau^2^** |
| --- | --- | --- | --- | --- | --- | --- | --- |
| Eastern Mediterranean region | 52 | 682/3851 | 8.2% | 4.2% - 15.5% | 0.1% - 92.1% | 96.9% | 5.76 |
| Bahrain | 1 | 3/50 | 6.0% | 2.1% - 16.2% | ne | 0.0% | 0 |
| Egypt | 17 | 173/787 | 24.6% | 15.5% - 36.7% | 2.7% - 79.1% | 90.5% | 1.24 |
| Iran | 12 | 153/1170 | 0.7% | 0.0% - 16.9% | 0.0% - 98.9% | 98.0% | 15.18 |
| Iraq | 2 | 27/39 | 89.7% | 11.9% - 99.8% | ne | 90.6% | 4.12 |
| Jordan | 2 | 136/237 | 42.0% | 6.2% - 88.9% | ne | 99.2% | 2.93 |
| Kuwait | 1 | 0/234 | <0.1% | 0.0% - 1.6% | ne | 0.0% | 0 |
| Lebanon | 2 | 1/73 | 1.4% | 0.2% - 9.1% | ne | 38.5% | <0.01 |
| Libya | 1 | 3/21 | 14.3% | 5.0% - 34.6% | ne | 0.0% | 0 |
| Morocco* | 3 | 9/268 | 2.5% | 0.2% - 6.5% | 0.0% - 83.3% | 54.3% | 0.01 |
| Pakistan | 6 | 156/763 | 9.2% | 2.1% - 31.9% | 0.0% - 95.5% | 97.2% | 3.10 |
| Saudi Arabia | 1 | 10/81 | 12.3% | 6.8% - 21.3% | ne | 0.0% | 0 |
| Sudan | 1 | 10/40 | 25.0% | 14.2% - 40.2% | ne | 0.0% | 0 |
| Tunisia | 2 | 0/56 | <0.1% | ne | ne | <0.1% | 1.00 |
| United Arab Emirates | 1 | 1/32 | 3.1% | 0.6% - 15.7% | ne | 0.0% | 0 |

Population-averaged prevalence proportions and respective confidence and prediction intervals were estimated using a Binomial-Normal mixed-effects logistic regression model when at least two studies were available. Whenever computational problems were encountered, the pooled prevalence proportions were estimated using a random effects inverse-variance model following the Freeman-Tukey double-arcsine transformation (these cases are indicated with an asterisk). For single studies, the confidence intervals were calculated using Wilson's score method.

S, number of studies; n/N is the ratio of the number of isolates producing metallo-β-lactamases over the total number of carbapenem-resistant *A. baumannii* isolates; CI, confidence interval; PI, prediction interval; ne, not estimable.

# **Table S12.** Prevalence estimates of the proportion of New Delhi metallo-beta-lactamases (NDM) in carbapenem-resistant *A. baumannii* in the European region

| **Region and countries** | **S** | **n/N** | **NDM Prevalence** | **95% CI** | **95% PI** | **I^2^** | **tau^2^** |
| --- | --- | --- | --- | --- | --- | --- | --- |
| European region | 50 | 265/6339 | 0.5% | 0.1% - 1.7% | 0.0% - 61.1% | 95.0% | 7.84 |
| Austria | 1 | 0/114 | <0.1% | 0.0% - 3.3% | ne | 0.0% | 0 |
| Belarus | 1 | 0/47 | <0.1% | 0.0% - 7.6% | ne | 0.0% | 0 |
| Belgium | 1 | 1/40 | 2.5% | 0.4% - 12.9% | ne | 0.0% | 0 |
| Bulgaria | 1 | 0/73 | <0.1% | 0.0% - 5.0% | ne | 0.0% | 0 |
| Croatia | 4 | 0/199 | <0.1% | ne | ne | <0.1% | 1.00 |
| Denmark | 1 | 3/141 | 2.1% | 0.7% - 6.1% | ne | 0.0% | 0 |
| Germany* | 6 | 88/1128 | 6.0% | 2.8% - 10.1% | 0.0% - 18.8% | 48.4% | 0.01 |
| Greece* | 2 | 4/310 | 0.9% | 0.0% - 2.5% | ne | <0.1% | <0.01 |
| Israel | 1 | 54/857 | 6.3% | 4.9% - 8.1% | ne | 0.0% | 0 |
| Italy | 2 | 0/132 | <0.1% | ne | ne | <0.1% | 1.00 |
| Kazakhstan | 1 | 0/182 | <0.1% | 0.0% - 2.1% | ne | 0.0% | 0 |
| Poland* | 2 | 2/109 | 1.3% | 0.2% - 5.0% | ne | <0.1% | <0.01 |
| Portugal | 1 | 0/96 | <0.1% | 0.0% - 3.8% | ne | 0.0% | 0 |
| Romania | 1 | 0/24 | <0.1% | 0.0% - 13.8% | ne | 0.0% | 0 |
| Russia | 3 | 6/598 | 1.0% | 0.5% - 2.2% | 0.0% - 65.1% | <0.1% | <0.01 |
| Serbia | 3 | 50/432 | 12.8% | 3.9% - 34.7% | 0.0% - 100.0% | 95.1% | 1.13 |
| Spain | 7 | 30/615 | <0.1% | 0.0% - 2.2% | 0.0% - 100.0% | 98.8% | 152.41 |
| Switzerland | 1 | 10/106 | 9.4% | 5.2% - 16.5% | ne | 0.0% | 0 |
| Türkiye | 6 | 12/599 | 0.2% | 0.0% - 15.1% | 0.0% - 96.8% | 79.6% | 6.76 |
| Ukraine | 2 | 0/52 | <0.1% | ne | ne | <0.1% | 1.00 |
| United Kingdom | 1 | 1/16 | 6.2% | 1.1% - 28.3% | ne | 0.0% | 0 |
| Multi-country | 2 | 4/469 | 0.5% | 0.0% - 7.3% | ne | 81.8% | 1.44 |

Population-averaged prevalence proportions and respective confidence and prediction intervals were estimated using a Binomial-Normal mixed-effects logistic regression model when at least two studies were available. Whenever computational problems were encountered, the pooled prevalence proportions were estimated using a random effects inverse-variance model following the Freeman-Tukey double-arcsine transformation (these cases are indicated with an asterisk). For single studies, the confidence intervals were calculated using Wilson's score method. S, number of studies; n/N is the ratio of the number of isolates producing metallo-β-lactamases over the total number of carbapenem-resistant *A. baumannii* isolates; CI, confidence interval; PI, prediction interval; ne, not estimable.

# **Table S13.** Prevalence estimates of the proportion of New Delhi metallo-beta-lactamases (NDM) in carbapenem-resistant *A. baumannii* in the South-East Asia region

| **Region and countries** | **S** | **n/N** | **NDM Prevalence** | **95% CI** | **95% PI** | **I^2^** | **tau^2^** |
| --- | --- | --- | --- | --- | --- | --- | --- |
| South-East Asia region | 35 | 892/3962 | 12.2% | 7.1% - 20.2% | 0.4% - 82.4% | 96.9% | 2.89 |
| Bangladesh | 2 | 3/58 | 2.4% | 0.0% - 77.0% | ne | 88.1% | 6.08 |
| India | 16 | 776/2367 | 30.3% | 18.6% - 45.4% | 2.5% - 88.0% | 97.2% | 1.63 |
| Indonesia* | 2 | 11/183 | 5.9% | 2.8% - 9.9% | ne | <0.1% | <0.01 |
| Myanmar | 2 | 6/57 | 10.5% | 4.8% - 21.5% | ne | <0.1% | <0.01 |
| Nepal | 2 | 38/101 | 37.6% | 28.7% - 47.4% | ne | <0.1% | <0.01 |
| North Korea | 2 | 0/172 | <0.1% | ne | ne | <0.1% | 1.00 |
| Thailand | 9 | 58/1024 | 4.3% | 2.4% - 7.9% | 0.7% - 23.8% | 77.8% | 0.56 |

Population-averaged prevalence proportions and respective confidence and prediction intervals were estimated using a Binomial-Normal mixed-effects logistic regression model when at least two studies were available. Whenever computational problems were encountered, the pooled prevalence proportions were estimated using a random effects inverse-variance model following the Freeman-Tukey double-arcsine transformation (these cases are indicated with an asterisk). For single studies, the confidence intervals were calculated using Wilson's score method.

S, number of studies; n/N is the ratio of the number of isolates producing metallo-β-lactamases over the total number of carbapenem-resistant *A. baumannii* isolates; CI, confidence interval; PI, prediction interval; ne, not estimable.

# **Table S14.** Prevalence estimates of the proportion of New Delhi metallo-beta-lactamases (NDM) in carbapenem-resistant *A. baumannii* in the Region of the Americas

| **Region and countries** | **S** | **n/N** | **NDM Prevalence** | **95% CI** | **95% PI** | **I^2^** | **tau^2^** |
| --- | --- | --- | --- | --- | --- | --- | --- |
| Region of the Americas | 29 | 181/29048 | 0.5% | 0.2% - 1.4% | 0.0% - 15.0% | 93.8% | 2.67 |
| Brazil* | 8 | 70/17392 | 0.1% | ne | ne | <0.1% | <0.01 |
| Colombia | 1 | 2/38 | 5.3% | 1.5% - 17.3% | ne | 0.0% | 0 |
| Cuba | 1 | 22/186 | 11.8% | 7.9% - 17.3% | ne | 0.0% | 0 |
| Ecuador | 1 | 0/24 | <0.1% | 0.0% - 13.8% | ne | 0.0% | 0 |
| Mexico* | 7 | 1/315 | <0.1% | ne | 0.0% - 1.3% | <0.1% | <0.01 |
| USA | 8 | 34/4821 | 0.3% | 0.0% - 5.5% | 0.0% - 74.0% | 85.1% | 5.45 |
| Venezuela | 1 | 0/119 | <0.1% | 0.0% - 3.1% | ne | 0.0% | 0 |
| Multi-country* | 2 | 52/6153 | 1.1% | 0.1% - 3.2% | ne | 64.4% | <0.01 |

Population-averaged prevalence proportions and respective confidence and prediction intervals were estimated using a Binomial-Normal mixed-effects logistic regression model when at least two studies were available. Whenever computational problems were encountered, the pooled prevalence proportions were estimated using a random effects inverse-variance model following the Freeman-Tukey double-arcsine transformation (these cases are indicated with an asterisk). For single studies, the confidence intervals were calculated using Wilson's score method.

S, number of studies; n/N is the ratio of the number of isolates producing metallo-β-lactamases over the total number of carbapenem-resistant *A. baumannii* isolates; CI, confidence interval; PI, prediction interval; ne, not estimable.

# **Table S15.** Prevalence estimates of the proportion of New Delhi metallo-beta-lactamases (NDM) in carbapenem-resistant *A. baumannii* in the Western Pacific region

| **Region and countries** | **S** | **n/N** | **NDM Prevalence** | **95% CI** | **95% PI** | **I^2^** | **tau^2^** |
| --- | --- | --- | --- | --- | --- | --- | --- |
| Western Pacific region | 35 | 108/5723 | <0.1% | 0.0% - 1.1% | 0.0% - 94.2% | 92.5% | 26.93 |
| Cambodia | 1 | 0/18 | <0.1% | 0.0% - 17.6% | ne | 0.0% | 0 |
| China | 22 | 48/3851 | 0.2% | 0.0% - 2.4% | 0.0% - 66.6% | 84.0% | 9.54 |
| Fiji | 1 | 56/131 | 42.7% | 34.6% - 51.3% | ne | 0.0% | 0 |
| Malaysia | 3 | 3/156 | <0.1% | 0.0% - 93.9% | 0.0% - 100.0% | 90.0% | 48.70 |
| Philippines | 1 | 1/25 | 4.0% | 0.7% - 19.5% | ne | 0.0% | 0 |
| South Korea | 3 | 0/1128 | <0.1% | ne | ne | <0.1% | 1.00 |
| Taiwan | 4 | 0/414 | <0.1% | ne | ne | <0.1% | 1.00 |
| Vietnam | 0 | 0/0 | ne | ne | ne | ne | ne |

Population-averaged prevalence proportions and respective confidence and prediction intervals were estimated using a Binomial-Normal mixed-effects logistic regression model when at least two studies were available. For single studies, the confidence intervals were calculated using Wilson's score method.

S, number of studies; n/N is the ratio of the number of isolates producing metallo-β-lactamases over the total number of carbapenem-resistant *A. baumannii* isolates; CI, confidence interval; PI, prediction interval; ne, not estimable.

# **Table S16.** Prevalence estimates of the proportion of New Delhi metallo-beta-lactamases (NDM) in carbapenem-resistant *A. baumannii* in the African region

| **Region and countries** | **S** | **n/N** | **NDM Prevalence** | **95% CI** | **95% PI** | **I^2^** | **tau^2^** |
| --- | --- | --- | --- | --- | --- | --- | --- |
| African region | 7 | 84/206 | 26.0% | 7.0% - 62.0% | 0.2% - 98.6% | 93.9% | 3.65 |
| Algeria | 1 | 3/21 | 14.3% | 5.0% - 34.6% | ne | 0.0% | 0 |
| Ethiopia | 2 | 13/46 | 19.6% | 3.4% - 62.8% | ne | 89.6% | 1.41 |
| Ghana | 1 | 20/22 | 90.9% | 72.2% - 97.5% | ne | 0.0% | 0 |
| Kenya | 1 | 7/27 | 25.9% | 13.2% - 44.7% | ne | 0.0% | 0 |
| South Africa | 2 | 41/90 | 5.6% | 0.0% - 98.1% | ne | 97.3% | 11.18 |

Population-averaged prevalence proportions and respective confidence and prediction intervals were estimated using a Binomial-Normal mixed-effects logistic regression model when at least two studies were available. For single studies, the confidence intervals were calculated using Wilson's score method.

S, number of studies; n/N is the ratio of the number of isolates producing metallo-β-lactamases over the total number of carbapenem-resistant *A. baumannii* isolates; CI, confidence interval; PI, prediction interval; ne, not estimable.

# **Figure S5.** Doi plot and LFK index to assess publication bias in reporting prevalence proportions of MBL-producing (on the left) and NDM-producing (on the right) isolates in carbapenem-resistant *A. baumannii*

# **Figure S6.** Doi plot and LFK index to assess publication bias in reporting prevalence proportions of MBL-producing carbapenem-resistant *A. baumannii* isolates, stratified by WHO region

AFR, African Region; AMR, Region of the Americas; EMR, Eastern Mediterranean Region; EUR, European region; SEAR, South-East Asia Region; WPR, Western Pacific Region.

# **Figure S7.** Doi plot and LFK index to assess publication bias in reporting prevalence proportions of NDM-producing carbapenem-resistant *A. baumannii* isolates, stratified by WHO region

AFR, African Region; AMR, Region of the Americas; EMR, Eastern Mediterranean Region; EUR, European region; SEAR, South-East Asia Region; WPR, Western Pacific Region.

# **List of studies included in the review**

[1] Gür Vural D, Zeliha S, Tanriverdi̇ Çayci Y, Kemal B, Asuman B, The investigation of beta lactamase in carbapenem-resistant acinetobacter baumannii isolates, Journal of Experimental and Clinical Medicine (Turkey), Ondokuz Mayis Universitesi, Department of Medical Microbiology, Faculty of Medicine, Ondokuz Mayıs University, Samsun, Turkey, pp. 172-6.

[2] Abouelfetouh A, Torky AS, Aboulmagd E. Role of plasmid carrying bla (NDM) in mediating antibiotic resistance among Acinetobacter baumannii clinical isolates from Egypt. 3 Biotech. 2020;10:170. DOI:https://doi.org/10.1007/s13205-020-2157-y

[3] Al-Hassan LL, Al-Madboly LA. Molecular characterisation of an Acinetobacter baumannii outbreak. Infection prevention in practice. 2020;2:100040. DOI:https://doi.org/10.1016/j.infpip.2020.100040

[4] Amaral SC, Pruski BB, de Freitas SB, Allend SO, Ferreira MRA, Moreira C, Jr., et al. Origanum vulgare essential oil: antibacterial activities and synergistic effect with polymyxin B against multidrug-resistant Acinetobacter baumannii. Molecular biology reports. 2020;47:9615-25. DOI:https://doi.org/10.1007/s11033-020-05989-0

[5] Azimi L, Fallah F, Karimi A, Shirdoust M, Azimi T, Sedighi I, et al. Survey of various carbapenem-resistant mechanisms of Acinetobacter baumannii and Pseudomonas aeruginosa isolated from clinical samples in Iran. Iranian journal of basic medical sciences. 2020;23:1396-400. DOI:https://doi.org/10.22038/IJBMS.2020.44853.10463

[6] Bandić-Pavlović D, Zah-Bogović T, Žižek M, Bielen L, Bratić V, Hrabač P, et al. Gram-negative bacteria as causative agents of ventilator-associated pneumonia and their respective resistance mechanisms. Journal of chemotherapy (Florence, Italy). 2020;32:344-58. DOI:https://doi.org/10.1080/1120009X.2020.1793594

[7] Bansal G, Allen-McFarlane R, Eribo B. Antibiotic Susceptibility, Clonality, and Molecular Characterization of Carbapenem-Resistant Clinical Isolates of Acinetobacter baumannii from Washington DC. International journal of microbiology. 2020;2020:2120159. DOI:https://doi.org/10.1155/2020/2120159

[8] Camargo CH, Cunha MPV, de Barcellos TAF, Bueno MS, Bertani AMJ, Dos Santos CA, et al. Genomic and phenotypic characterisation of antimicrobial resistance in carbapenem-resistant Acinetobacter baumannii hyperendemic clones CC1, CC15, CC79 and CC25. International journal of antimicrobial agents. 2020;56:106195. DOI:https://doi.org/10.1016/j.ijantimicag.2020.106195

[9] de Freitas SB, Amaral SC, Ferreira MRA, Roloff BC, Moreira C, Jr., Conceição FR, et al. Molecular Characterization of Carbapenem-Resistant Acinetobacter baumannii Associated with Nosocomial Infection in the Pelotas, RS, Brazil. Current microbiology. 2020;77:2724-34. DOI:https://doi.org/10.1007/s00284-020-02060-w

[10] Emami A, Pirbonyeh N, Keshavarzi A, Bazargani A, Hassanpour S, Javanmardi F. Evaluating the Saliva of Burn ICU Patients for Resistant Infections Harbor Metallo-β-Lactamase Genes. Journal of burn care & research : official publication of the American Burn Association. 2020;41:647-51. DOI:https://doi.org/10.1093/jbcr/iraa007

[11] Fam NS, Gamal D, Mohamed SH, Wasfy RM, Soliman MS, El-Kholy AA, et al. Molecular Characterization of Carbapenem/Colistin-Resistant Acinetobacter baumannii Clinical Isolates from Egypt by Whole-Genome Sequencing. Infection and drug resistance. 2020;13:4487-93. DOI:https://doi.org/10.2147/IDR.S288865

[12] Farajzadeh Sheikh A, Savari M, Abbasi Montazeri E, Khoshnood S. Genotyping and molecular characterization of clinical Acinetobacter baumannii isolates from a single hospital in Southwestern Iran. Pathogens and global health. 2020;114:251-61. DOI:https://doi.org/10.1080/20477724.2020.1765124

[13] Gomez-Gamboa L, Perozo-Mena A, Bermudez-Gonzalez J, Villavicencio C, Villasmil J, Ginestre MM, et al. Detection of carbapenemase-producing bacteria in a public healthcare center from Venezuela. Journal of infection in developing countries. 2020;15:163-7. DOI:https://doi.org/10.3855/jidc.13567

[14] Guclu AU, Gozen AG. Genetic Diversity of OXA-like Genes in Multidrug-Resistant Acinetobacter baumannii Strains from ICUs. Clinical laboratory. 2020;66:DOI:https://doi.org/10.7754/Clin.Lab.2020.200135

[15] Hamza MM, Hadi OM. Detection of qnr A and New Delhi metallo- beta- lactamase-1 (bla NDM-1) in Acinetobacter baumannii isolated from clinical samples in Hillah hospitals. Annals of Tropical Medicine and Public Health. 2020;23:DOI:https://doi.org/10.36295/ASRO.2020.231401

[16] Hashemi B, Afkhami H, Khaledi M, Kiani M, Bialvaei AZ, Fathi J, et al. Frequency of Metalo beta Lactamase genes, bla IMP1, INT 1 in Acinetobacter baumanii isolated from burn patients North of Iran. Gene Reports. 2020;21:DOI:https://doi.org/10.1016/j.genrep.2020.100800

[17] Higgins PG, Hagen RM, Podbielski A, Frickmann H, Warnke P. Molecular epidemiology of carbapenem-resistant acinetobacter baumannii isolated from war-injured patients from the eastern Ukraine. Antibiotics. 2020;9:1-10. DOI:https://doi.org/10.3390/antibiotics9090579

[18] Ingti B, Upadhyay S, Hazarika M, Khyriem AB, Paul D, Bhattacharya P, et al. Distribution of carbapenem resistant Acinetobacter baumannii with bla(ADC-30) and induction of ADC-30 in response to beta-lactam antibiotics. Research in microbiology. 2020;171:128-33. DOI:https://doi.org/10.1016/j.resmic.2020.01.002

[19] Kadom SM, Abid IN. Detection of blaOXA-51-like and blaVIM carbapenemase genes in acinetobacter baumannii isolated from burn patients. International Journal of Pharmaceutical Research. 2020;12:1812-9. DOI:https://doi.org/10.31838/ijpr/2020.12.02.188

[20] Kalal BS, Chandran SP, Yoganand R, Nagaraj S. Molecular characterization of carbapenem-resistant Acinetobacter baumannii strains from a tertiary care center in South India. Infectio. 2020;24:27-34. DOI:https://doi.org/10.22354/in.v24i1.824

[21] Khrulnova SA, Korobova AG, Fedorova AV, Frolova IN, Klyasova GA. Change in the Clonal Structure of Carbapenem not Susceptible Acinetobacter baumannii Isolated from the Blood Culture of Patients with Hematological Malignancies. Molecular Genetics, Microbiology and Virology. 2020;35:145-51. DOI:https://doi.org/10.3103/S0891416820030039

[22] Koirala J, Tyagi I, Guntupalli L, Koirala S, Chapagain U, Quarshie C, et al. OXA-23 and OXA-40 producing carbapenem-resistant Acinetobacter baumannii in Central Illinois. Diagnostic microbiology and infectious disease. 2020;97:114999. DOI:https://doi.org/10.1016/j.diagmicrobio.2020.114999

[23] Kumar S, Singhal L, Ray P, Gautam V. Over-expression of rnd and mate efflux pumps contribute to decreased susceptibility in clinical isolates of carbapenem resistant acinetobacter baumannii. International Journal of Pharmaceutical Research. 2020;12:342-9. DOI:https://doi.org/10.31838/ijpr/2020.12.03.050

[24] Leal NC, Campos TL, Rezende AM, Docena C, Mendes-Marques CL, de Sá Cavalcanti FL, et al. Comparative Genomics of Acinetobacter baumannii Clinical Strains From Brazil Reveals Polyclonal Dissemination and Selective Exchange of Mobile Genetic Elements Associated With Resistance Genes. Frontiers in microbiology. 2020;11:1176. DOI:https://doi.org/10.3389/fmicb.2020.01176

[25] Liu PY, Lee YL, Lu MC, Shao PL, Lu PL, Chen YH, et al. National Surveillance of Antimicrobial Susceptibility of Bacteremic Gram-Negative Bacteria with Emphasis on Community-Acquired Resistant Isolates: Report from the 2019 Surveillance of Multicenter Antimicrobial Resistance in Taiwan (SMART). Antimicrobial agents and chemotherapy. 2020;64:DOI:https://doi.org/10.1128/AAC.01089-20

[26] Lukovic B, Gajic I, Dimkic I, Kekic D, Zornic S, Pozder T, et al. The first nationwide multicenter study of Acinetobacter baumannii recovered in Serbia: emergence of OXA-72, OXA-23 and NDM-1-producing isolates. Antimicrobial resistance and infection control. 2020;9:101. DOI:https://doi.org/10.1186/s13756-020-00769-8

[27] Mabrouk SS, Abdellatif GR, El-Ansary MR, Aboshanab KM, Ragab YM. Carbapenemase Producers Among Extensive Drug-Resistant Gram-Negative Pathogens Recovered from Febrile Neutrophilic Patients in Egypt. Infection and drug resistance. 2020;13:3113-24. DOI:https://doi.org/10.2147/IDR.S269971

[28] Makharita RR, El-Kholy I, Hetta HF, Abdelaziz MH, Hagagy FI, Ahmed AA, et al. Antibiogram and Genetic Characterization of Carbapenem-Resistant Gram-Negative Pathogens Incriminated in Healthcare-Associated Infections. Infection and drug resistance. 2020;13:3991-4002. DOI:https://doi.org/10.2147/IDR.S276975

[29] Meliani S, Toumi S, Djahoudi H, Deghdegh K, Djahoudi KA. Synergistic combination of colistin with imipenem, amikacine or ciprofloxacin against Acinetobacter baumannii and Pseudomonas aeruginosa carbapenem-resistant isolated in Annaba hospital Algeria. Biocell. 2020;44:175-82. DOI:https://doi.org/10.32604/biocell.2020.09097

[30] Moulana Z, Babazadeh A, Eslamdost Z, Shokri M, Ebrahimpour S. Phenotypic and genotypic detection of metallo-beta-lactamases in Carbapenem resistant Acinetobacter baumannii. Caspian journal of internal medicine. 2020;11:171-6. DOI:https://doi.org/10.22088/cjim.11.2.171

[31] Nelson K, Rubio-Aparicio D, Tsivkovski R, Sun D, Totrov M, Dudley M, et al. In Vitro Activity of the Ultra-Broad-Spectrum Beta-Lactamase Inhibitor QPX7728 in Combination with Meropenem against Clinical Isolates of Carbapenem-Resistant Acinetobacter baumannii. Antimicrobial agents and chemotherapy. 2020;64:DOI:https://doi.org/10.1128/AAC.01406-20

[32] Ogbolu DO, Alli OAT, Oluremi AS, Ogunjimi YT, Ojebode DI, Dada V, et al. Contribution of NDM and OXA-type carbapenemases to carbapenem resistance in clinical Acinetobacter baumannii from Nigeria. Infectious diseases (London, England). 2020;52:644-50. DOI:https://doi.org/10.1080/23744235.2020.1775881

[33] Osman M, F BH, Rafei R, Mallat H, Tom JE, Raad EB, et al. Investigation of an XDR-Acinetobacter baumannii ST2 outbreak in an intensive care unit of a Lebanese tertiary care hospital. Future microbiology. 2020;15:1535-42. DOI:https://doi.org/10.2217/fmb-2020-0079

[34] Perdigão Neto LV, Oliveira MS, Orsi TD, Prado G, Martins RCR, Leite GC, et al. Alternative drugs against multiresistant Gram-negative bacteria. Journal of global antimicrobial resistance. 2020;23:33-7. DOI:https://doi.org/10.1016/j.jgar.2020.07.025

[35] Pragasam AK, Jennifer SL, Solaimalai D, Muthuirulandi Sethuvel DP, Rachel T, Elangovan D, et al. Expected plazomicin susceptibility in India based on the prevailing aminoglycoside resistance mechanisms in Gram-negative organisms derived from whole-genome sequencing. Indian journal of medical microbiology. 2020;38:313-8. DOI:https://doi.org/10.4103/ijmm.IJMM_20_384

[36] Qu J, Yu R, Wang Q, Feng C, Lv X. Synergistic Antibacterial Activity of Combined Antimicrobials and the Clinical Outcome of Patients With Carbapenemase-Producing Acinetobacter baumannii Infection. Frontiers in microbiology. 2020;11:541423. DOI:https://doi.org/10.3389/fmicb.2020.541423

[37] Rahbarnia L, Farajnia S, Khaneshi H, Farajnia H, Naghili B, Tanomand A. Detection of blaOXA-23 and blaNDM-1 carbapenemase among clinical isolates of A. baumannii in Tabriz, north-west of Iran. Gene Reports. 2020;18:DOI:https://doi.org/10.1016/j.genrep.2019.100555

[38] Rao M, Rashid FA, Shukor S, Hashim R, Ahmad N. Detection of Antimicrobial Resistance Genes Associated with Carbapenem Resistance from the Whole-Genome Sequence of Acinetobacter baumannii Isolates from Malaysia. The Canadian journal of infectious diseases & medical microbiology = Journal canadien des maladies infectieuses et de la microbiologie medicale. 2020;2020:5021064. DOI:https://doi.org/10.1155/2020/5021064

[39] Rodriguez CH, Brune A, Nastro M, Vay C, Famiglietti A. In vitro synergistic activity of the sulbactam/avibactam combination against extensively drug-resistant Acinetobacter baumannii. Journal of medical microbiology. 2020;69:928-31. DOI:https://doi.org/10.1099/jmm.0.001211

[40] Sharifipour E, Shams S, Esmkhani M, Khodadadi J, Fotouhi-Ardakani R, Koohpaei A, et al. Evaluation of bacterial co-infections of the respiratory tract in COVID-19 patients admitted to ICU. BMC infectious diseases. 2020;20:646. DOI:https://doi.org/10.1186/s12879-020-05374-z

[41] Sharma M, Singhal L, Gautam V, Ray P. Distribution of carbapenemase genes in clinical isolates of Acinetobacter baumannii & a comparison of MALDI-TOF mass spectrometry-based detection of carbapenemase production with other phenotypic methods. The Indian journal of medical research. 2020;151:585-91. DOI:https://doi.org/10.4103/ijmr.IJMR_1383_18

[42] Tada T, Uchida H, Hishinuma T, Watanabe S, Tohya M, Kuwahara-Arai K, et al. Molecular epidemiology of multidrug-resistant Acinetobacter baumannii isolates from hospitals in Myanmar. Journal of global antimicrobial resistance. 2020;22:122-5. DOI:https://doi.org/10.1016/j.jgar.2020.02.011

[43] Thet KT, Lunha K, Srisrattakarn A, Lulitanond A, Tavichakorntrakool R, Kuwatjanakul W, et al. Colistin heteroresistance in carbapenem-resistant Acinetobacter baumannii clinical isolates from a Thai university hospital. World journal of microbiology & biotechnology. 2020;36:102. DOI:https://doi.org/10.1007/s11274-020-02873-8

[44] Vijayakumar S, Anandan S, Ms DP, Kanthan K, Vijayabaskar S, Kapil A, et al. Insertion sequences and sequence types profile of clinical isolates of carbapenem-resistant A. baumannii collected across India over four year period. Journal of infection and public health. 2020;13:1022-8. DOI:https://doi.org/10.1016/j.jiph.2019.11.018

[45] Yadav SK, Bhujel R, Hamal P, Mishra SK, Sharma S, Sherchand JB. Burden of Multidrug-Resistant Acinetobacter baumannii Infection in Hospitalized Patients in a Tertiary Care Hospital of Nepal. Infection and drug resistance. 2020;13:725-32. DOI:https://doi.org/10.2147/IDR.S239514

[46] Zhu LJ, Pan Y, Gao CY, Hou PF. Distribution of Carbapenemases and Efflux Pump in Carbapenem-resistance Acinetobacter baumannii. Annals of clinical and laboratory science. 2020;50:241-6.

[47] Ababneh Q, Aldaken N, Jaradat Z, Al Sbei S, Alawneh D, Al-Zoubi E, et al. Molecular epidemiology of carbapenem-resistant Acinetobacter baumannii isolated from three major hospitals in Jordan. International journal of clinical practice. 2021;75:e14998. DOI:https://doi.org/10.1111/ijcp.14998

[48] Abdollahi A, Aliramezani A, Salehi M, Norouzi Shadehi M, Ghourchian S, Douraghi M. Co-infection of ST2(IP) carbapenem-resistant Acinetobacter baumannii with SARS-CoV-2 in the patients admitted to a Tehran tertiary referral hospital. BMC infectious diseases. 2021;21:927. DOI:https://doi.org/10.1186/s12879-021-06642-2

[49] Abou Fayad AG, Haraoui LP, Sleiman A, Jaafar M, Zorgani A, Matar GM, et al. Diversity of Sequence Types and Impact of Fitness Cost among Carbapenem-Resistant Acinetobacter baumannii Isolates from Tripoli, Libya. Antimicrobial agents and chemotherapy. 2021;65:e0027721. DOI:https://doi.org/10.1128/AAC.00277-21

[50] Adjei AY, Vasaikar SD, Apalata T, Okuthe EG, Songca SP. Phylogenetic analysis of carbapenem-resistant Acinetobacter baumannii isolated from different sources using Multilocus Sequence Typing Scheme. Infection, genetics and evolution : journal of molecular epidemiology and evolutionary genetics in infectious diseases. 2021;96:105132. DOI:https://doi.org/10.1016/j.meegid.2021.105132

[51] Al-Hashem G, Rotimi VO, Albert MJ. Antimicrobial Resistance of Serial Isolates of Acinetobacter baumannii Colonizing the Rectum of Adult Intensive Care Unit Patients in a Teaching Hospital in Kuwait. Microbial drug resistance (Larchmont, N.Y.). 2021;27:64-72. DOI:https://doi.org/10.1089/mdr.2020.0106

[52] Al-Hassan L, Elbadawi H, Osman E, Ali S, Elhag K, Cantillon D, et al. Molecular Epidemiology of Carbapenem-Resistant Acinetobacter baumannii From Khartoum State, Sudan. Frontiers in microbiology. 2021;12:628736. DOI:https://doi.org/10.3389/fmicb.2021.628736

[53] Álvarez-Marín R, López-Cerero L, Guerrero-Sánchez F, Palop-Borras B, Rojo-Martín MD, Ruiz-Sancho A, et al. Do specific antimicrobial stewardship interventions have an impact on carbapenem resistance in Gram-negative bacilli? A multicentre quasi-experimental ecological study: time-trend analysis and characterization of carbapenemases. The Journal of antimicrobial chemotherapy. 2021;76:1928-36. DOI:https://doi.org/10.1093/jac/dkab073

[54] Aung MS, Hlaing MS, San N, Aung MT, Mar TT, Kobayashi N. Clonal diversity of Acinetobacter baumannii clinical isolates in Myanmar: identification of novel ST1407 harbouring blaNDM-1. New microbes and new infections. 2021;40:100847. DOI:https://doi.org/10.1016/j.nmni.2021.100847

[55] Ayoub Moubareck C, Hammoudi Halat D, Nabi A, AlSharhan MA, AlDeesi ZO, Han A, et al. Detection of OXA-23, GES-11 and NDM-1 among carbapenem-resistant Acinetobacter baumannii in Dubai: A preliminary study. Journal of global antimicrobial resistance. 2021;24:27-8. DOI:https://doi.org/10.1016/j.jgar.2020.11.016

[56] Azizi O, Fereshteh S, Nasiri O, Ghorbani M, Barzi SM, Badmasti F. The occurrence and characterization of class i, ii, and iii integrons among carbapenemase-producing clinical strains of acinetobacter baumannii in tehran, iran. Jundishapur Journal of Microbiology. 2021;14:DOI:https://doi.org/10.5812/jjm.117766

[57] Bratić V, Mihaljević S, Verzak Ž, Pleško E, Lukić A, Ćaćić M, et al. Prophylactic application of antibiotics selects extended-spectrum β-lactamase and carbapenemases producing Gram-negative bacteria in the oral cavity. Letters in applied microbiology. 2021;73:206-19. DOI:https://doi.org/10.1111/lam.13493

[58] Cabral BG, Brasiliense DM, Furlaneto IP, Rodrigues YC, Lima KVB. Surgical Site Infection Following Caesarean Section by Acinetobacter Species: A Report from a Hyperendemic Setting in the Brazilian Amazon Region. Microorganisms. 2021;9:DOI:https://doi.org/10.3390/microorganisms9040743

[59] Cano-Martín E, Portillo-Calderón I, Pérez-Palacios P, Navarro-Marí JM, Fernández-Sierra MA, Gutiérrez-Fernández J. A Study in a Regional Hospital of a Mid-Sized Spanish City Indicates a Major Increase in Infection/Colonization by Carbapenem-Resistant Bacteria, Coinciding with the COVID-19 Pandemic. Antibiotics (Basel, Switzerland). 2021;10:DOI:https://doi.org/10.3390/antibiotics10091127

[60] Chen L, Tan P, Zeng J, Yu X, Cai Y, Liao K, et al. Impact of an Intervention to Control Imipenem-Resistant Acinetobacter baumannii and Its Resistance Mechanisms: An 8-Year Survey. Frontiers in Microbiology. 2021;11:DOI:https://doi.org/10.3389/fmicb.2020.610109

[61] Chitrabanu NA, Mallya S. Identification, speciation and antibiogram along with detection of metallo beta-lactamase production in acinetobacter isolated from clinical samples in a tertiary care hospital. Journal of Pure and Applied Microbiology. 2021;15:839-44. DOI:https://doi.org/10.22207/JPAM.15.2.38

[62] Donadu MG, Zanetti S, Nagy ÁL, Barrak I, Gajdács M. Insights on carbapenem-resistant Acinetobacter baumannii: Phenotypic characterization of relevant isolates. Acta Biologica Szegediensis. 2021;65:85-92. DOI:https://doi.org/10.14232/ABS.2021.1.85-92

[63] Ejaz H, Ahmad M, Younas S, Junaid K, Abosalif KOA, Abdalla AE, et al. Molecular Epidemiology of Extensively-Drug Resistant Acinetobacter baumannii Sequence Type 2 Co-Harboring bla (NDM) and bla (OXA) From Clinical Origin. Infection and drug resistance. 2021;14:1931-9. DOI:https://doi.org/10.2147/IDR.S310478

[64] El-Hady HIA, Abdelhadi AA. Expression of metallo-β-lactamase genes in carbapenem resistant Acinetobacter baumannii isolates from intensive care unit patients. Microbes and Infectious Diseases. 2021;2:797-806. DOI:https://doi.org/10.21608/MID.2021.97430.1196

[65] Garza-González E, Bocanegra-Ibarias P, Bobadilla-Del-Valle M, Ponce-de-León-Garduño LA, Esteban-Kenel V, Silva-Sánchez J, et al. Drug resistance phenotypes and genotypes in Mexico in representative gram-negative species: Results from the infivar network. PloS one. 2021;16:e0248614. DOI:https://doi.org/10.1371/journal.pone.0248614

[66] Ghebremedhin B, Ahmad-Nejad P. In-Vitro Efficacy of Cefiderocol in Carbapenem-Non-Susceptible Gram-Negative Bacilli of Different Genotypes in Sub-Region of North Rhine Westphalia, Germany. Pathogens (Basel, Switzerland). 2021;10:DOI:https://doi.org/10.3390/pathogens10101258

[67] Gozalan A, Unaldı O, Guldemir D, Aydogan S, Kuzucu C, Cakirlar FK, et al. Molecular Characterization of Carbapenem-Resistant Acinetobacter baumannii Blood Culture Isolates from Three Hospitals in Turkey. Japanese journal of infectious diseases. 2021;74:200-8. DOI:https://doi.org/10.7883/yoken.JJID.2020.478

[68] Grisold AJ, Luxner J, Bedenić B, Diab-Elschahawi M, Berktold M, Wechsler-Fördös A, et al. Diversity of Oxacillinases and Sequence Types in Carbapenem-Resistant Acinetobacter baumannii from Austria. International journal of environmental research and public health. 2021;18:DOI:https://doi.org/10.3390/ijerph18042171

[69] Guerra-Sarmiento M, Ruíz-Martin-Leyes F, Arzuza-Ortega L, Maestre-Serrano R. [Characterization of multiresistant gram-negative bacilli, isolated in patients hospitalized in health institutions in Barranquilla (Colombia)]. Revista chilena de infectologia : organo oficial de la Sociedad Chilena de Infectologia. 2021;38:189-96. DOI:https://doi.org/10.4067/S0716-10182021000200189

[70] Hadjadj L, Syed MA, Abbasi SA, Rolain JM, Jamil B. Diversity of Carbapenem Resistance Mechanisms in Clinical Gram-Negative Bacteria in Pakistan. Microbial drug resistance (Larchmont, N.Y.). 2021;27:760-7. DOI:https://doi.org/10.1089/mdr.2019.0387

[71] Hassan RM, Salem ST, Hassan SIM, Hegab AS, Elkholy YS. Molecular characterization of carbapenem-resistant Acinetobacter baumannii clinical isolates from Egyptian patients. PloS one. 2021;16:e0251508. DOI:https://doi.org/10.1371/journal.pone.0251508

[72] Higgins PG, Hagen RM, Kreikemeyer B, Warnke P, Podbielski A, Frickmann H, et al. Molecular Epidemiology of Carbapenem-Resistant Acinetobacter baumannii Isolates from Northern Africa and the Middle East. Antibiotics (Basel, Switzerland). 2021;10:DOI:https://doi.org/10.3390/antibiotics10030291

[73] Hujer AM, Hujer KM, Leonard DA, Powers RA, Wallar BJ, Mack AR, et al. A comprehensive and contemporary "snapshot" of β-lactamases in carbapenem resistant Acinetobacter baumannii. Diagnostic microbiology and infectious disease. 2021;99:115242. DOI:https://doi.org/10.1016/j.diagmicrobio.2020.115242

[74] Jaloot AS, Owaid MN. Antibiotic resistance pattern and prevalence of multi-drug and extensive resistant acinetobacter baumannii isolates from clinical specimens after military operations Western Iraq. Gazi Medical Journal. 2021;32:381-8. DOI:https://doi.org/10.12996/GMJ.2021.87

[75] Karami F, Nazari R, Adeli H. Detection of Genes Encoding Metallo-beta-lactamases in Carbapenem Resistant Acinetobacter baumannii. Journal of Kerman University of Medical Sciences. 2021;28:559-67. DOI:https://doi.org/10.22062/jkmu.2021.91827

[76] Khalil MAF, Ahmed FA, Elkhateeb AF, Mahmoud EE, Ahmed MI, Ahmed RI, et al. Virulence Characteristics of Biofilm-Forming Acinetobacter baumannii in Clinical Isolates Using a Galleria mellonella Model. Microorganisms. 2021;9:DOI:https://doi.org/10.3390/microorganisms9112365

[77] Khuntayaporn P, Kanathum P, Houngsaitong J, Montakantikul P, Thirapanmethee K, Chomnawang MT. Predominance of international clone 2 multidrug-resistant Acinetobacter baumannii clinical isolates in Thailand: a nationwide study. Annals of clinical microbiology and antimicrobials. 2021;20:19. DOI:https://doi.org/10.1186/s12941-021-00424-z

[78] Khuntayaporn P, Thirapanmethee K, Kanathum P, Chitsombat K, Chomnawang MT. Comparative study of phenotypic-based detection assays for carbapenemase-producing Acinetobacter baumannii with a proposed algorithm in resource-limited settings. PloS one. 2021;16:e0259686. DOI:https://doi.org/10.1371/journal.pone.0259686

[79] Kim D, Yoon EJ, Hong JS, Choi MH, Kim HS, Kim YR, et al. Major Bloodstream Infection-Causing Bacterial Pathogens and Their Antimicrobial Resistance in South Korea, 2017-2019: Phase I Report From Kor-GLASS. Frontiers in microbiology. 2021;12:799084. DOI:https://doi.org/10.3389/fmicb.2021.799084

[80] Kondratiuk V, Jones BT, Kovalchuk V, Kovalenko I, Ganiuk V, Kondratiuk O, et al. Phenotypic and genotypic characterization of antibiotic resistance in military hospital-associated bacteria from war injuries in the Eastern Ukraine conflict between 2014 and 2020. The Journal of hospital infection. 2021;112:69-76. DOI:https://doi.org/10.1016/j.jhin.2021.03.020

[81] Kongthai P, Thummeepak R, Leungtongkam U, Pooarlai R, Kitti T, Thanwisai A, et al. Insight into Molecular Epidemiology, Antimicrobial Resistance, and Virulence Genes of Extensively Drug-Resistant Acinetobacter baumannii in Thailand. Microbial drug resistance (Larchmont, N.Y.). 2021;27:350-9. DOI:https://doi.org/10.1089/mdr.2020.0064

[82] Kostyanev T, Xavier BB, García-Castillo M, Lammens C, Bravo-Ferrer Acosta J, Rodríguez-Baño J, et al. Phenotypic and molecular characterizations of carbapenem-resistant Acinetobacter baumannii isolates collected within the EURECA study. International journal of antimicrobial agents. 2021;57:106345. DOI:https://doi.org/10.1016/j.ijantimicag.2021.106345

[83] Kozlov RS, Azizov IS, Dekhnich АV, Ivanchik NV, Kuzmenkov АY, Martinovich АА, et al. In vitro activity of biapenem and other carbapenems against Russian clinical isolates of Pseudomonas aeruginosa, Acinetobacter spp., and Enterobacterales. Kliniceskaa Mikrobiologia i Antimikrobnaa Himioterapia. 2021;23:280-91. DOI:https://doi.org/10.36488/cmac.2021.3.280-291

[84] Kumari M, Verma S, Venkatesh V, Gupta P, Tripathi P, Agarwal A, et al. Emergence of blaNDM-1 and blaVIM producing Gram-negative bacilli in ventilator-associated pneumonia at AMR Surveillance Regional Reference Laboratory in India. PloS one. 2021;16:e0256308. DOI:https://doi.org/10.1371/journal.pone.0256308

[85] Kuo SC, Wang YC, Tan MC, Huang WC, Shiau YR, Wang HY, et al. In vitro activity of imipenem/relebactam, meropenem/vaborbactam, ceftazidime/avibactam, cefepime/zidebactam and other novel antibiotics against imipenem-non-susceptible Gram-negative bacilli from Taiwan. The Journal of antimicrobial chemotherapy. 2021;76:2071-8. DOI:https://doi.org/10.1093/jac/dkab141

[86] Lavrinenko A, Sheck E, Kolesnichenko S, Azizov I, Turmukhambetova A. Antibiotic Resistance and Genotypes of Nosocomial Strains of Acinetobacter baumannii in Kazakhstan. Antibiotics (Basel, Switzerland). 2021;10:DOI:https://doi.org/10.3390/antibiotics10040382

[87] Liu B, Liu L. Molecular Epidemiology and Mechanisms of Carbapenem-Resistant Acinetobacter baumannii Isolates from ICU and Respiratory Department Patients of a Chinese University Hospital. Infection and drug resistance. 2021;14:743-55. DOI:https://doi.org/10.2147/IDR.S299540

[88] Massik A, Hibaoui L, Arhoune B, Yahyaoui G, Oumokhtar B, Mahmoud M. Detection of metallo-beta lactamases and oxacillinase genes in carbapenem-resistant Acinetobacter baumannii strains isolated in Morocco. The Pan African medical journal. 2021;40:210. DOI:https://doi.org/10.11604/pamj.2021.40.210.28663

[89] Monnheimer M, Cooper P, Amegbletor HK, Pellio T, Groß U, Pfeifer Y, et al. High Prevalence of Carbapenemase-Producing Acinetobacter baumannii in Wound Infections, Ghana, 2017/2018. Microorganisms. 2021;9:DOI:https://doi.org/10.3390/microorganisms9030537

[90] Musila L, Kyany'a C, Maybank R, Stam J, Oundo V, Sang W. Detection of diverse carbapenem and multidrug resistance genes and high-risk strain types among carbapenem non-susceptible clinical isolates of target gram-negative bacteria in Kenya. PloS one. 2021;16:e0246937. DOI:https://doi.org/10.1371/journal.pone.0246937

[91] Namaei MH, Yousefi M, Askari P, Roshanravan B, Hashemi A, Rezaei Y. High prevalence of multidrug-resistant non-fermentative Gram-negative bacilli harboring bla (IMP-1) and bla (VIM-1) metallo-beta-lactamase genes in Birjand, south-east Iran. Iranian journal of microbiology. 2021;13:470-9. DOI:https://doi.org/10.18502/ijm.v13i4.6971

[92] Ngoi ST, Chong CW, Ponnampalavanar SSS, Tang SN, Idris N, Abdul Jabar K, et al. Genetic mechanisms and correlated risk factors of antimicrobial-resistant ESKAPEE pathogens isolated in a tertiary hospital in Malaysia. Antimicrobial resistance and infection control. 2021;10:70. DOI:https://doi.org/10.1186/s13756-021-00936-5

[93] Nguyen LP, Park CS, Pinto NA, Lee H, Seo HS, Vu TN, et al. In Vitro Activity of a Novel Siderophore-Cephalosporin LCB10-0200 (GT-1), and LCB10-0200/Avibactam, against Carbapenem-Resistant Escherichia coli, Klebsiella pneumoniae, Acinetobacter baumannii, and Pseudomonas aeruginosa Strains at a Tertiary Hospital in Korea. Pharmaceuticals (Basel, Switzerland). 2021;14:DOI:https://doi.org/10.3390/ph14040370

[94] Nikibakhsh M, Firoozeh F, Badmasti F, Kabir K, Zibaei M. Molecular study of metallo-β-lactamases and integrons in Acinetobacter baumannii isolates from burn patients. BMC infectious diseases. 2021;21:782. DOI:https://doi.org/10.1186/s12879-021-06513-w

[95] Nogbou ND, Phofa DT, Nchabeleng M, Musyoki AM. Investigating multi-drug resistant Acinetobacter baumannii isolates at a tertiary hospital in Pretoria, South Africa. Indian journal of medical microbiology. 2021;39:218-23. DOI:https://doi.org/10.1016/j.ijmmb.2021.03.005

[96] Özkul C, Hazırolan G. Oxacillinase Gene Distribution, Antibiotic Resistance, and Their Correlation with Biofilm Formation in Acinetobacter baumannii Bloodstream Isolates. Microbial drug resistance (Larchmont, N.Y.). 2021;27:637-46. DOI:https://doi.org/10.1089/mdr.2020.0130

[97] Pandey R, Mishra SK, Shrestha A. Characterisation of ESKAPE Pathogens with Special Reference to Multidrug Resistance and Biofilm Production in a Nepalese Hospital. Infection and drug resistance. 2021;14:2201-12. DOI:https://doi.org/10.2147/IDR.S306688

[98] Pongparit S, Bunchaleamchai A, Watthanakul N, Boonma N, Massarotti K, Khamuan S, et al. Prevalence of blaoxa genes in carbapenem-resistant acinetobacter baumannii isolates from clinical specimens from nopparatrajathanee hospital. Journal of Current Science and Technology. 2021;11:367-74. DOI:https://doi.org/10.14456/jcst.2021.37

[99] Rodríguez-Lucas C, Rodicio MR, Vázquez X, Escudero D, Quindós B, Alaguero M, et al. Extensively drug-resistant Acinetobacter baumannii carrying bla(OXA-23-like) and armA in a hospital after an intervention in the intensive care unit which ended a long-standing endemicity. European journal of clinical microbiology & infectious diseases : official publication of the European Society of Clinical Microbiology. 2021;40:385-9. DOI:https://doi.org/10.1007/s10096-020-04009-0

[100] Sabour S, Huang JY, Bhatnagar A, Gilbert SE, Karlsson M, Lonsway D, et al. Detection and Characterization of Targeted Carbapenem-Resistant Health Care-Associated Threats: Findings from the Antibiotic Resistance Laboratory Network, 2017 to 2019. Antimicrobial agents and chemotherapy. 2021;65:e0110521. DOI:https://doi.org/10.1128/AAC.01105-21

[101] Shi X, Wang H, Wang X, Jing H, Duan R, Qin S, et al. Molecular characterization and antibiotic resistance of Acinetobacter baumannii in cerebrospinal fluid and blood. PloS one. 2021;16:e0247418. DOI:https://doi.org/10.1371/journal.pone.0247418

[102] Shrestha M, Baral R, Shrestha LB. Metallo-β Lactamase Producing Non-Fermentative Gram-Negative Bacilli from Various Clinical Isolates in a Tertiary Care Hospital: A Descriptive Cross-sectional Study. JNMA; journal of the Nepal Medical Association. 2021;59:875-80. DOI:https://doi.org/10.31729/jnma.6408

[103] Słoczyńska A, Wand ME, Tyski S, Laudy AE. Analysis of bla(CHDL) Genes and Insertion Sequences Related to Carbapenem Resistance in Acinetobacter baumannii Clinical Strains Isolated in Warsaw, Poland. International journal of molecular sciences. 2021;22:DOI:https://doi.org/10.3390/ijms22052486

[104] Soria-Segarra C, Delgado-Valverde M, Serrano-García ML, López-Hernández I, Navarro-Marí JM, Gutiérrez-Fernández J. [Infections in patients colonized with carbapenem-resistant Gram-negative bacteria in a medium Spanish city]. Revista espanola de quimioterapia : publicacion oficial de la Sociedad Espanola de Quimioterapia. 2021;34:450-8. DOI:https://doi.org/10.37201/req/021.2021

[105] Taylor E, Bal AM, Balakrishnan I, Brown NM, Burns P, Clark M, et al. A prospective surveillance study to determine the prevalence of 16S rRNA methyltransferase-producing Gram-negative bacteria in the UK. The Journal of antimicrobial chemotherapy. 2021;76:2428-36. DOI:https://doi.org/10.1093/jac/dkab186

[106] Vahhabi A, Hasani A, Rezaee MA, Baradaran B, Hasani A, Kafil HS, et al. Carbapenem resistance in Acinetobacter baumannii clinical isolates from northwest Iran: high prevalence of OXA genes in sync. Iranian journal of microbiology. 2021;13:282-93. DOI:https://doi.org/10.18502/ijm.v13i3.6388

[107] Wasfi R, Rasslan F, Hassan SS, Ashour HM, Abd El-Rahman OA. Co-Existence of Carbapenemase-Encoding Genes in Acinetobacter baumannii from Cancer Patients. Infectious diseases and therapy. 2021;10:291-305. DOI:https://doi.org/10.1007/s40121-020-00369-4

[108] Wen WH, Wang WR, Li YZ, Chen LJ, Xu LQ. Detection and homology analysis of carbapenemase genes in carbapenem-resistant Acinetobacter baumannii. China Tropical Medicine. 2021;21:849-54. DOI:https://doi.org/10.13604/j.cnki.46-1064/r.2021.09.07

[109] Woon JJ, Teh CSJ, Chong CW, Abdul Jabar K, Ponnampalavanar S, Idris N. Molecular Characterization of Carbapenem-Resistant Acinetobacter baumannii Isolated from the Intensive Care Unit in a Tertiary Teaching Hospital in Malaysia. Antibiotics (Basel, Switzerland). 2021;10:DOI:https://doi.org/10.3390/antibiotics10111340

[110] Zahedi Bialvaei A, Dolatyar Dehkharghani A, Asgari F, Shamloo F, Eslami P, Rahbar M. Modified CIM test as a useful tool to detect carbapenemase activity among extensively drug-resistant Klebsiella pneumoniae, Escherichia coli and Acinetobacter baumannii. Annals of Microbiology. 2021;71:DOI:https://doi.org/10.1186/s13213-021-01634-8

[111] Zhang Y, Ding F, Luo Y, Fan B, Tao Z, Li Y, et al. Distribution pattern of carbapenemases and solitary contribution to resistance in clinical strains of Acinetobacter baumannii. Annals of palliative medicine. 2021;10:9184-91. DOI:https://doi.org/10.21037/apm-21-1805

[112] Zhang Y, Fan B, Luo Y, Tao Z, Nie Y, Wang Y, et al., Comparative analysis of carbapenemases, RND family efflux pumps and biofilm formation potential among Acinetobacter baumannii strains with different carbapenem susceptibility, BMC infectious diseases, England, 2021, p. 841.

[113] Molecular Detection of Carbapenemase Enzymes Directly from Positive Blood Cultures Using Xpert Carba-R, 2022, pp. 365-8.

[114] Abouelfetouh A, Mattock J, Turner D, Li E, Evans BA. Diversity of carbapenem-resistant Acinetobacter baumannii and bacteriophage-mediated spread of the Oxa23 carbapenemase. Microbial genomics. 2022;8:DOI:https://doi.org/10.1099/mgen.0.000752

[115] Al-Tamimi M, Albalawi H, Alkhawaldeh M, Alazzam A, Ramadan H, Altalalwah M, et al. Multidrug-Resistant Acinetobacter baumannii in Jordan. Microorganisms. 2022;10:DOI:https://doi.org/10.3390/microorganisms10050849

[116] Anggraini D, Santosaningsih D, Saharman YR, Endraswari PD, Cahyarini C, Saptawati L, et al. Distribution of Carbapenemase Genes among Carbapenem-Non-Susceptible Acinetobacter baumanii Blood Isolates in Indonesia: A Multicenter Study. Antibiotics (Basel, Switzerland). 2022;11:DOI:https://doi.org/10.3390/antibiotics11030366

[117] Barcelos Valiatti T, Silva Carvalho T, Fernandes Santos F, Silva Nodari C, Cayô R, da Silva JTP, et al. Spread of multidrug-resistant Acinetobacter baumannii isolates belonging to IC1 and IC5 major clones in Rondônia state. Brazilian journal of microbiology : [publication of the Brazilian Society for Microbiology]. 2022;53:795-9. DOI:https://doi.org/10.1007/s42770-022-00706-4

[118] Brauncajs M, Bielec F, Macieja A, Pastuszak-Lewandoska D. Carbapenem-Resistant Gram-Negative Fermenting and Non-Fermenting Rods Isolated from Hospital Patients in Poland-What Are They Susceptible to? Biomedicines. 2022;10:DOI:https://doi.org/10.3390/biomedicines10123049

[119] Chen ZR, Guo HW, Liu J, Pan Q, Fu MZ, Qiu YK, et al. Resistance traits and molecular characterization of multidrug-resistant Acinetobacter baumannii isolates from an intensive care unit of a tertiary hospital in Guangdong, southern China. International microbiology : the official journal of the Spanish Society for Microbiology. 2022;25:471-9. DOI:https://doi.org/10.1007/s10123-022-00233-0

[120] Chukamnerd A, Singkhamanan K, Chongsuvivatwong V, Palittapongarnpim P, Doi Y, Pomwised R, et al. Whole-genome analysis of carbapenem-resistant Acinetobacter baumannii from clinical isolates in Southern Thailand. Computational and structural biotechnology journal. 2022;20:545-58. DOI:https://doi.org/10.1016/j.csbj.2021.12.038

[121] Ejaz H, Qamar MU, Junaid K, Younas S, Taj Z, Bukhari SNA, et al. The Molecular Detection of Class B and Class D Carbapenemases in Clinical Strains of Acinetobacter calcoaceticus-baumannii Complex: The High Burden of Antibiotic Resistance and the Co-Existence of Carbapenemase Genes. Antibiotics (Basel, Switzerland). 2022;11:DOI:https://doi.org/10.3390/antibiotics11091168

[122] Farzana R, Swedberg G, Giske CG, Hasan B. Molecular and genetic characterization of emerging carbapenemase-producing Acinetobacter baumannii strains from patients and hospital environments in Bangladesh. Infection prevention in practice. 2022;4:100215. DOI:https://doi.org/10.1016/j.infpip.2022.100215

[123] Ferjani S, Kanzari L, Maamar E, Hamzaoui Z, Rehaiem A, Ferjani A, et al. Extensively drug-resistant Acinetobacter baumannii co-producing VIM-2 and OXA-23 in intensive care units: Results of a one-day point prevalence in a Tunisian hospital. Infectious diseases now. 2022;52:426-31. DOI:https://doi.org/10.1016/j.idnow.2022.09.003

[124] Frenk S, Temkin E, Lurie-Weinberger MN, Keren-Paz A, Rov R, Rakovitsky N, et al. Large-scale WGS of carbapenem-resistant Acinetobacter baumannii isolates reveals patterns of dissemination of ST clades associated with antibiotic resistance. The Journal of antimicrobial chemotherapy. 2022;77:934-43. DOI:https://doi.org/10.1093/jac/dkac010

[125] Haji SH, Ali FA, Aka STH. Synergistic antibacterial activity of silver nanoparticles biosynthesized by carbapenem-resistant Gram-negative bacilli. Scientific reports. 2022;12:15254. DOI:https://doi.org/10.1038/s41598-022-19698-0

[126] Hashemizadeh Z, Hatam G, Fathi J, Aminazadeh F, Hosseini-Nave H, Hadadi M, et al. The Spread of Insertion Sequences Element and Transposons in Carbapenem Resistant Acinetobacter baumannii in a Hospital Setting in Southwestern Iran. Infection & chemotherapy. 2022;54:275-86. DOI:https://doi.org/10.3947/ic.2022.0022

[127] Hegazy EE, Bahey MG, Abo Hagar AM, Elkholy AA, Mohamed EA. Carbapenem-Resistant Gram-Negative Bacilli Causing Ventilator Associated Pneumonia: Study of MASTDISCS Combi Carba Plus for Detection of Carbapenemase Producing Enterobacterales. Infection and drug resistance. 2022;15:6331-42. DOI:https://doi.org/10.2147/IDR.S385294

[128] Homenta H, Saharman YR, Kuntaman K, Susianti H, Santosaningsih D. Molecular characterization of Clinical carbapenem-resistant Acinetobacter baumannii isolates from two tertiary care hospitals in Indonesia. Research Journal of Pharmacy and Technology. 2022;15:2917-22. DOI:https://doi.org/10.52711/0974-360X.2022.00486

[129] Jitmuang A, Puttinad S, Hemvimol S, Pansasiri S, Horthongkham N. A multiplex pneumonia panel for diagnosis of hospital-acquired and ventilator-associated pneumonia in the era of emerging antimicrobial resistance. Frontiers in cellular and infection microbiology. 2022;12:977320. DOI:https://doi.org/10.3389/fcimb.2022.977320

[130] Karpova E, Tapalski D. The Role of Efflux Pump and Carbapenemase Production in the Development of Multidrug Resistance in Klebsiella Pneumoniae and Acinetobacter Baumannii Strains Isolated from Patients with COVID-19 Infection. Laboratory Diagnostics. Eastern Europe. 2022;11:214-23. DOI:https://doi.org/10.34883/PI.2022.11.2.018

[131] Kim D, Lee H, Choi JS, Croney CM, Park KS, Park HJ, et al. The Changes in Epidemiology of Imipenem-Resistant Acinetobacter baumannii Bacteremia in a Pediatric Intensive Care Unit for 17 Years. Journal of Korean medical science. 2022;37:e196. DOI:https://doi.org/10.3346/jkms.2022.37.e196

[132] Li Y, Guo S, Li X, Yu Y, Yan B, Tian M, et al. Evaluation of the in vitro synergy of polymyxin B-based combinations against polymyxin B -resistant gram-negative bacilli. Microbial pathogenesis. 2022;166:105517. DOI:https://doi.org/10.1016/j.micpath.2022.105517

[133] Luo YC, Hsieh YC, Wu JW, Quyen TLT, Chen YY, Liao WC, et al., Exploring the association between capsular types, sequence types, and carbapenemase genes in Acinetobacter baumannii, International journal of antimicrobial agents, Netherlands, 2022, p. 106470.

[134] Massik A, Hibaoui L, Moussa B, Yahyaoui G, Oumokhtar B, Mahmoud M. First report of SPM metallo-β-lactamases producing Acinetobacter baumannii isolates in Morocco. Iranian journal of microbiology. 2022;14:438-44. DOI:https://doi.org/10.18502/ijm.v14i4.10229

[135] McKay SL, Vlachos N, Daniels JB, Albrecht VS, Stevens VA, Rasheed JK, et al. Molecular Epidemiology of Carbapenem-Resistant Acinetobacter baumannii in the United States, 2013-2017. Microbial drug resistance (Larchmont, N.Y.). 2022;28:645-53. DOI:https://doi.org/10.1089/mdr.2021.0352

[136] Mohamed HA, Gad GFM, Mohamed MF, Ahmed HH, Elfarash AE, Fahmy NF. Genotyping of carbapenem resistant Acinetobacter baumannii isolated from Egyptian patients. Novel Research in Microbiology Journal. 2022;6:1801-20. DOI:https://doi.org/10.21608/nrmj.2022.276319

[137] Mohamed SER, Ahmed Z, Mubarak T, Mohamed S, Higazi H, Ali S. Detection of the blaVIM-2 Gene in Carbapenem-Resistant Acinetobacter baumannii Clinical Isolates in Sudan. International Journal of Biomedicine. 2022;12:636-9. DOI:https://doi.org/10.21103/Article12(4)_OA21

[138] Mostafa SH, Saleh SE, Hamed SM, Aboshanab KM. Febrile illness of bacterial etiology in a public fever hospital in Egypt: High burden of multidrug resistance and WHO priority Gram negative pathogens. Germs. 2022;12:75-85. DOI:https://doi.org/10.18683/germs.2022.1308

[139] Numan S, Al-Saedi F, Burhan I. Prevalence of Extended Spectrum Betalactamase and Metallobetalactamase Producing Acinetobacter baumannii Isolated from Different Infections in Baghdad City of Iraq. International Journal of Drug Delivery Technology. 2022;12:1742-7. DOI:https://doi.org/10.25258/ijddt.12.4.41

[140] Okdah L, AlDosary MS, AlMazyed A, Alkhurayb HM, Almossallam M, Al Obaisi YS, et al. Genomic Characterization of Colistin-Resistant Isolates from the King Fahad Medical City, Kingdom of Saudi Arabia. Antibiotics (Basel, Switzerland). 2022;11:DOI:https://doi.org/10.3390/antibiotics11111597

[141] Palavecino E, Greene SR, Kilic A. Characterisation of carbapenemase genes and antibiotic resistance in carbapenem-resistant Acinetobacter baumannii between 2019 and 2022. Infectious diseases (London, England). 2022;54:951-3. DOI:https://doi.org/10.1080/23744235.2022.2113137

[142] Pascale R, Bussini L, Gaibani P, Bovo F, Fornaro G, Lombardo D, et al. Carbapenem-resistant bacteria in an intensive care unit during the coronavirus disease 2019 (COVID-19) pandemic: A multicenter before-and-after cross-sectional study. Infection control and hospital epidemiology. 2022;43:461-6. DOI:https://doi.org/10.1017/ice.2021.144

[143] Quiñones Pérez D, Aung MS, Carmona Cartaya Y, González Molina MK, Pereda Novales N, Kobayashi N. Clonal diversity of Acinetobacter clinical isolates producing NDM-type carbapenemase in Cuba, 2013-19. IJID regions. 2022;5:93-6. DOI:https://doi.org/10.1016/j.ijregi.2022.08.008

[144] Rahman A, Styczynski A, Khaleque A, Hossain SA, Sadique A, Hossain A, et al. Genomic landscape of prominent XDR Acinetobacter clonal complexes from Dhaka, Bangladesh. BMC genomics. 2022;23:802. DOI:https://doi.org/10.1186/s12864-022-08991-x

[145] Rima M, Oueslati S, Dabos L, Daaboul D, Mallat H, Bou Raad E, et al. Prevalence and Molecular Mechanisms of Carbapenem Resistance among Gram-Negative Bacilli in Three Hospitals of Northern Lebanon. Antibiotics (Basel, Switzerland). 2022;11:DOI:https://doi.org/10.3390/antibiotics11101295

[146] Ruekit S, Srijan A, Serichantalergs O, Margulieux KR, Mc Gann P, Mills EG, et al. Molecular characterization of multidrug-resistant ESKAPEE pathogens from clinical samples in Chonburi, Thailand (2017-2018). BMC infectious diseases. 2022;22:695. DOI:https://doi.org/10.1186/s12879-022-07678-8

[147] Saleem M, Syed Khaja AS, Hossain A, Alenazi F, Said KB, Moursi SA, et al. Molecular Characterization and Antibiogram of Acinetobacter baumannii Clinical Isolates Recovered from the Patients with Ventilator-Associated Pneumonia. Healthcare (Basel, Switzerland). 2022;10:DOI:https://doi.org/10.3390/healthcare10112210

[148] Sewunet T, Asrat D, Woldeamanuel Y, Aseffa A, Giske CG. Molecular epidemiology and antimicrobial susceptibility of Pseudomonas spp. and Acinetobacter spp. from clinical samples at Jimma medical center, Ethiopia. Frontiers in microbiology. 2022;13:951857. DOI:https://doi.org/10.3389/fmicb.2022.951857

[149] Sharma S, Banerjee T, Yadav G, Kumar A. Susceptibility profile of bla (OXA-23) and metallo-β-lactamases co-harbouring isolates of carbapenem resistant Acinetobacter baumannii (CRAB) against standard drugs and combinations. Frontiers in cellular and infection microbiology. 2022;12:1068840. DOI:https://doi.org/10.3389/fcimb.2022.1068840

[150] Valcek A, Nesporova K, Whiteway C, De Pooter T, De Coster W, Strazisar M, et al. Genomic Analysis of a Strain Collection Containing Multidrug-, Extensively Drug-, Pandrug-, and Carbapenem-Resistant Modern Clinical Isolates of Acinetobacter baumannii. Antimicrobial agents and chemotherapy. 2022;66:e0089222. DOI:https://doi.org/10.1128/aac.00892-22

[151] Vijayakumar S, Jacob JJ, Vasudevan K, Mathur P, Ray P, Neeravi A, et al. Genomic Characterization of Mobile Genetic Elements Associated With Carbapenem Resistance of Acinetobacter baumannii From India. Frontiers in microbiology. 2022;13:869653. DOI:https://doi.org/10.3389/fmicb.2022.869653

[152] von Laer A, Eckmanns T, Zacher B, Pfennigwerth N, Gatermann SG, Reichert F, et al. Geographical differences of carbapenem non-susceptible Enterobacterales and Acinetobacter spp. in Germany from 2017 to 2019. Antimicrobial resistance and infection control. 2022;11:25. DOI:https://doi.org/10.1186/s13756-021-01045-z

[153] Wohlfarth E, Kresken M, Higgins PG, Stefanik D, Wille J, Hafner D, et al. The evolution of carbapenem resistance determinants and major epidemiological lineages among carbapenem-resistant Acinetobacter baumannii isolates in Germany, 2010-2019. International journal of antimicrobial agents. 2022;60:106689. DOI:https://doi.org/10.1016/j.ijantimicag.2022.106689

[154] Zornic S, Lukovic B, Petrovic I, Jencic A. Prevalence of multidrug-resistant Gram-negative bacteria from blood cultures and rapid detection of beta-lactamase-encoding genes by multiplex PCR assay. Germs. 2022;12:434-43. DOI:https://doi.org/10.18683/germs.2022.1349

[155] Abbasi E, van Belkum A, Ghaznavi-Rad E. High frequency of carbapenemase in extensively drug-resistant Acinetobacter baumannii isolates in central Iran. World journal of microbiology & biotechnology. 2023;39:321. DOI:https://doi.org/10.1007/s11274-023-03778-y

[156] Adler A, Ghosh H, Gross A, Rechavi A, Lasnoy M, Assous MV, et al. Clinical and molecular features of NDM-producing Acinetobacter baumannii in a multicenter study in Israel. Annals of clinical microbiology and antimicrobials. 2023;22:52. DOI:https://doi.org/10.1186/s12941-023-00607-w

[157] Al-Rashed N, Bindayna KM, Shahid M, Saeed NK, Darwish A, Joji RM, et al. Prevalence of Carbapenemases in Carbapenem-Resistant Acinetobacter baumannii Isolates from the Kingdom of Bahrain. Antibiotics (Basel, Switzerland). 2023;12:DOI:https://doi.org/10.3390/antibiotics12071198

[158] Alcántar-Curiel MD, Huerta-Cedeño M, Jarillo-Quijada MD, Gayosso-Vázquez C, Fernández-Vázquez JL, Hernández-Medel ML, et al. Gram-negative ESKAPE bacteria bloodstream infections in patients during the COVID-19 pandemic. PeerJ. 2023;11:e15007. DOI:https://doi.org/10.7717/peerj.15007

[159] Boulesnam SL, Hamaidi-Chergui F, Benamara M, Azrou S. Phenotypical Comparison between Environmental and Clinical Acinetobacter baumannii Strains Isolated from an Intensive Care Unit. The Malaysian journal of medical sciences : MJMS. 2023;30:85-93. DOI:https://doi.org/10.21315/mjms2023.30.4.8

[160] Castillo Bejarano JI, Llaca Díaz J, ME ElOC, Sánchez Alanís H, Mascareñas de Los Santos AH, Espinosa-Villaseñor F, et al. Carbapenem-Resistant Acinetobacter baumannii Infection in Children From a Third-Level Hospital in Mexico: Clinical Characteristics and Molecular Epidemiology. Journal of the Pediatric Infectious Diseases Society. 2023;12:431-5. DOI:https://doi.org/10.1093/jpids/piad046

[161] Chandran S, Manokaran Y, Vijayakumar S, Shankar BA, Bakthavatchalam YD, Dwarakanathan HT, et al. Enhanced bacterial killing with a combination of sulbactam/minocycline against dual carbapenemase-producing Acinetobacter baumannii. European journal of clinical microbiology & infectious diseases : official publication of the European Society of Clinical Microbiology. 2023;42:645-51. DOI:https://doi.org/10.1007/s10096-023-04583-z

[162] Chatterjee N, Nirwan PK, Srivastava S, Rati R, Sharma L, Sharma P, et al. Trends in carbapenem resistance in Pre-COVID and COVID times in a tertiary care hospital in North India. Annals of clinical microbiology and antimicrobials. 2023;22:1. DOI:https://doi.org/10.1186/s12941-022-00549-9

[163] Dobrović K, Škrobo T, Selec K, Jelić M, Čivljak R, Peršec J, et al. Healthcare-Associated Bloodstream Infections Due to Multidrug-Resistant Acinetobacter baumannii in COVID-19 Intensive Care Unit: A Single-Center Retrospective Study. Microorganisms. 2023;11:DOI:https://doi.org/10.3390/microorganisms11030774

[164] Galani I, Papoutsaki V, Karaiskos I, Moustakas N, Galani L, Maraki S, et al. In vitro activities of omadacycline, eravacycline, cefiderocol, apramycin, and comparator antibiotics against Acinetobacter baumannii causing bloodstream infections in Greece, 2020-2021: a multicenter study. European journal of clinical microbiology & infectious diseases : official publication of the European Society of Clinical Microbiology. 2023;42:843-52. DOI:https://doi.org/10.1007/s10096-023-04616-7

[165] García-Cedrón D, De La Cruz Noriega M, Cabanillas-Chirinos L, Otiniano NM, Rojas-Villacorta W, Salvatierra-Espinola W, et al. Carbapenemase-Producing Bacteria Isolated from ICU Patients of a Peruvian Government Hospital during the COVID-19 Pandemic: A Descriptive Analysis. Medicina (Kaunas, Lithuania). 2023;59:DOI:https://doi.org/10.3390/medicina59101763

[166] Garza-Ramos U, Rodríguez-Medina N, Córdova-Fletes C, Rubio-Mendoza D, Alonso-Hernández CJ, López-Jácome LE, et al. Whole genome analysis of Gram-negative bacteria using the EPISEQ CS application and other bioinformatic platforms. Journal of global antimicrobial resistance. 2023;33:61-71. DOI:https://doi.org/10.1016/j.jgar.2023.02.026

[167] Garza-Ramos U, Silva-Sánchez J, López-Jácome LE, Hernández-Durán M, Colín-Castro CA, Sánchez-Pérez A, et al. Carbapenemase-Encoding Genes and Colistin Resistance in Gram-Negative Bacteria During the COVID-19 Pandemic in Mexico: Results from the Invifar Network. Microbial drug resistance (Larchmont, N.Y.). 2023;29:239-48. DOI:https://doi.org/10.1089/mdr.2022.0226

[168] Gautam D, Dolma KG, Khandelwal B, Goyal RK, Mitsuwan W, Pereira MLG, et al. Acinetobacter baumannii in suspected bacterial infections: Association between multidrug resistance, virulence genes, & biofilm production. The Indian journal of medical research. 2023;158:439-46. DOI:https://doi.org/10.4103/ijmr.ijmr_3470_21

[169] Hamed SM, Elkhatib WF, Brangsch H, Gesraha AS, Moustafa S, Khater DF, et al. Acinetobacter baumannii Global Clone-Specific Resistomes Explored in Clinical Isolates Recovered from Egypt. Antibiotics (Basel, Switzerland). 2023;12:DOI:https://doi.org/10.3390/antibiotics12071149

[170] Hansen F, Porsbo LJ, Frandsen TH, Kaygisiz ANS, Roer L, Henius AE, et al. Characterisation of carbapenemase-producing Acinetobacter baumannii isolates from danish patients 2014-2021: detection of a new international clone - IC11. International journal of antimicrobial agents. 2023;62:106866. DOI:https://doi.org/10.1016/j.ijantimicag.2023.106866

[171] Ijaz S, Ansari F, Nawaz M, Anjum AA, Rasool K. Genetic Characterization of Carbapenem Resistant Acinetobacter baumannii in Tertiary care settings of Lahore, Pakistan. Advancements in Life Sciences. 2023;10:479-85.

[172] Jun SH, Lee DE, Hwang HR, Kim N, Kwon KT, Kim YK, et al. Clonal evolution and antimicrobial resistance of Acinetobacter baumannii isolates from Korean hospitals over the last decade. Infection, Genetics and Evolution. 2023;108:DOI:https://doi.org/10.1016/j.meegid.2023.105404

[173] Kadivarian S, Rostamian M, Dashtbin S, Kooti S, Zangeneh Z, Abiri R, et al. High burden of MDR, XDR, PDR, and MBL producing Gram negative bacteria causing infections in Kermanshah health centers during 2019-2020. Iranian journal of microbiology. 2023;15:359-72. DOI:https://doi.org/10.18502/ijm.v15i3.12896

[174] Kiffer CRV, Rezende TFT, Costa-Nobre DT, Marinonio ASS, Shiguenaga LH, Kulek DNO, et al. A 7-Year Brazilian National Perspective on Plasmid-Mediated Carbapenem Resistance in Enterobacterales, Pseudomonas aeruginosa, and Acinetobacter baumannii Complex and the Impact of the Coronavirus Disease 2019 Pandemic on Their Occurrence. Clinical infectious diseases : an official publication of the Infectious Diseases Society of America. 2023;77:S29-S37. DOI:https://doi.org/10.1093/cid/ciad260

[175] Lee YL, Ko WC, Hsueh PR. Geographic patterns of Acinetobacter baumannii and carbapenem resistance in the Asia-Pacific Region: results from the Antimicrobial Testing Leadership and Surveillance (ATLAS) program, 2012-2019. International journal of infectious diseases : IJID : official publication of the International Society for Infectious Diseases. 2023;127:48-55. DOI:https://doi.org/10.1016/j.ijid.2022.12.010

[176] Longjam LA, Tsering DC, Das D. Molecular Characterization of Class A-ESBLs, Class B-MBLs, Class C-AmpC, and Class D-OXA Carbapenemases in MDR Acinetobacter baumannii Clinical Isolates in a Tertiary Care Hospital, West Bengal, India. Cureus. 2023;15:e43656. DOI:https://doi.org/10.7759/cureus.43656

[177] Mancini S, Seth-Smith HMB, Kolesnik-Goldmann N, Hinic V, Roloff T, Imkamp F, et al. Evaluation of the RESIST ACINETO multiplex immunochromatographic assay for detection of OXA-23-like, OXA-40/58-like and NDM carbapenemase production in Acinetobacter baumannii. The Journal of antimicrobial chemotherapy. 2023;78:2771-4. DOI:https://doi.org/10.1093/jac/dkad253

[178] Massik A, Hibaoui L, Benboubker M, Yahyaoui G, Oumokhtar B, Mahmoud M. Acinetobacter baumannii Carbapenemase Producers in Morocco: Genetic Diversity. Cureus. 2023;15:e43629. DOI:https://doi.org/10.7759/cureus.43629

[179] Mehrotra T, Konar D, Pragasam AK, Kumar S, Jana P, Babele P, et al. Antimicrobial resistance heterogeneity among multidrug-resistant Gram-negative pathogens: Phenotypic, genotypic, and proteomic analysis. Proceedings of the National Academy of Sciences of the United States of America. 2023;120:e2305465120. DOI:https://doi.org/10.1073/pnas.2305465120

[180] Miniaoui D, Dziri O, Ben Lamine Y, El Salabi AA, Omar EO, Slimene K, et al. Prevalence of carbapenemases among Gram-negative bacteria in Tunisia: first report of KPC-2 producing Acinetobacter baumannii. Journal of infection in developing countries. 2023;17:1591-7. DOI:https://doi.org/10.3855/jidc.17978

[181] Mostafa SH, Saleh SE, Khaleel EF, Badi RM, Aboshanab KM, Hamed SM. Phenotypic and Genotypic Analysis of Bacterial Pathogens Recovered from Patients Diagnosed with Fever of Unknown Origin in Egypt. Antibiotics (Basel, Switzerland). 2023;12:DOI:https://doi.org/10.3390/antibiotics12081294

[182] Mottaghiyan Z, Esmaeili D, Ahmadi MH, Niakan M. Designing a Multiplex PCR for Rapid and Accurate Detection of Metallobetalactamases Resistant Genes from Acinetobacter baumaniiIsolates in Tehran City, Iran. Iranian journal of pathology. 2023;18:449-55. DOI:https://doi.org/10.30699/IJP.2023.2007142.3144

[183] Moussa SH, Shapiro AB, McLeod SM, Iyer R, Carter NM, Tsai YK, et al. Molecular drivers of resistance to sulbactam-durlobactam in contemporary clinical isolates of Acinetobacter baumannii. Antimicrobial agents and chemotherapy. 2023;67:e0066523. DOI:https://doi.org/10.1128/aac.00665-23

[184] Müller C, Reuter S, Wille J, Xanthopoulou K, Stefanik D, Grundmann H, et al. A global view on carbapenem-resistant Acinetobacter baumannii. mBio. 2023;14:e0226023. DOI:https://doi.org/10.1128/mbio.02260-23

[185] Petazzoni G, Bellinzona G, Merla C, Corbella M, Monzillo V, Samuelsen Ø, et al. The COVID-19 Pandemic Sparked Off a Large-Scale Outbreak of Carbapenem-Resistant Acinetobacter baumannii from the Endemic Strains at an Italian Hospital. Microbiology spectrum. 2023;11:e0450522. DOI:https://doi.org/10.1128/spectrum.04505-22

[186] Prayag PS, Patwardhan SA, Joshi RS, Panchakshari SP, Rane T, Prayag AP. Enzyme Patterns and Factors Associated with Mortality among Patients with Carbapenem Resistant AcinetobacterBaumannii (CRAB) Bacteremia: Real World Evidence from a Tertiary Center in India. Indian journal of critical care medicine : peer-reviewed, official publication of Indian Society of Critical Care Medicine. 2023;27:663-8. DOI:https://doi.org/10.5005/jp-journals-10071-24534

[187] Sadeeva ZZ, Novikova IE, Alyabyeva NM, Lazareva AV, Komyagina TM, Karaseva OV, et al. ACINETOBACTER BAUMANNII IN BLOOD-BORNE AND CENTRAL NERVOUS SYSTEM INFECTIONS IN INTENSIVE CARE UNIT CHILDREN: MOLECULAR AND GENETIC CHARACTERISTICS AND CLINICAL SIGNIFICANCE. Russian Journal of Infection and Immunity. 2023;13:289-301. DOI:https://doi.org/10.15789/2220-7619-ABI-2091

[188] Sánchez-Urtaza S, Ocampo-Sosa A, Molins-Bengoetxea A, El-Kholy MA, Hernandez M, Abad D, et al. Molecular characterization of multidrug resistant Acinetobacter baumannii clinical isolates from Alexandria, Egypt. Frontiers in cellular and infection microbiology. 2023;13:1208046. DOI:https://doi.org/10.3389/fcimb.2023.1208046

[189] Santajit S, Bhoopong P, Kong-Ngoen T, Tunyong W, Horpet D, Paehoh-Ele W, et al. Phenotypic and Genotypic Investigation of Carbapenem-Resistant Acinetobacter baumannii in Maharaj Nakhon Si Thammarat Hospital, Thailand. Antibiotics (Basel, Switzerland). 2023;12:DOI:https://doi.org/10.3390/antibiotics12030580

[190] Seifert H, Müller C, Stefanik D, Higgins PG, Wohlfarth E, Kresken M. In Vitro Activity of Cefiderocol against a Global Collection of Carbapenem-Resistant Acinetobacter baumannii Isolates. Antibiotics (Basel, Switzerland). 2023;12:DOI:https://doi.org/10.3390/antibiotics12071172

[191] Strateva TV, Sirakov I, Stoeva TJ, Stratev A, Peykov S. Phenotypic and Molecular Characteristics of Carbapenem-Resistant Acinetobacter baumannii Isolates from Bulgarian Intensive Care Unit Patients. Microorganisms. 2023;11:DOI:https://doi.org/10.3390/microorganisms11040875

[192] Taj Z, Rasool MH, Khurshid M, Aslam B, Qamar MU. Insights into the Intersection of Biocide Resistance, Efflux Pumps, and Sequence Types in Carbapenem-Resistant Acinetobacter baumannii: A Multicenter Study. Pathogens. 2023;12:DOI:https://doi.org/10.3390/pathogens12070899

[193] Takemura M, Wise MG, Hackel MA, Sahm DF, Yamano Y. In vitro activity of cefiderocol against MBL-producing Gram-negative bacteria collected in North America and Europe in five consecutive annual multinational SIDERO-WT surveillance studies (2014-2019). The Journal of antimicrobial chemotherapy. 2023;78:2019-27. DOI:https://doi.org/10.1093/jac/dkad200

[194] Tawfick MM, Adulall AK, El-Kholy AA, Manakhly ARE. Mutation-based fluoroquinolone resistance in carbapenem-resistant Acinetobacter baumannii and Escherichia coli isolates causing catheter-related bloodstream infections. International journal of health sciences. 2023;17:18-25.

[195] Tickler IA, Kawa D, Obradovich AE, Fang FC, Tenover FC, The Healthcare Associated Infections C. Characterization of Carbapenemase- and ESBL-Producing Gram-Negative Bacilli Isolated from Patients with Urinary Tract and Bloodstream Infections. Antibiotics (Basel, Switzerland). 2023;12:DOI:https://doi.org/10.3390/antibiotics12091386

[196] Yang Q, Kamat S, Mohamed N, Valdez RR, Lin S, Su M, et al. Antimicrobial Susceptibility Among Gram-Negative Isolates in Pediatric Patients in Latin America, Africa-Middle East, and Asia From 2016-2020 Compared to 2011-2015: Results From the ATLAS Surveillance Study. Journal of the Pediatric Infectious Diseases Society. 2023;12:459-70. DOI:https://doi.org/10.1093/jpids/piad055

[197] Yin L, Lu L, He L, Lu G, Cao Y, Wang L, et al. Molecular characteristics of carbapenem-resistant gram-negative bacilli in pediatric patients in China. BMC microbiology. 2023;23:136. DOI:https://doi.org/10.1186/s12866-023-02875-0

[198] Yin L, Wu N, Yan G, Lu L, Qian H, Yang W, et al. Carbapenem-resistant gram-negative bacterial prevention practice in nosocomial infection and molecular epidemiological characteristics in a pediatric intensive care unit. Heliyon. 2023;9:e18969. DOI:https://doi.org/10.1016/j.heliyon.2023.e18969

[199] Zhang S, Mi P, Wang J, Li P, Luo K, Liu S, et al. The optimized carbapenem inactivation method for objective and accurate detection of carbapenemase-producing Acinetobacter baumannii. Frontiers in microbiology. 2023;14:1185450. DOI:https://doi.org/10.3389/fmicb.2023.1185450

[200] Zhao Y, Zhu Y, Zhang H, Zhang L, Li J, Ye Y. Molecular tracking of carbapenem-resistant Acinetobacter baumannii clinical isolates: a multicentre study over a 4-year period across eastern China. Journal of medical microbiology. 2023;72:DOI:https://doi.org/10.1099/jmm.0.001655

[201] Zou X, Jin S, Chen L, Li J, Zhang X, Zhou H, et al. Antibacterial Activity of Eravacycline Against Carbapenem-Resistant Gram-Negative Isolates in China: An in vitro Study. Infection and drug resistance. 2023;16:2271-9. DOI:https://doi.org/10.2147/IDR.S396910

[202] AlFaris EM, Al-Karablieh N, Odat NA, Rafei R. Carbapenem-resistant Acinetobacter baumannii from Jordan: Complicated Carbapenemase Combinations. Jordan Journal of Biological Sciences. 2024;17:355-61. DOI:https://doi.org/10.54319/jjbs/170216

[203] Arif M, Asif A, Nazeer K, Sultan S, Riaz S. Coexistence of β-lactamase genes and biofilm forming potential among carbapenem-resistant Acinetobacter baumannii in Lahore, Pakistan. Journal of infection in developing countries. 2024;18:943-9. DOI:https://doi.org/10.3855/jidc.19119

[204] Bae IK, Hong JS. The Distribution of Carbapenem-Resistant Acinetobacter Species and High Prevalence of CC92 OXA-23-Producing Acinetobacter Baumannii in Community Hospitals in South Korea. Infection and drug resistance. 2024;17:1633-41. DOI:https://doi.org/10.2147/IDR.S459739

[205] Baleivanualala SC, Matanitobua S, Soqo V, Smita S, Limaono J, Sharma SC, et al. Molecular and clinical epidemiology of carbapenem resistant Acinetobacter baumannii, Pseudomonas aeruginosa and Enterobacterales in Fiji: a multicentre prospective observational study. The Lancet regional health. Western Pacific. 2024;47:101095. DOI:https://doi.org/10.1016/j.lanwpc.2024.101095

[206] Bandić Pavlović D, Pospišil M, Nađ M, Vrbanović Mijatović V, Luxner J, Zarfel G, et al. Multidrug-Resistant Bacteria in Surgical Intensive Care Units: Antibiotic Susceptibility and β-Lactamase Characterization. Pathogens (Basel, Switzerland). 2024;13:DOI:https://doi.org/10.3390/pathogens13050411

[207] Bulens SN, Campbell D, McKay SL, Vlachos N, Burgin A, Burroughs M, et al. Carbapenem-resistant Acinetobacter baumannii complex in the United States-An epidemiological and molecular description of isolates collected through the Emerging Infections Program, 2019. American journal of infection control. 2024;52:1035-42. DOI:https://doi.org/10.1016/j.ajic.2024.04.184

[208] Carascal MB, Destura RV, Rivera WL. Molecular genotyping reveals multiple carbapenemase genes and unique bla(OXA-51-like) (oxaAb) alleles among clinically isolated Acinetobacter baumannii from a Philippine tertiary hospital. Tropical medicine and health. 2024;52:62. DOI:https://doi.org/10.1186/s41182-024-00629-w

[209] Chen PK, Lee YT, Liu CY, Thuy TTD, Anh K, Wu JJ, et al. A 19-year longitudinal study to characterize carbapenem-nonsusceptible Acinetobacter isolated from patients with bloodstream infections and the contribution of conjugative plasmids to carbapenem resistance and virulence. Journal of microbiology, immunology, and infection = Wei mian yu gan ran za zhi. 2024;57:288-99. DOI:https://doi.org/10.1016/j.jmii.2024.01.008

[210] Cureño-Díaz MA, Plascencia-Nieto ES, Loyola-Cruz M, Cruz-Cruz C, Nolasco-Rojas AE, Durán-Manuel EM, et al. Gram-Negative ESKAPE Bacteria Surveillance in COVID-19 Pandemic Exposes High-Risk Sequence Types of Acinetobacter baumannii MDR in a Tertiary Care Hospital. Pathogens (Basel, Switzerland). 2024;13:DOI:https://doi.org/10.3390/pathogens13010050

[211] Domingues R, Oliveira R, Silva S, Araújo D, Almeida C, Cho GS, et al. Molecular Detection of Carbapenemases in Acinetobacter baumannii Strains of Portugal and Association With Sequence Types, Capsular Types, and Virulence. Clinical therapeutics. 2024;DOI:https://doi.org/10.1016/j.clinthera.2024.09.005

[212] Dos Santos LA, Cayô R, Valiatti TB, Gales AC, de Araújo LFB, Rodrigues FM, et al. Biodiversity of carbapenem-resistant bacteria in clinical samples from the Southwest Amazon region (Rondônia/Brazil). Scientific reports. 2024;14:9383. DOI:https://doi.org/10.1038/s41598-024-59733-w

[213] Ekinci B, Sankur F, Turkyilmaz A, Citil BE, Aksozek A, Citil N. Comparison of Beta-Lactamase Profiles and Epidemiology of Multi-Drug Resistant Acinetobacter baumannii Isolates in Southwest Turkey: 2011 vs. 2019 Study. Jundishapur Journal of Microbiology. 2024;17:DOI:https://doi.org/10.5812/jjm-145756

[214] Fuentes-González MF, Fernández-Rodríguez D, Colín-Castro CA, Hernández-Durán M, López-Jácome LE, Franco-Cendejas R. Gram-Negative Bacilli Blood Stream Infection in Patients with Severe Burns: Microbiological and Clinical Evidence from a 9-Year Cohort. International journal of molecular sciences. 2024;25:DOI:https://doi.org/10.3390/ijms251910458

[215] Gurung A, Napit R, Shrestha B, Lekhak B. Carbapenem Resistance in Acinetobacter calcoaceticus-baumannii Complex Isolates From Kathmandu Model Hospital, Nepal, Is Attributed to the Presence of bla (OXA-23-like) and bla (NDM-1) Genes. BioMed research international. 2024;2024:8842625. DOI:https://doi.org/10.1155/2024/8842625

[216] Hernández-Chico I, Rodríguez-Guerrero E, Expósito-Ruiz M, Navarro-Marí JM, Gutiérrez-Fernández J. Reliability of a Screening Method Using Antibiotic Disks to Detect Carbapenemases in Glucose-Nonfermenting Gram-Negative Microorganisms From Clinical Samples of a Regional Hospital in Southeastern Spain. Journal of clinical laboratory analysis. 2024;38:e25036. DOI:https://doi.org/10.1002/jcla.25036

[217] Hsueh SC, Huang YT, Ko WC, Liu IM, Hsieh PC, Jean SS. In vitro antimicrobial susceptibility data of global meropenem-resistant Acinetobacter baumannii isolates causing pneumonia: Data from the Antimicrobial Testing Leadership and Surveillance Program, 2014-2021, and re-estimations of susceptibility breakpoints and appropriate dosages of important an. Journal of global antimicrobial resistance. 2024;36:411-8. DOI:https://doi.org/10.1016/j.jgar.2024.01.019

[218] Hu H, Wang Y, Sun J, Wang Y, Zhou J, Shi Q, et al. Risk factors and molecular epidemiology of intestinal colonization by carbapenem-resistant Gram-negative bacteria in patients with hematological diseases: a multicenter case‒control study. Microbiology spectrum. 2024;12:e0429923. DOI:https://doi.org/10.1128/spectrum.04299-23

[219] Jian X, Li Y, Wang H, Li C, Li F, Li J, et al. A comparative study of genotyping and antimicrobial resistance between carbapenem-resistant Klebsiella pneumoniae and Acinetobacter baumannii isolates at a tertiary pediatric hospital in China. Frontiers in cellular and infection microbiology. 2024;14:1298202. DOI:https://doi.org/10.3389/fcimb.2024.1298202

[220] Kazemian H, Karami-Zarandi M, Heidari H, Ghanavati R, Khoshnood S. Antimicrobial combination effects against multidrug-resistant Acinetobacter baumannii and Pseudomonas aeruginosa strains: A cross-sectional study. Health science reports. 2024;7:e2061. DOI:https://doi.org/10.1002/hsr2.2061

[221] Kovalchuk V, Kondratiuk V, McGann P, Jones BT, Fomina N, Nazarchuk O, et al. Temporal evolution of bacterial species and their antimicrobial resistance characteristics in wound infections of war-related injuries in Ukraine from 2014 to 2023. The Journal of hospital infection. 2024;152:99-104. DOI:https://doi.org/10.1016/j.jhin.2024.06.011

[222] Li YY, Liu HC, Wang HP, Du TY, Jiang L. [Characteristics of drug resistance and biofilm formation in carbapenem-resistant Acinetobacter baumannii in hospitalized children]. Zhongguo dang dai er ke za zhi = Chinese journal of contemporary pediatrics. 2024;26:358-64. DOI:https://doi.org/10.7499/j.issn.1008-8830.2309058

[223] Lukovic B, Kabic J, Dragicevic M, Kuljanin S, Dimkic I, Jovcic B, et al. Genetic basis of antimicrobial resistance, virulence features and phylogenomics of carbapenem-resistant Acinetobacter baumannii clinical isolates. Infection. 2024;DOI:https://doi.org/10.1007/s15010-024-02316-8

[224] Luo TL, Martin MJ, Kovalchuk V, Kondratiuk V, Trapaidze N, Metreveli M, et al. Detection of carbapenemase producing Acinetobacter baumannii ST19 from Georgia and Ukraine carrying bla (OXA-23), bla (OXA-72), and/or bla (NDM-5), December 2019 to June 2023. Euro surveillance : bulletin Europeen sur les maladies transmissibles = European communicable disease bulletin. 2024;29:DOI:https://doi.org/10.2807/1560-7917.ES.2024.29.24.2400259

[225] Marusinec R, Shemsu M, Lloyd T, Kober BM, Heaton DT, Herrera JA, et al. Epidemiology of carbapenem-resistant organisms in Alameda County, California, 2019-2021. Antimicrobial stewardship & healthcare epidemiology : ASHE. 2024;4:e64. DOI:https://doi.org/10.1017/ash.2024.33

[226] Morales L, Cobo A, Frías MP, Gálvez A, Ortega E. The Prevalence of Antibiotic Resistance Phenotypes and Genotypes in Multidrug-Resistant Bacterial Isolates from the Academic Hospital of Jaén, Spain. Antibiotics (Basel, Switzerland). 2024;13:DOI:https://doi.org/10.3390/antibiotics13050429

[227] Özdede M, Zarakolu P, Metan G, Köseoğlu Eser Ö, Selimova C, Kızılkaya C, et al. Predictive modeling of mortality in carbapenem-resistant Acinetobacter baumannii bloodstream infections using machine learning. Journal of investigative medicine : the official publication of the American Federation for Clinical Research. 2024;72:684-96. DOI:https://doi.org/10.1177/10815589241258964

[228] Papadopoulou M, Deliolanis I, Polemis M, Vatopoulos A, Psichogiou M, Giakkoupi P. Characteristics of the Genetic Spread of Carbapenem-Resistant Acinetobacter baumannii in a Tertiary Greek Hospital. Genes. 2024;15:DOI:https://doi.org/10.3390/genes15040458

[229] Rocha IV, Martins LR, Pimentel MIS, Mendes RPG, Lopes ACS. Genetic profile and characterization of antimicrobial resistance in Acinetobacter baumannii post-COVID-19 pandemic: a study in a tertiary hospital in Recife, Brazil. Journal of applied microbiology. 2024;135:DOI:https://doi.org/10.1093/jambio/lxae148

[230] Rodríguez-Aguirregabiria M, Lázaro-Perona F, Cacho-Calvo JB, Arellano-Serrano MS, Ramos-Ramos JC, Rubio-Mora E, et al. Challenges Facing Two Outbreaks of Carbapenem-Resistant Acinetobacter baumannii: From Cefiderocol Susceptibility Testing to the Emergence of Cefiderocol-Resistant Mutants. Antibiotics (Basel, Switzerland). 2024;13:DOI:https://doi.org/10.3390/antibiotics13080784

[231] Sabour S, Bantle K, Bhatnagar A, Huang JY, Biggs A, Bodnar J, et al. Descriptive analysis of targeted carbapenemase genes and antibiotic susceptibility profiles among carbapenem-resistant Acinetobacter baumannii tested in the Antimicrobial Resistance Laboratory Network-United States, 2017-2020. Microbiology spectrum. 2024;12:e0282823. DOI:https://doi.org/10.1128/spectrum.02828-23

[232] Saikia S, Gogoi I, Oloo A, Sharma M, Puzari M, Chetia P. Co-production of metallo-β-lactamase and OXA-type β-lactamases in carbapenem-resistant Acinetobacter baumannii clinical isolates in North East India. World journal of microbiology & biotechnology. 2024;40:167. DOI:https://doi.org/10.1007/s11274-024-03977-1

[233] Sakuma M, Tohya M, Hishinuma T, Sherchand JB, Kirikae T, Tada T. Molecular epidemiology of multidrug-resistant Acinetobacter baumannii isolates from a hospital in Nepal. Journal of global antimicrobial resistance. 2024;38:363-7. DOI:https://doi.org/10.1016/j.jgar.2024.07.017

[234] Selim MI, Sonbol FI, El-Banna TE, Negm WA, Elekhnawy E. Antibacterial and wound healing potential of biosynthesized zinc oxide nanoparticles against carbapenem-resistant Acinetobacter baumannii: an in vitro and in vivo study. Microbial cell factories. 2024;23:281. DOI:https://doi.org/10.1186/s12934-024-02538-3

[235] Serwacki PA, Hareza DA, Kujawska A, Pałka A, Jachowicz-Matczak E, Rybka-Grymek A, et al. Molecular epidemiology and clinical significance of carbapenemase genes in carbapenem-resistant Acinetobacter baumannii isolates in southern Poland. Polish archives of internal medicine. 2024;134:DOI:https://doi.org/10.20452/pamw.16734

[236] Sharma V, Sharma R, Vyas A. Molecular Identification of Carbapenem Resistance Acinetobacter baumannii causing Ventilator-Associated Pneumonia Isolated from Intensive Care Unit of Tertiary Care Hospital. Journal of Pure and Applied Microbiology. 2024;18:696-702. DOI:https://doi.org/10.22207/JPAM.18.1.59

[237] Soria-Segarra C, Soria-Segarra C, Molina-Matute M, Agreda-Orellana I, Núñez-Quezada T, Cevallos-Apolo K, et al. Molecular epidemiology of carbapenem-resistant gram-negative bacilli in Ecuador. BMC infectious diseases. 2024;24:378. DOI:https://doi.org/10.1186/s12879-024-09248-6

[238] Sotomayor N, Villacis JE, Burneo N, Reyes J, Zapata S, Bayas-Rea R. Carbapenemase genes in clinical and environmental isolates of Acinetobacter spp. from Quito, Ecuador. PeerJ. 2024;12:e17199. DOI:https://doi.org/10.7717/peerj.17199

[239] Tran LS, Thi NNP, Thi BT, Phan MH. Epidemiology and antibiotic resistance assessment of Acinetobacter baumannii isolates from respiratory specimens collected at Can Tho General Hospital. Journal of Applied Biology and Biotechnology. 2024;12:198-204. DOI:https://doi.org/10.7324/JABB.2024.146101

[240] Uskudar-Guclu A, Danyildiz S, Mirza HC, Akcil Ok M, Basustaoglu A. In vitro activity of cefiderocol against carbapenem-resistant Acinetobacter baumannii carrying various β-lactamase encoding genes. European journal of clinical microbiology & infectious diseases : official publication of the European Society of Clinical Microbiology. 2024;43:1171-9. DOI:https://doi.org/10.1007/s10096-024-04831-w

[241] Vintilă C, Coșeriu RL, Mare AD, Ciurea CN, Togănel RO, Simion A, et al. Biofilm Formation and Antibiotic Resistance Profiles in Carbapenemase-Producing Gram-Negative Rods-A Comparative Analysis between Screening and Pathological Isolates. Antibiotics (Basel, Switzerland). 2024;13:DOI:https://doi.org/10.3390/antibiotics13080687

[242] Wang L, Chen QW, Qin YC, Yi XL, Zeng H. Analysis of carbapenem-resistant Acinetobacter baumannii carbapenemase gene distribution and biofilm formation. International journal of molecular epidemiology and genetics. 2024;15:1-11. DOI:https://doi.org/10.62347/KBSB9946

[243] Wang M, Ge L, Chen L, Komarow L, Hanson B, Reyes J, et al. Clinical Outcomes and Bacterial Characteristics of Carbapenem-resistant Acinetobacter baumannii Among Patients From Different Global Regions. Clinical infectious diseases : an official publication of the Infectious Diseases Society of America. 2024;78:248-58. DOI:https://doi.org/10.1093/cid/ciad556

[244] Worku S, Abebe T, Seyoum B, Alemu B, Denkayehu G, Seyoum T, et al. Molecular characterization of carbapenemase and extended spectrum beta-lactamase producing Acinetobacter baumannii isolates causing surgical site infections in Ethiopia. BMC infectious diseases. 2024;24:459. DOI:https://doi.org/10.1186/s12879-024-09362-5

[245] Wu Y, Chen J, Zhang G, Li J, Wang T, Kang W, et al. In-vitro activities of essential antimicrobial agents including aztreonam/avibactam, eravacycline, colistin and other comparators against carbapenem-resistant bacteria with different carbapenemase genes: A multi-centre study in China, 2021. International journal of antimicrobial agents. 2024;64:107341. DOI:https://doi.org/10.1016/j.ijantimicag.2024.107341

[246] Xiu Y, Dai Y, Yin S, Wei Q. Analysis of the Class 1 Integrons, Carbapenemase Genes and Biofilm Formation Genes Occurrence in Acinetobacter baumannii Clinical Isolates. Polish journal of microbiology. 2024;73:189-97. DOI:https://doi.org/10.33073/pjm-2024-017

[247] Xu A, Li M, Hang Y, Zeng L, Zhang X, Hu Y, et al. Multicenter retrospective genomic characterization of carbapenemase-producing Acinetobacter baumannii isolates from Jiangxi patients 2021-2022: identification of a novel international clone, IC11. mSphere. 2024;9:e0027624. DOI:https://doi.org/10.1128/msphere.00276-24

[248] Yao Y, Imirzalioglu C, Falgenhauer L, Falgenhauer J, Heinmüller P, Domann E, et al. Plasmid-Mediated Spread of Carbapenem Resistance in Enterobacterales: A Three-Year Genome-Based Survey. Antibiotics (Basel, Switzerland). 2024;13:DOI:https://doi.org/10.3390/antibiotics13080682

[249] Yek C, Lon C, Chea S, Lay S, Oum MH, Tang G, et al. Bacterial Genomics for National Antimicrobial Resistance Surveillance in Cambodia. The Journal of infectious diseases. 2024;DOI:https://doi.org/10.1093/infdis/jiae417
